# Tailoring electric arc furnace steelmaking expansion pathways based on regional scrap and energy availability in China

**DOI:** 10.1016/j.isci.2026.117512

**Published:** 2026-09-12

**Authors:** Jialin Shen, Shuoshuo Tian, Zhao Zhang, Zhaoling Li, Hongyou Lu, Peng Wang, Lu Sun, Linyang Wei, Sheng Xie, Qi Zhang

**Affiliations:** 1 Engineering Research Center of Ministry of Education for Frontier Technologies of Low-Carbon Steelmaking, Shenyang, Liaoning 110819, China; 2 Department of Thermal Engineering, School of Metallurgy, Northeastern University, Shenyang, Liaoning 110819, China; 3 School of Economics, Shanghai University, Shanghai 200444, China; 4 Lawrence Berkeley National Laboratory, Berkeley, CA 94720, USA; 5 Key Lab of Urban Environment and Health, Institute of Urban Environment, Chinese Academy of Sciences, Xiamen, China; 6 School of Human Settlements and Civil Engineering, Xi’an Jiaotong University, Xi’an 710049, China; 7 State Environmental Protection Key Laboratory of Eco-Industry, Northeastern University, Shenyang, Liaoning 110819, China

**Keywords:** electric arc furnace, steelmaking, scrap, regional disparities, low-carbon electricity, decarbonization

## Abstract

Electric arc furnace (EAF) steelmaking underpins China’s low-carbon steel transition, while uneven regional resources and energy conditions greatly constrain its low-carbon development. This study adopts a province-level material flow analysis-capacity integration framework with multi-scenario modeling to explore targeted regional decarbonization pathways. Although national scrap supply generally meets domestic EAF demand, severe provincial mismatches occur: major producers, including Hebei (36.3 Mt) and Guangdong (32.0 Mt), heavily rely on interprovincial scrap transfers. Low-carbon electricity-rich regions are spatially separated from core EAF hubs, driving large disparities in provincial electricity carbon intensity (0.101–0.171 kg CO_2_ kWh^−1^ by 2060) and heterogeneous EAF emissions ranging from 69.6 to 92.7 Mt across scenarios. This study highlights dynamic scrap circulation, renewable energy matching, and differentiated provincial policies to coordinate national steel decarbonization.

## Highlights

• National scrap supply is sufficient, but regional scrap and EAF capacity mismatches
• Spatial separation of renewable resources and EAF restricts steel decarbonization
• Provincial CO_2_ emissions vary with production dynamics and electricity mixes
• Coordinated policies can mitigate regional gaps and advance steel decarbonization

## Graphical abstract

**Figure:**
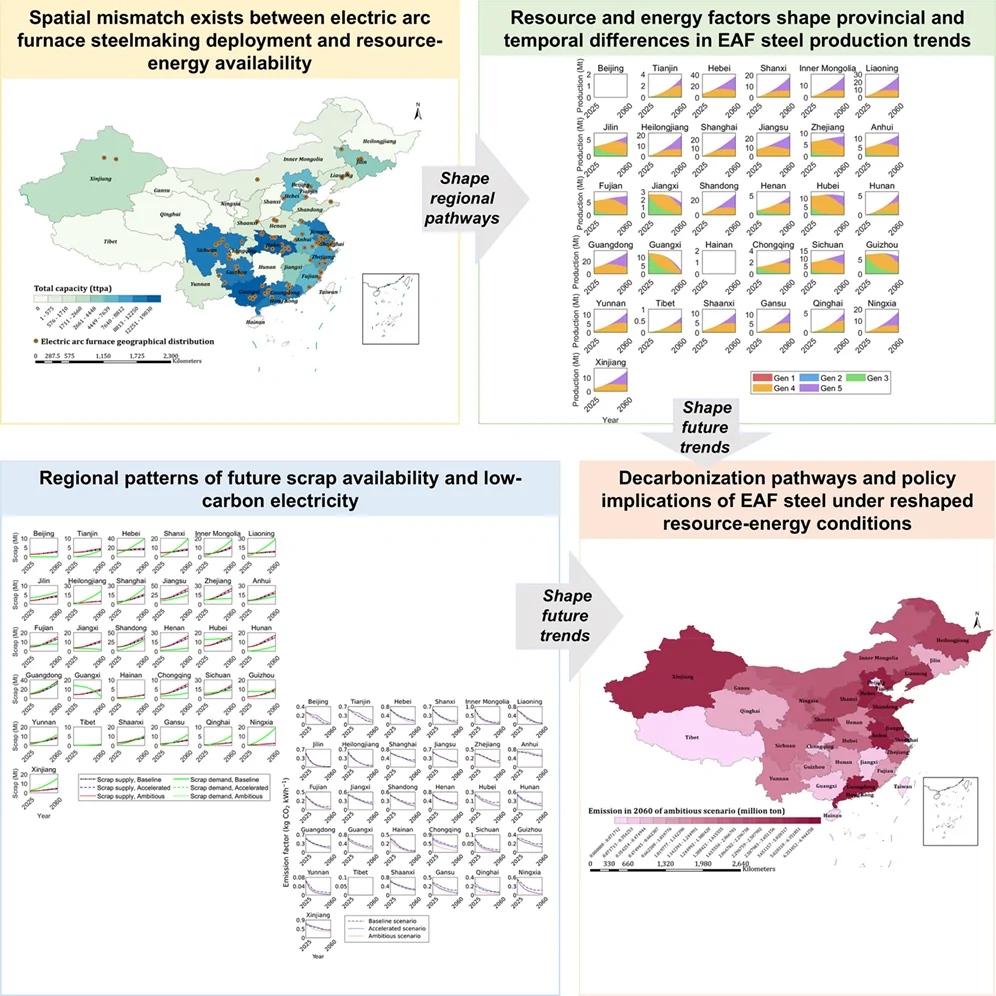

## Teaser

Industrial engineering; Manufacturing engineering

## Introduction

The global steel sector accounts for approximately 7% of CO_2_ emissions, posing a critical challenge for achieving net-zero targets.^1^ As the world’s largest steel producer, China accounted for over 54% of global crude steel production in 2024,^2^ and its steel output remains dominated by the carbon-intensive blast furnace-basic oxygen furnace (BF-BOF) route.^3^ An electric arc furnace (EAF) emits just 0.70 t CO_2_ per ton of steel—far lower than the 2.32 t CO_2_/t steel from the BF-BOF route.^4^ Transitioning from this conventional pathway to EAF steelmaking has been widely recognized as a key decarbonization strategy.^5^^,^^6^^,^^7^ In China, accelerating EAF deployment has become an important component of the national “dual-carbon” strategy.^8^ Recent policy documents, including the Guiding Opinions on Promoting High-Quality Development of the Iron and Steel Industry and the Implementation Plan for Carbon Peaking in the Industrial Sector, explicitly identify EAF expansion, scrap utilization, and clean-energy integration as key pathways for steel-sector decarbonization.^9^^,^^10^ EAF offers inherent advantages in energy efficiency, scrap recycling, and compatibility with low-carbon electricity.^11^^,^^12^ The technology is well established and demonstrates stable industrial-scale operation. It also remains economically viable, with its cost being less than 15% higher than that of BF-BOF steel.^13^ Looking ahead, China’s EAF steelmaking capacity is projected to expand significantly. According to national development plans, the share of EAF in China’s crude steel output is expected to increase to about 15% by 2025, while longer-term expectations from industry and academic studies suggest that this share could rise further to 60–80% by 2050–2060.^14^^,^^15^ EAF steel production is expected to grow several-fold by 2060, driven by a surge in scrap supply—a direct result of the retirement of steel products from decades of high crude steel output—alongside rising demand for low-carbon steel in construction, manufacturing, and transport.^16^^,^^17^ This trend underscores a fundamental structural shift in China’s steel industry.

The feasibility of EAF steelmaking expansion fundamentally depends on the balance between the supply of upstream resources—primarily scrap steel and electricity—and the corresponding demand from EAF production.^18^ On the demand side, expanding EAF capacity will significantly increase electricity consumption. Given that China’s shift toward EAFs is closely tied to its broader decarbonization strategy, this transition also heightens the need for low-carbon electricity and high-quality scrap to ensure that the electrification of steelmaking delivers the intended emissions reductions.^19^^,^^20^ Therefore, the decarbonization potential of EAF steelmaking depends not only on the deployment of EAF technology itself, but also on the coordinated availability of scrap resources and the progress of power system decarbonization. These factors jointly determine whether EAF expansion can deliver substantial long-term emission reductions.^21^ The scale of this resource demand is ultimately shaped by macro-level socioeconomic drivers such as population growth, GDP expansion, and urbanization, which influence the trajectory of steel production and, consequently, the required inputs for EAF processes.^22^ On the supply side, the decarbonization benefits of EAF steelmaking depend largely on the carbon intensity of the electricity used, which varies across provinces and is expected to decline over time with the growth of renewable energy.^23^^,^^24^ Scrap supply derives from three sources with distinct spatiotemporal patterns: (1) end-of-life (EOL) scrap from retired steel products (building/machine/appliance/automobile/power-sectors), emerging decades later at consumption sites; (2) home scrap from steelmaking byproducts at mills (e.g., furnace returns or offcuts from rolling), generated immediately during production; and (3) prompt scrap from manufacturing waste (e.g., stamping scraps) at processing plants—collectively creating complex supply variability.^11^^,^^25^^,^^26^ In addition to different categories of scrap, direct reduced iron (DRI) produced from iron-bearing materials via rotary hearth furnaces (RHF) serves as a major complementary raw material for EAF operations, and the techno-economic performance and life cycle environmental impacts of this coupled process have been extensively studied in existing literature.^27^^,^^28^ These interrelated dynamics call for an integrated assessment of energy system transitions and scrap flow projections to evaluate the long-term viability of EAF expansion under different regional conditions.

Furthermore, national ambitions to accelerate low-carbon steel development coexist with significant heterogeneity across Chinese provinces—in resource endowments, electricity mix, industrial structure, and scrap availability—leading to highly differentiated potentials for EAF deployment.^17^^,^^29^^,^^30^^,^^31^ Provinces such as Hebei, with a strong BF-BOF infrastructure and limited access to clean electricity, may prioritize process upgrades and adopt the EAF routes with hot metal.^32^ In contrast, Jiangsu and Guangdong, with robust manufacturing sectors and abundant scrap resources, are better positioned to expand EAF-based production using recycled materials.^33^ Meanwhile, provinces like Inner Mongolia can harness their renewable energy advantages to support greenfield EAF projects.^34^ These diverse local conditions shape distinct transition pathways and underscore the limitations of one-size-fits-all strategies. Moreover, because future EAF deployment will occur at the provincial level, spatial mismatches between scrap availability, low-carbon electricity supply, and steel production demand may become critical constraints on achieving national EAF expansion targets.^35^ Understanding provincial heterogeneity is therefore essential for developing realistic long-term deployment pathways at the national scale. To inform realistic and equitable decarbonization planning, province-level analyses that consider local techno-economic conditions, resource and energy constraints, and decarbonization potentials are essential. A spatially resolved approach enables tailored policy recommendations that align with regional characteristics while collectively contributing to China’s national carbon neutrality goals.^36^

Existing studies on EAF development and steel-sector decarbonization have primarily focused on national-scale assessments, technology deployment potential, economic performance, or emission reduction benefits.^19^^,^^37^^,^^38^^,^^39^ In addition, studies addressing scrap availability and electricity decarbonization have often examined these factors separately.^11^^,^^24^ However, the future feasibility of EAF expansion depends on the coordinated evolution of scrap supply, power-sector transition, and production capacity deployment. Few studies have integrated these interrelated dimensions within a unified provincial-level analytical framework. Recent province-level studies have significantly advanced the understanding of regional steel transitions by examining the evolution of steel stocks and scrap resources, the spatial reconfiguration of steel production systems, and the coordination of interprovincial resource flows for decarbonization.^17^^,^^30^^,^^33^ However, these studies generally focus on specific aspects of the transition process and provide limited detail on the interactions among heterogeneous scrap supply streams, evolving power-sector decarbonization pathways, and EAF deployment. Given the strong interdependence among these factors, a more integrated framework is needed to identify province-specific constraints and opportunities for long-term EAF expansion. To address this gap, this study further incorporates detailed scrap supply-demand dynamics across end-use sectors and scrap types together with provincial power-sector decarbonization pathways into a unified material flow analysis (MFA)-capacity integration framework.

Moreover, unlike earlier studies that forecast EAF steel demand merely based on socio-economic indicators such as population and economic growth, or traditional MFA studies limited to estimating scrap generation and availability, this MFA-capacity integration framework further centers on the evolution of provincial EAF capacity.^40^^,^^41^ It systematically couples local scrap supply and power-sector conditions into one unified model, explicitly connecting scrap flows, EAF capacity dynamics, electricity demand, and power decarbonization. This design supports dynamic evaluation of resource-capacity matching and regional constraints, and delivers solid evidence for formulating province-specific EAF development plans and decarbonization strategies.

Here, this study presents a comprehensive, province-level assessment of resource and energy availability and supply-demand dynamics for EAF development in China, applying a province-level MFA-capacity integration framework combined with scenario-based modeling to quantify regional low-carbon transition pathways. First, this study estimated provincial EAF capacity and production from 2025 to 2060, considering deployments across five representative EAF types, differentiated by their technological features. Second, the provincial scrap supply-demand balance by estimating future scrap generation from different scrap types and end-use sectors based on stock-driven dynamic MFA is assessed. Third, the evolution of each province’s power structure and associated electricity emission factors, accounting for planned changes in energy mix and decarbonization efforts in the power-sector is forecasted. Finally, total CO_2_ emissions from EAF—covering both direct and indirect sources—are estimated, and these results are used to propose differentiated development pathways for each province is estimated. This assessment aims to evaluate the future alignment between scrap and electricity supply and EAF steelmaking demand in each province, thereby enabling the formulation of province-specific decarbonization timelines and roadmaps for the steel sector.

## Results

### Provincial distribution of EAFs in China

**Figure 1:**
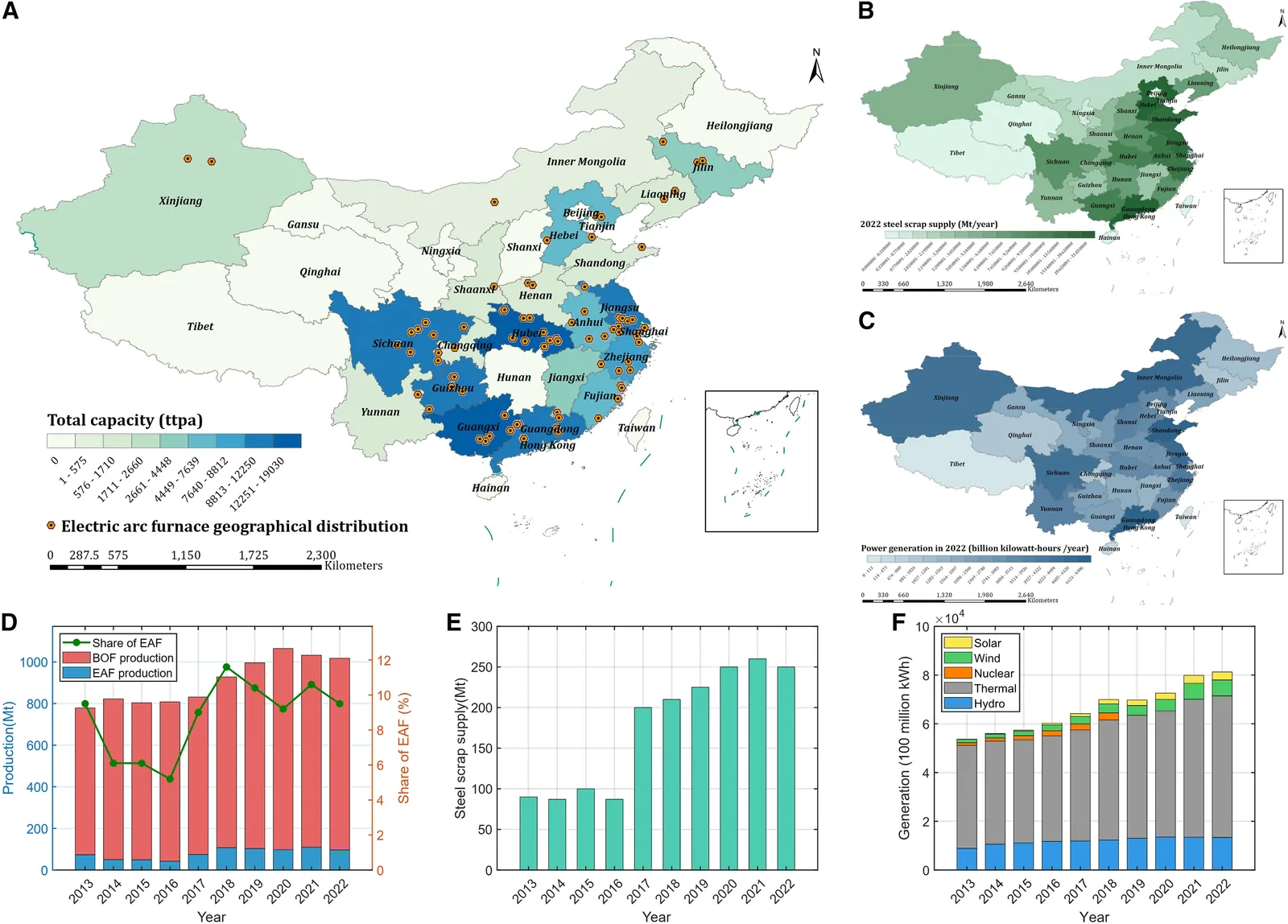
The distribution and characteristics of electric arc furnace steel equipment and its resources and energy (A) Geographic distribution of electric arc furnace (EAF) equipment and installed EAF steelmaking capacity across provinces. (B) Provincial scrap steel supply. (C) Provincial electricity generation. (D) Historical EAF and basic oxygen furnace (BOF) steel production. (E) Historical scrap steel supply. (F) Historical electricity generation and the composition of the electricity mix (stacked-area representation).

China’s EAF steel has experienced gradual growth over the past decades.^35^^,^^42^ The distribution and characteristics of EAF steel equipment and its resources and energy are illustrated in Figures 1A–1F.^2^^,^^43^^,^^44^ From 2013 to 2022, national EAF steel production increased from 74.0 to 96.7 Mt. Regional differences have been significant, with provinces such as Jiangsu and Guangdong leading in early adoption.^45^ The present distribution of EAF equipment across China is highly uneven. Provinces like Guangdong, Sichuan, Hubei, and Jiangsu host the highest concentrations of EAFs, and the eastern and central regions dominate EAF deployment, whereas western and less industrialized regions lag behind (Figure 1A). In regions such as Jiangsu and Guangdong, the abundant availability of scrap not only ensured a stable material supply but also significantly reduced feedstock costs relative to other provinces, making EAF production more economically attractive and accelerating local EAF deployment. Similarly, Sichuan benefits from abundant low-carbon hydropower and comparatively low electricity prices, which further enhance the competitiveness of EAF operations in the region.

Scrap steel, as the most important iron-bearing raw material in EAF steel production, plays a critical role in determining production capacity and efficiency.^46^^,^^47^ The annual supply of scrap steel in China has shown significant growth over the past decade. From 2013 to 2016, the national scrap supply remained relatively stable at around 80–100 Mt, but after 2017, it increased sharply from approximately 80 Mt to around 200 Mt. Despite this substantial growth in scrap availability, the concurrent rapid expansion of EAF steel production has led to a persistent shortage of scrap relative to demand. Furthermore, the geographic distribution of scrap resources does not align with the distribution of EAF equipment. Most EAF facilities are concentrated in the eastern and central provinces, while the largest scrap resources are located in provinces with higher traditional BF-BOF steel production, high steel consumption and higher population densities, such as Hebei, Shandong, Jiangsu, and Guangdong (Figure 1B). This spatial mismatch between scrap availability and EAF location creates constrains efficient EAF steel production, potentially limiting the ability to fully utilize installed EAF capacity and necessitating the transportation of scrap over long distances.^48^

Electricity supply is a crucial factor for the operation and expansion of EAF steel production. However, the geographic distribution of electricity generation does not align well with the location of EAF facilities. Provinces with high total electricity output, such as Inner Mongolia, Xinjiang, Shandong, and Guangdong, do not fully coincide with the major EAF steel provinces in the eastern and central regions. Moreover, the low-carbon transformation of steel production requires access to clean electricity, including hydro, wind, solar, and nuclear power, to reduce the carbon emissions of EAF operations. Provinces with abundant clean electricity resources, such as Inner Mongolia, Sichuan, and Yunnan, are often spatially separated from the largest concentrations of EAF equipment (Figure 1C).^24^^,^^49^^,^^50^ This spatial mismatch between clean electricity availability and EAF location poses a challenge for achieving low-carbon steel production, indicating that future expansion of EAF capacity may need to consider both proximity to electricity supply and the share of renewable generation.^34^^,^^51^

### Scrap resource mismatch patterns for EAF expansion

**Figure 2:**
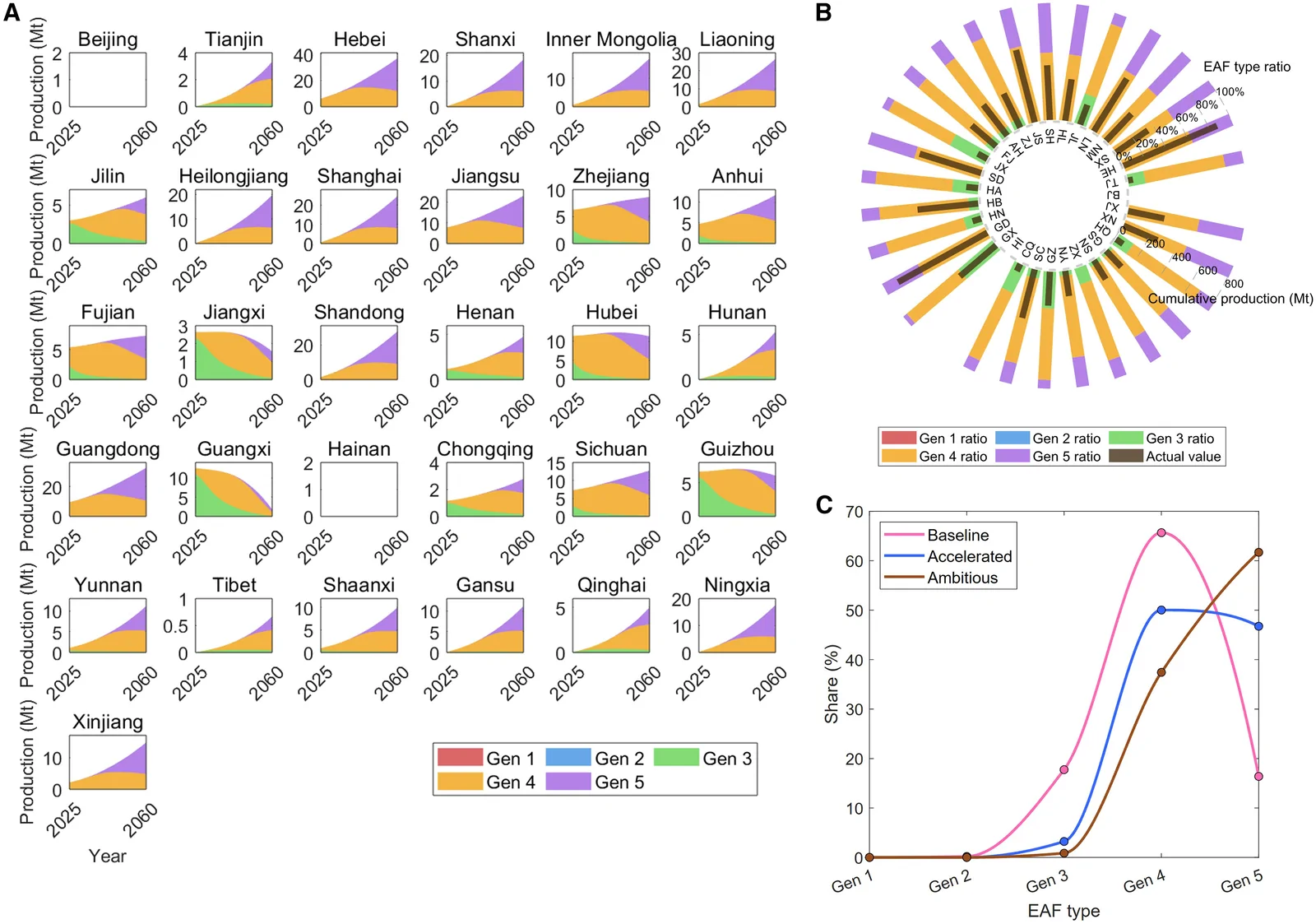
Evolution of provincial EAF steel production under different scenarios (A) Time-series trajectories of provincial EAF steel production by technology type in the ambitious scenario, shown across 31 provincial panels (Beijing, Tianjin, Hebei, Shanxi, Inner Mongolia, Liaoning, Jilin, Heilongjiang, Shanghai, Jiangsu, Zhejiang, Anhui, Fujian, Jiangxi, Shandong, Henan, Hubei, Hunan, Guangdong, Guangxi, Hainan, Chongqing, Sichuan, Guizhou, Yunnan, Tibet, Shaanxi, Gansu, Qinghai, Ningxia, and Xinjiang). (B) Cumulative provincial contributions of different EAF technology types in the ambitious scenario (stacked bars). The legend defines Gen1–5 ratio as EAF type ratio and actual value as actual cumulative production value. Space limitations necessitate the use of provincial abbreviations here: BJ, Beijing; TJ, Tianjin; HE, Hebei; SX, Shanxi; NM, Inner Mongolia; LN, Liaoning; JL, Jilin; HL, Heilongjiang; SH, Shanghai; JS, Jiangsu; ZJ, Zhejiang; AH, Anhui; FJ, Fujian; JX, Jiangxi; SD, Shandong; HA, Henan; HB, Hubei; HN, Hunan; GD, Guangdong; GX, Guangxi; HI, Hainan; CQ, Chongqing; SC, Sichuan; GZ, Guizhou; YN, Yunnan; XZ, Tibet; SN, Shaanxi; GS, Gansu; QH, Qinghai; NX, Ningxia; and XJ, Xinjiang. (C) National EAF steel production by technology type under multiple scenarios in 2060 (line plots).

EAF steelmaking has emerged as a crucial pathway for decarbonizing China’s steel industry, and assessing its future development at the provincial level is essential for guiding region-specific strategies.^30^^,^^52^ To further analyze the development potentials of EAF, this study projects provincial EAF steel production from 2025 to 2060 under three scenarios: baseline, accelerated, and ambitious. The study develops a provincial-level EAF trajectory model that integrates technological differentiation, equipment lifetimes, and capacity utilization rates, thereby capturing both the scale and structural evolution of EAF development across provinces. Figures 2A–2C illustrates the evolution trends of EAF steel production in different scenarios. Under the baseline scenario, the national EAF steel production amounts to 375 Mt in 2060. Approximately 49.8% of output is produced by EAFs that adopt key process-efficiency improvements, including oxygen burners, scrap pre-heating upgrades, and high-efficiency transformers. Another 44.9% of the output derives from EAFs that further integrate digital process-control and automation functions, such as real-time operational optimization and predictive energy-management systems. Several major steel-producing provinces—Guangdong (32.0 Mt), Hebei (36.3 Mt), and Jiangsu (22.7 Mt)—show the highest cumulative production in 2060, reflecting their large traditional steelmaking base and the gradual transition of BF-BOF capacity toward EAF steelmaking. Provinces with abundant clean electricity resources also demonstrate strong EAF development potential, such as Sichuan, with 12.5 Mt of cumulative production. Under the accelerated scenario, EAF steel production reaches 379 Mt in 2060, slightly higher than the baseline scenario. Although the total increase is modest, the accelerated scenario drives a more pronounced structural shift toward EAFs equipped with advanced process, efficiency, and digital-control improvements. At the national level, EAFs incorporating digitalized and automation-oriented features account for 50.0% of cumulative output. In several major steel-producing provinces, the transition occurs at an even faster pace than the national average—for instance, in Sichuan Province, digitally enhanced EAFs are projected to contribute 56.90% of total cumulative production. This reflects the impact of faster technology deployment and strengthened policy support for electrification and low-carbon transformation. In the ambitious scenario, which assumes rapid adoption of advanced EAF technologies and substantial replacement of BF-BOF steelmaking by the EAF routes, the dominance of advanced EAF technologies becomes even more pronounced. By 2060, EAFs integrating low-carbon energy use, high-efficiency process configurations, and advanced digital control systems together represent 99.1% of national EAF production, reaching 381 Mt. This outcome highlights the transformative role of aggressive decarbonization policies and the accelerated penetration of advanced EAF technologies.

**Figure 3:**
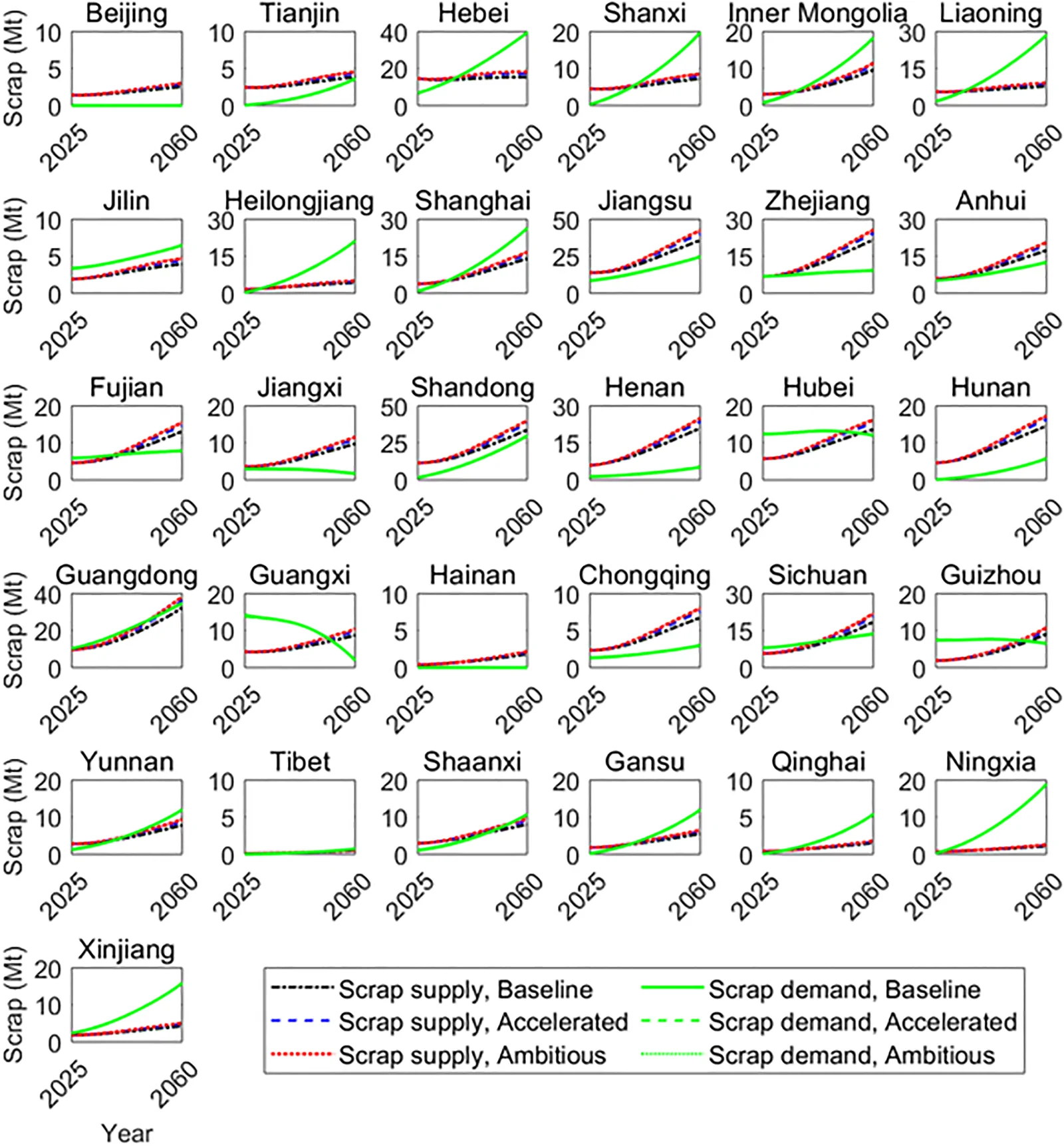
Provincial trajectories of steel scrap supply and demand across scenarios Each image represents one of China’s 31 provinces. For each province, three curves show projected scrap supply under the baseline, accelerated, and ambitious scenarios, and three additional curves show the corresponding scrap demand trajectories. Together, these images illustrate the evolving balance between scrap supply and demand across provinces under alternative development pathways.

Scrap resources are the cornerstone for sustaining EAF production, and therefore analyzing the supply-demand balance of scrap is of critical importance.^53^ The provincial scrap supply-demand relationships are illustrated in Figure 3. Based on the projected EAF steel output, scrap steel demand in 2060 is estimated at 408–411 Mt under different scenarios, reflecting modest differences in production levels while assuming an input-output ratio of 1.07–1.30 t scrap per ton of steel in different types of EAF equipment. On the supply side, the sources of scrap steel are diverse, namely home scrap, prompt scrap, and EOL scrap, with their respective contributions quantified by quality. Home scrap and prompt scrap are characterized by high recycling efficiency but relatively small quantities, whereas EOL scrap has lower recovery efficiency but constitutes the largest potential source, with diverse recovery channels across industries.^54^ Provincial simulations show that domestic and processing scrap decline steadily, reaching 25.1 and 22.1 Mt respectively nationally by 2060 because they closely follow crude steel production and apparent consumption. The scrap from the construction, machinery, and power-sectors continues to rise, with Jiangsu and Guangdong leading in construction and machinery scrap, and Sichuan and Inner Mongolia in power-sector scrap. In contrast, scrap from appliances and automobiles shows mixed patterns, increasing in some provinces but peaking and then declining in others due to demand saturation and population decline. The relationship between scrap demand and supply reveals three key findings. First, the demand for scrap shows only limited differences across scenarios. This is because, although the structure of EAF technologies evolves rapidly over time, total EAF steel output is primarily determined by the same exogenous socioeconomic parameters in each scenario, resulting in similar overall demand. Moreover, technological iteration mainly improves smelting duration and power consumption, but has only marginal effects on reducing the requirement for scrap input. Second, scrap supply exhibits more significant differences across scenarios, reflecting differences in scrap recovery and recycling performance. By 2060, recovery rates are assumed to improve progressively across scenarios, with the ambitious scenario achieving the highest rates for all scrap grades, with high-quality scrap projected to reach around 95%, followed by the accelerated and baseline scenarios, which demonstrate the divergent trends of scrap supply driven by varying recycling performance across scenarios.

The provincial mismatch patterns can be grouped into four types. (1) Persistent-surplus regions such as Beijing, Tianjin, Jiangsu, Zhejiang, Anhui, Jiangxi, Shandong, Henan, Hunan, Hainan, and Chongqing consistently generate more scrap than they consume, driven either by strong recycling bases (e.g., Jiangsu, Shandong) or by high obsolete scrap inflows despite limited future EAF deployment (e.g., Beijing, Hainan). These provinces should facilitate cross-provincial scrap circulation to neighboring deficit areas such as Hebei, Shanghai, and Guangdong. (2) Persistent-deficit regions such as Jilin and Xinjiang face chronic shortages. Jilin’s deficit can be bridged through regional transfers, whereas Xinjiang’s structural gap requires broader measures, including large dismantling facilities and incentives for recycling enterprises. (3) Early-surplus-late-deficit regions including Hebei, Shanxi, Inner Mongolia, Liaoning, Heilongjiang, Shanghai, Yunnan, Tibet, Shaanxi, Gansu, Qinghai, and Ningxia experience declining supply over time. These regions should strengthen recycling systems and develop interprovincial logistics channels before shortages emerge. (4) Early-deficit-late-surplus regions such as Fujian, Hubei, Guangdong, Guangxi, Sichuan, and Guizhou exhibit rising scrap availability over time. Short-term deficits must be met through inflows, while long-term policies should focus on upgrading sorting and storage capacities to utilize emerging surpluses.

Overall, although provincial scrap supply varies considerably, at the national scale the resource base is broadly sufficient to support the future expansion of EAF steelmaking. By 2060, the projected scrap supply of 356–422 Mt is largely able to meet the EAF steel output of 375–381 Mt. The key lies in enabling dynamic interprovincial scrap flows to achieve resource complementarity. In this regard, categories (1) and (2) exhibit mutually reinforcing supply-demand balances, while categories (3) and (4) also form a complementary pair. Specifically, early surpluses in group (3) provinces can alleviate initial deficits in group (4) provinces, while later surpluses in group (4) provinces can compensate for emerging shortages in group (3) provinces. To operationalize this complementarity, a phased scrap reallocation mechanism should be established. In the early stage (2025–2040), northern and western provinces such as Hebei, Shanxi, and Inner Mongolia should channel surplus scrap to high-demand but deficit provinces like Guangdong, Fujian, and Sichuan. In the later stage (2040–2060), as southern and southwestern provinces including Guangdong, Sichuan, and Guangxi accumulate surpluses, they should redirect resources to deficit provinces such as Liaoning, Shaanxi, and Gansu. Building such dynamic “north-south” and “east-west” scrap circulation corridors, combined with differentiated policies—surplus provinces focusing on circulation and quality upgrading, persistent-deficit provinces on external sourcing and recycling expansion, and transitional provinces on phased coordination-will ensure sustainable EAF expansion and a stable national scrap supply system while reducing reliance on imported scrap and iron ore.

### Regional deployment and utilization of the electricity mix

**Figure 4:**
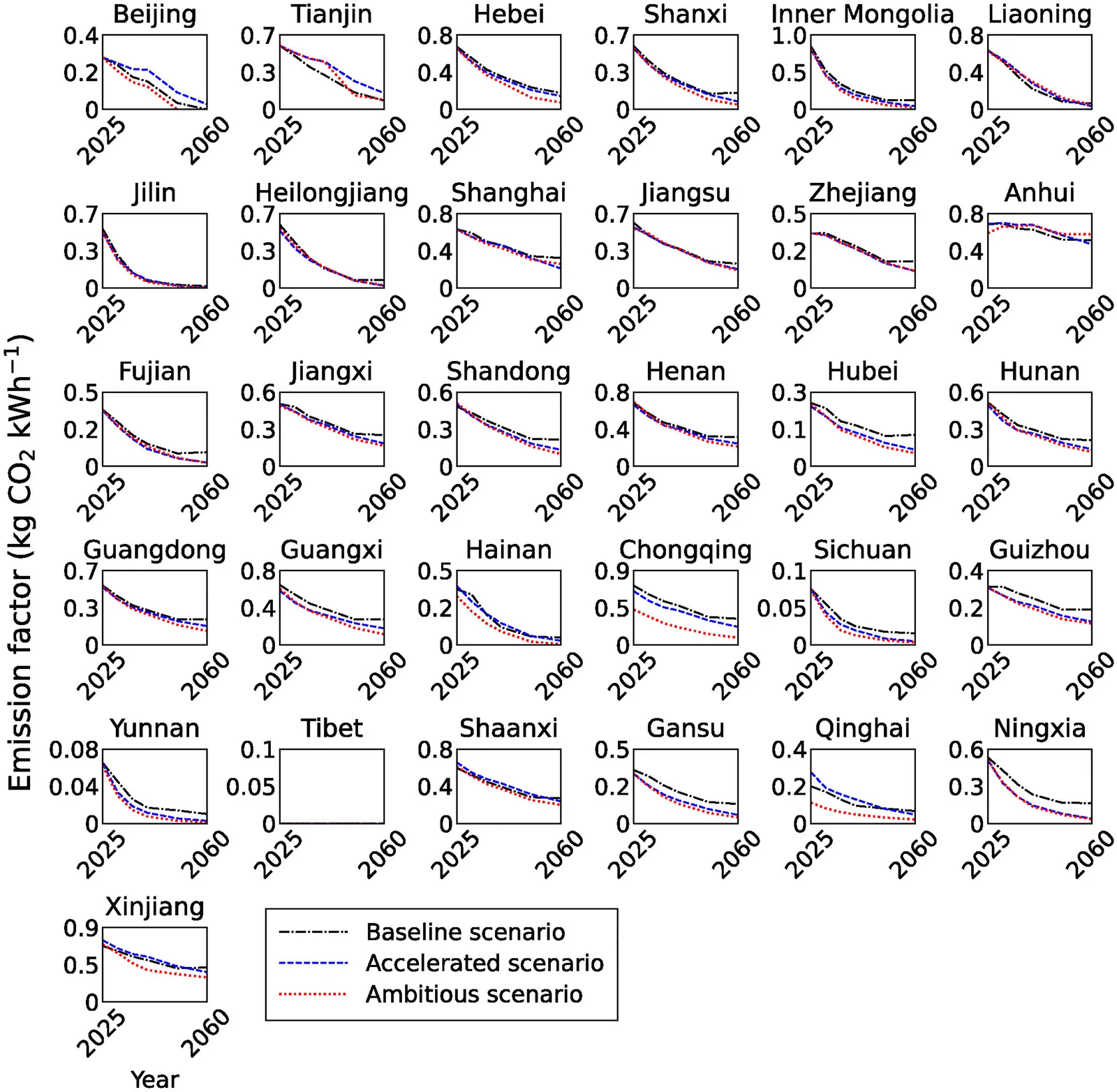
Carbon intensity trajectories of provincial power systems across scenarios Each image represents one of China’s 31 provinces. In every image, three curves illustrate the projected evolution of power-sector carbon emission factors under the baseline, accelerated, and ambitious scenarios.

The integration of green electricity can significantly reduce carbon emissions in the steel industry, making it necessary to systematically investigate the development pathways of provincial power systems. As shown in Figure 4, under the baseline, accelerated, and ambitious scenarios, the national-level carbon emission factors for electricity in 2060 are 0.171, 0.121, and 0.101 kg CO_2_ kwh^−1^, respectively. Overall, provincial power systems exhibit both commonalities and differences. The commonality lies in the continuous decline of carbon emission factors across provinces as the power system advances toward decarbonization. The differences reflect how provinces vary in their resource endowments, generation portfolios, and the temporal sequencing of decarbonization measures, such as the rate of renewable capacity deployment and fossil-fuel phase-out, which collectively result in heterogeneous emission-factor trajectories over time. For instance, provinces such as Hebei are characterized by a slowdown in emission reduction during the later stage of carbon neutrality; provinces such as Jiangsu maintain a consistently rapid decarbonization pace; while Anhui has not yet reached its emission peak.

In addition, under different scenarios, some provinces do not exhibit clear advantages. For example, the carbon emission factor of Beijing in the baseline scenario is lower than that in the accelerated scenario. Such cases are not unique to Beijing. They reflect the interplay of national-level constraints—provincial electricity deficits or surpluses, uneven availability of hydropower and wind resources, and the timing of capacity additions in neighboring provinces—which collectively determine how provinces compete for and rely on cross-regional electricity flows during the decarbonization process.

### Regional decarbonization pathways and policy options

**Figure 5:**
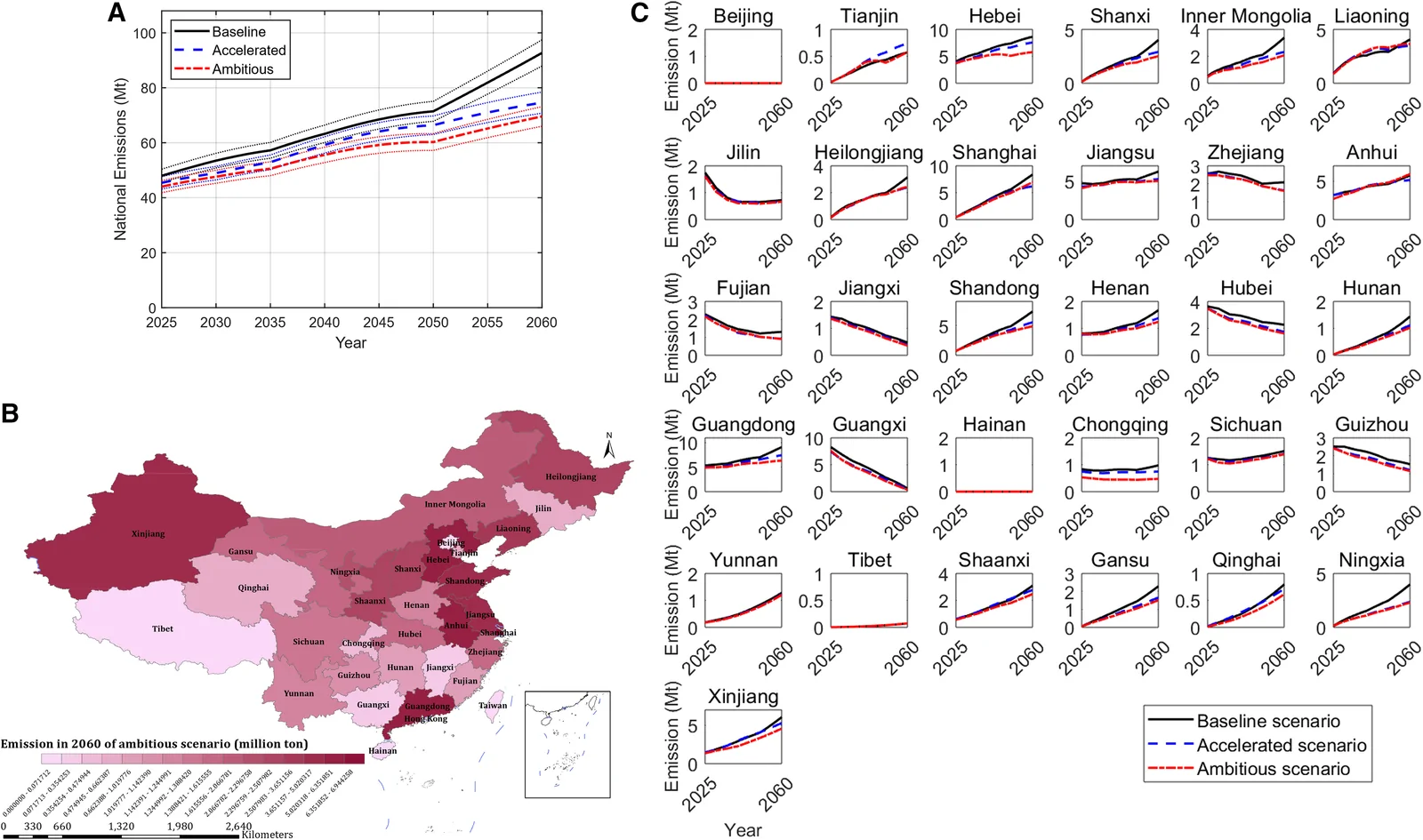
Provincial pathways for decarbonizing EAF steelmaking (A) National CO_2_ emissions from EAF steel production under the baseline, accelerated, and ambitious scenarios. (B) Provincial EAF-related CO_2_ emissions across the three scenarios, shown as comparative time-series trajectories. (C) Spatial distribution of provincial CO_2_ emissions from EAF steel production in 2060 under the ambitious scenario.

The decarbonization of China’s steel industry is not only a national priority but also a regional challenge, as provinces differ significantly in terms of steel production scale, scrap availability, electricity mix, and resource endowments. EAF steelmaking, as a key low-carbon pathway, exhibits diverse emission trajectories across provinces. Understanding these regional differences is crucial for designing effective and targeted policy interventions. Figure 5 presents the carbon emissions of EAF steelmaking in China under multiple scenarios. At the national level, total CO_2_ emissions from EAF steel production show an increasing trend across all three scenarios due to the continuous growth of EAF output. Electricity-related emissions dominate national CO_2_ emissions from EAF steel production, but their impact diminishes over time as cleaner electricity is deployed. In the baseline scenario, emissions reach 92.7 Mt in 2060. In the accelerated and ambitious scenarios, the growth is curbed, with emissions reduced to 74.6 and 69.6 Mt, respectively, demonstrating the potential of faster technology adoption and supportive policies.

At the provincial level, clear heterogeneity is observed. In most provinces, rising EAF production drives a steady increase in CO_2_ emissions. The total CO_2_ emissions under the three scenarios range from 69.6 to 92.7 Mt. Major EAF-producing provinces such as Guangdong and Jiangsu record the highest emissions. Although their unit CO_2_ intensity decreases, the large expansion of production leads to overall growth in emissions across all scenarios. By contrast, Beijing and Hainan are projected to have no EAF production capacity in the future due to policy orientation and historical development, resulting in zero EAF-related emissions. Meanwhile, several provinces show a declining emissions trend, such as Jilin, Zhejiang, Fujian, Jiangxi, Hubei, Guangxi, and Guizhou. These declines stem either from falling EAF output (e.g., Jiangxi and Guangxi), a sharp decrease in the electricity emission factor (e.g., Jilin), or the combined effect of relatively stable production and declining electricity intensity (e.g., Hubei and Guizhou). These findings indicate that decarbonization will proceed along differentiated regional pathways. Focusing on 2060 under the ambitious scenario, emissions are concentrated in southeastern coastal provinces such as Shandong, Jiangsu, and Shanghai, as well as in Anhui, Hebei, Guangdong, and Xinjiang. This is primarily driven by the scale of EAF output in these regions. Given these spatial patterns, effective mitigation requires targeted regional measures. In particular, ensuring a stable scrap supply and accelerating the decarbonization of the power mix in provinces such as Shanghai and Xinjiang should be considered policy priorities.

Using a province-level MFA-capacity integration framework, this study quantitatively links historical data, scenario-based projections of EAF steel output, dynamic scrap availability, and provincial electricity decarbonization to assess regional carbon emissions. The analysis reveals that carbon emissions are shaped by the interaction of three key factors: (1) local scrap supply, which constrains or enables EAF production; (2) electricity mix and its evolving carbon intensity, which determine the emissions associated with power consumption; and (3) regional production dynamics, including the scale and timing of EAF deployment relative to scrap generation. These interacting mechanisms produce pronounced spatial heterogeneity, with some provinces experiencing high emissions due to limited scrap or carbon-intensive power, while others achieve low emissions thanks to abundant scrap and cleaner electricity. By explicitly capturing these interactions, the framework highlights the provinces where targeted interventions, such as enhancing scrap circulation, prioritizing renewable integration, or supporting technology deployment, can achieve the largest decarbonization benefits. This province-specific, mechanism-based insight constitutes a key contribution of the study, providing a transferable approach for aligning material flows, industrial siting, and energy transitions in other steel-producing economies.

## Discussion

In this study, we present a comprehensive analysis of how provincial resource and energy availability shapes the development and decarbonization pathways of EAF steelmaking in China. Our findings indicate that both material and energy resources strongly determine regional EAF deployment, highlighting the importance of resource-informed planning for low-carbon steelmaking. First, provinces with abundant scrap resources show the highest potential for EAF expansion, provided that the distribution and quality of scrap are effectively managed. However, a persistent spatial mismatch exists between scrap availability and EAF capacity: while eastern and central provinces host the majority of EAF equipment, the largest scrap resources are often located in traditional BF-BOF regions. Our analysis indicates that this mismatch will be most pronounced in the near-term phase of EAF expansion, gradually easing in later years as scrap circulation and regional logistics systems develop. Although iron ore and coal circulate through well-established national and global markets, scrap is locally generated, unevenly recovered, and limited by quality-control and regulatory barriers that reduce long-distance mobility. These characteristics make scrap far more spatially constrained, necessitating provincial-level mismatch analysis. Given the limited mobility of existing BF-BOF capacity, regional scrap balances will shape EAF deployment in the medium term rather than prompt rapid plant relocation. Second, access to clean electricity is critical for low-carbon EAF operation. Provinces such as Sichuan, Yunnan, and Inner Mongolia have abundant renewable electricity, but these regions are often spatially separated from the main EAF production centers. Achieving low-carbon production therefore requires integrating EAF deployment with regional electricity supply, renewable energy availability, and electricity infrastructure planning.

Our findings on the dynamic scrap supply-demand patterns highlight that regional imbalances will evolve over time rather than remain static. This suggests that scrap circulation should be treated as a staged and adaptive process rather than a one-time allocation. In the early phase of EAF rollout, northern and eastern provinces are better positioned to support regions with deficits, whereas in the later phase, emerging surpluses in the south and southwest will become increasingly important. Such dynamics underscore the necessity of designing a flexible national scrap circulation system that can adapt to shifting regional balances. Beyond logistics, differentiated provincial policies are also essential: surplus regions should focus on improving collection and recycling efficiency, deficit regions on developing dismantling capacity and external sourcing channels, and transitional provinces in managing the timing of their shift from exporter to importer. By establishing this adaptive and regionally coordinated framework, China can reduce reliance on imported scrap and iron ore while ensuring a stable foundation for long-term EAF expansion.

At the national level, power-sector development is highly dependent on policy orientation. For example, recent reports on the construction of five new hydropower stations in Tibet illustrate how national planning can directly influence the carbon emission factor of the power system, leading to significant variations across different scenarios. At the provincial level, however, power development is shaped by a combination of factors, including regional resource endowments, policy environment, power flows, and interregional competition. Furthermore, the developmental pathways of provinces are not only determined by resource conditions but are also closely linked to their historical power structures and dependence on fossil fuels. As a major electricity consumer, the decarbonization potential of the steel industry is largely contingent upon the pace of low-carbon transition in the provincial power system.

The observed spatial mismatches between EAF production capacity, scrap availability, and clean electricity supply underscore the need for regionally differentiated policy design. Based on the results, provinces can be broadly categorized into three transition groups with distinct policy priorities. First, traditional steelmaking bases such as Hebei and Shanxi, which possess large existing BF-BOF capacities but face constraints in scrap availability and low-carbon electricity supply, should prioritize the gradual retirement or retrofitting of carbon-intensive assets while strengthening access to external scrap resources and renewable electricity. For Hebei, the top steel-producing province in China, local authorities can launch cross-regional scrap procurement partnerships with northern scrap-surplus provinces, and sign long-term green power subscription agreements to gradually replace coal-fired power for steel production. For Shanxi, policy priorities include phasing out outdated production lines alongside the construction of centralized scrap processing bases to offset local scrap shortages. Second, renewable-rich provinces such as Inner Mongolia, Yunnan, and Sichuan are well positioned to host new low-carbon EAF capacity and should be supported through green electricity priority arrangements, industrial clustering policies, and grid infrastructure investments. Sichuan and Yunnan, rich in hydropower resources, can roll out special industrial zones exclusively for low-carbon EAF projects and grant tax incentives to attract steel enterprises. Inner Mongolia can optimize its power grid connection capacity to transmit surplus wind and solar power to nearby EAF hubs in North China. Third, scrap-surplus regions with strong manufacturing and recycling bases, such as Jiangsu and Guangdong, should focus on establishing high-standard scrap collection, sorting, and quality certification systems to improve scrap utilization efficiency and support interregional resource allocation. Jiangsu and Guangdong can build provincial-level unified scrap trading platforms, formulate unified local standards for scrap classification, and subsidize interprovincial scrap logistics to lower circulation costs. More specifically, a national scrap quality certification and recycling standard system could be established to improve market transparency and facilitate long-distance scrap circulation. Cross-provincial coordination mechanisms, including dedicated scrap trading platforms and logistics corridors, could help balance regional supply-demand mismatches. In addition, green electricity priority arrangements, such as renewable power purchase agreements, green electricity certificates, and preferential grid access for low-carbon steel projects, could accelerate the integration of clean electricity into EAF production.

The emissions trajectories further reinforce the conclusion that regional decarbonization of EAF steelmaking will follow highly differentiated pathways. While unit CO_2_ intensity declines nationwide with cleaner electricity and improved technologies, overall emissions still rise in many provinces due to rapid production growth. Conversely, a subset of provinces exhibit declining emissions, either because of reduced EAF output or accelerated decarbonization of the power mix. This heterogeneity indicates that provincial contributions to national mitigation will not be uniform. Effective policy design must therefore move beyond aggregate targets and adopt regionally tailored approaches. Provinces with large and growing EAF output should prioritize securing low-carbon electricity supply and enhancing scrap allocation efficiency, whereas provinces with declining emissions can play a supportive role by exporting renewable electricity or surplus scrap to other regions. Transitional provinces with moderate growth should focus on coordinated planning to balance production expansion with electricity decarbonization. By aligning provincial pathways with national goals, China can turn regional disparities from potential obstacles into complementary strengths, ensuring that cumulative emissions reductions are maximized across the entire steel sector.

The modeling framework developed in this study is primarily tailored to China’s institutional and structural characteristics, where steel production, scrap generation, and electricity systems are organized at the provincial level under relatively constrained interregional flows. In particular, China exhibits pronounced spatial fragmentation in scrap availability, strong provincial heterogeneity in power-sector decarbonization, and relatively rigid cross-provincial industrial allocation, which jointly motivate a province-resolved analysis framework. Nevertheless, the core mechanisms identified in this study, including spatial mismatch between scrap supply and steel production, temporal misalignment between capacity expansion and resource availability, and the coupling between industrial electrification and power system transition, are broadly applicable to other major steel-producing economies. However, adapting the framework to regions such as the EU, the United States, or Japan would require adjustments in system boundaries and modeling assumptions, including the treatment of cross-border scrap trade, the representation of inter-regional electricity markets, and the characterization of industrial plant distribution and lifetime structures. Therefore, while the quantitative results are China-specific, the analytical structure and identified interaction mechanisms provide transferable insights for evaluating EAF transition pathways in other heterogeneous energy-industrial systems. Many steel-intensive economies worldwide are now promoting EAF transformation to cut industrial carbon emissions, and they encounter similar resource and spatial dilemmas. The European Union, with a mature scrap recycling system, also suffers from uneven scrap distribution and mismatches between renewable energy sites and steel production bases; member states such as Germany and Italy have started to build cross-border scrap logistics networks and joint green power sharing mechanisms to tackle this issue. Japan, relying heavily on imported resources, has formulated strict nationwide scrap quality standards and optimized domestic material circulation to support EAF development. Unlike these economies with relatively small territorial scales, the United States faces distinct challenges from vast land areas and fragmented regional energy policies, leading to prominent gaps between scrap supply, clean power and EAF capacity. Countries such as the EU members, the United States, and Japan face similar spatial gaps between the locations where scrap and electricity are generated and those where EAF capacity is located, shaped by differences in product lifetimes, industrial geography, and electricity decarbonization pace. The dynamic MFA-capacity integration framework developed here offers a transferable tool for addressing these challenges, demonstrating how coordinated planning of material flows, industrial siting, and power system transition across provinces, regions, and member states can support low-carbon EAF expansion across diverse contexts. For China, this broader insight underscores the need for strong national coordination to manage its pronounced regional disparities. Establishing a flexible scrap-circulation system and aligning electricity-infrastructure development with industrial transformation will be essential to prevent local resource constraints from impeding sector-wide decarbonization. Through such nationally integrated yet regionally tailored strategies, China can turn its uneven resource distribution into an advantage, building a more balanced and resilient pathway toward large-scale, low-carbon EAF steelmaking.

Overall, this study conducts a comprehensive province-level assessment of EAF steelmaking expansion in China, integrating MFA, capacity evaluation, and scenario modeling to link scrap supply-demand dynamics, electricity availability, and regional decarbonization potentials. The results confirm that while national scrap supply is generally sufficient to support future EAF expansion, significant spatial mismatches exist between scrap availability, EAF capacity distribution, and low-carbon electricity access. Provincial CO_2_ emissions from EAF steelmaking exhibit distinct patterns, driven by differences in production scales, electricity mixes, and scrap supply-demand balances across regions. To align regional EAF development with China’s steel decarbonization goals, coordinated national strategies tailored to provincial characteristics are essential. Key policy priorities include establishing dynamic interprovincial scrap circulation systems to address spatial mismatches, integrating EAF deployment with renewable-rich power resources, and implementing differentiated provincial pathways based on local resource endowments and industrial structures. This study provides a spatially resolved framework and actionable insights for balancing regional disparities, ensuring the sustainable and equitable expansion of EAF steelmaking as a core pathway toward China’s carbon neutrality targets.

### Limitations of the study

Several limitations of this study should be acknowledged. First, the analysis relies on exogenous socioeconomic drivers (e.g., GDP and population projections), which may not fully capture feedback effects between economic development and steel demand. Second, international scrap trade is not explicitly considered, which may affect regional scrap balance under future globalization and policy changes. Third, the electricity system is represented at an aggregated provincial level, without accounting for intra-provincial transmission constraints or spatial variability in renewable energy integration. Finally, plant-level heterogeneity within provinces is not captured, and the results therefore reflect average provincial conditions rather than firm-level differences. These limitations suggest directions for future research toward multi-scale and more granular modeling frameworks.

Future research can further extend this study in several directions. First, the integration of emerging low-carbon steelmaking technologies, such as H_2_-DRI and carbon capture, utilization, and storage (CCUS), would provide a more comprehensive representation of deep decarbonization pathways. Second, future work could downscale the analysis at the plant level to better capture intra-provincial heterogeneity in technology adoption and operational efficiency. Third, incorporating an explicit cost-benefit assessment of inter-provincial scrap and electricity transmission corridors would help evaluate the economic feasibility of coordinated resource allocation strategies. These extensions would further improve the robustness and policy relevance of the modeling framework.

## Resource availability

### Lead contact

Requests for further information and resources should be directed to and will be fulfilled by the lead contact, Qi Zhang (zhangqi@mail.neu.edu.cn).

### Materials availability

This study did not generate new materials. All relevant supporting information is provided in the supplemental files.

### Data and code availability

• All data used in this study are from publicly available sources, which are fully documented in the main text and supplemental information.
• This paper does not report original code.
• Any additional information required to reanalyze the data reported in this paper is available from the lead contact upon request.

## Acknowledgments

This work was supported by 10.13039/501100018537National Science and Technology Major Project (2025ZD1602503). The authors gratefully acknowledge the reviewers and editors for their fruitful comments.

## Author contributions

J.S., S.T., and Z.Z. conceptualized the study with support; performed the methodology design; carried out data curation, formal analysis, investigation, and visualization; and prepared the original draft. Z.L., H.L., P.W., and L.S. provided critical review, interpretation, and editing of the manuscript. L.W. provided supervision and contributed to manuscript review and editing. S.X. contributed to investigation and manuscript review and editing. Q.Z. led conceptualization and resources, supervised the project, guided manuscript revision, and acquired funding.s

## Declaration of interests

The authors declare no competing interests.

## STAR★Methods

### Key resources table

**Table:** 

REAGENT or RESOURCE	SOURCE	IDENTIFIER
**Softwareandalgorithms**		

MATLAB R2023b	The MathWorks, Inc.	https://www.mathworks.com

### Method details

#### Capacity and production of electric arc furnace

EAF steelmaking has emerged as a key decarbonization pathway for the steel industry. For carbon neutrality, understanding the future development of EAF production—particularly at the provincial level—is critical for supporting region-specific policy design, infrastructure planning, and resource allocation.^52^ To quantify the development potential of EAF steelmaking across provinces, a comprehensive evaluation framework was established based on five dimensions, namely resource supply, energy supply, economic competitiveness, market demand, and policy support (Table S1). These dimensions were represented by 20 secondary indicators, including scrap supply, electricity supply capacity, scrap price, crude steel consumption, and green transition policies. All indicators were first normalized according to their positive or negative effects. Subsequently, a combined weighting approach integrating the Analytic Hierarchy Process (AHP) and Entropy Weight Method (EWM) was adopted to balance subjective expert judgment and objective data variation. Specifically, AHP was used to determine subjective weights, while EWM was applied to derive objective weights from provincial data distributions. The final indicator weights were then incorporated into a TOPSIS model to calculate the relative closeness coefficient of each province to the ideal solution, which was defined as the provincial EAF development potential score. The EAF development potential score is constructed in Equations 1, 2, 3, 4, 5, and 6.

(Equation 1) $$x_{pj}^{\prime }=\frac{x_{pj}-\min\left(x_{j}\right)}{\max\left(x_{j}\right)-\min\left(x_{j}\right)}$$

(Equation 2) $$x_{pj}^{\prime }=\frac{\max\left(x_{j}\right){-x}_{pj}}{\max\left(x_{j}\right)-\min\left(x_{j}\right)}$$

(Equation 3) $$w_{j}=\frac{w_{j}^{AHP}*w_{j}^{EWM}}{\sum_{j=1}^{m}\left(w_{j}^{AHP}*w_{j}^{EWM}\right)}$$

(Equation 4) $$D_{p}^{+}=\sqrt{\sum_{j=1}^{m}{\left\langle w_{j}*x_{pj}^{\prime }-\left\{\max\left(w_{j}*x_{pj}^{\prime }\right)\right\}\right\rangle }^{2}}$$

(Equation 5) $$D_{p}^{-}=\sqrt{\sum_{j=1}^{m}{\left\langle w_{j}*x_{pj}^{\prime }-\left\{\min\left(w_{j}*x_{pj}^{\prime }\right)\right\}\right\rangle }^{2}}$$

(Equation 6) $$S_{p}=\frac{D_{p}^{-}}{D_{p}^{+}+D_{p}^{-}}$$

where $x_{pj}^{\prime }$ denotes normalized value of indicator *j* for province *p*; *x*_*pj*_ denotes original value of indicator *j* for province *p*; *w*_*j*_ denotes the final combined weight of indicator *j*; $w_{j}^{AHP}$ denotes the subjective weight of indicator *j* determined by AHP; $w_{j}^{EWM}$ denotes the subjective weight of indicator *j* determined by EWM; $D_{p}^{+}$ denotes the Euclidean distance between province *p* and the positive ideal solution; $D_{p}^{-}$ denotes the Euclidean distance between province *p* and the negative ideal solution; *S*_*p*_ denotes the score of EAF development potential in province p.

This study constructs a province-level MFA-capacity integration framework to estimate the capacity and production of EAF steelmaking in China from 2025 to 2060. The analysis covers all 31 provincial-level administrative regions in mainland China, excluding Hong Kong, Macao, and Taiwan, and integrates both historical data and forward-looking scenarios. The EAF capacities in different provinces are shown in Equations 7, 8, and 9.

(Equation 7) $$C_{p}\left(t\right)=C_{o,p}+\left(C_{2060\;\max,p}-C_{o,p}\right)*\frac{\left(1-e^{k_{p}t}\right)}{\left(1-e^{k_{p}T}\right)}$$

(Equation 8) kp=1Sp

(Equation 9) C2060max,p=Sp−Sp,minSp,max−Sp,min∑pSp−Sp,minSp,max−Sp,min∗C2060max,total

where *C*_*p*_(*t*) denotes the EAF capacity of year t in province p; *C*_*o*,*p*_ denotes the EAF capacity of base year in province p; *C*_2060*max*,*p*_ denotes the upper limit of EAF capacity in 2060 in province p; *k*_*p*_ denotes the change rate of EAF in province p; T denotes the research cycle; *C*_2060*max*,*total*_ is the total national capacity of EAF in 2060.

To capture the temporal evolution of EAF technologies, this study differentiates EAF types by their representative technological characteristics, such as energy efficiency, automation level, scrap preheating technologies, and low-carbon features. Five representative EAF technology generations (Gen 1–Gen 5) are considered, ranging from conventional EAFs with relatively high electricity consumption and limited automation to advanced low-carbon EAFs characterized by intelligent operation, enhanced energy efficiency, and the integration of emerging technologies such as hydrogen metallurgy and AI-assisted process optimization. Detailed technological characteristics, commissioning periods, electricity consumption levels, melting times, and equipment lifetimes for each EAF generation are summarized in Table S2. Among these differentiated technology profiles, the more advanced tiers represent forward-looking assumptions that capture expected trends in digitalization, automation, energy-efficiency improvements, and low-carbon process innovations. The definition and representation of key scenario parameters are shown in Table S3. The modeling of EAF capacity evolution incorporates three key considerations. First, different technology profiles are assumed to have distinct peak deployment periods. For example, intermediate-level profiles are expected to experience accelerated adoption around 2030, whereas the most advanced low-carbon profiles are assumed to expand gradually after 2050. To reflect regional heterogeneity in deployment timing, a normal distribution function is applied to simulate the annual share of each technology profile, capturing the probability distribution of adoption over time, as shown in Equation 10. The scenario-specific assumptions regarding commissioning years and construction peak widths for different EAF generations are provided in Table S4. Second, EAF equipment gradually retires rather than operating indefinitely. To account for lifespan-related capacity dynamics, a Weibull distribution function is used to represent the retirement pattern of each technology profile, as shown in Equations 11, 12, and 13. This enables the estimation of in-service capacity for each profile in a given year and provides a more realistic representation of cumulative retirement and renewal dynamics. The corresponding assumptions on equipment lifetime and Weibull shape parameters are also summarized in Table S4. Third, the proportion of in-service equipment in each profile is used to allocate total EAF capacity across the differentiated technological tiers, as shown in Equation 14. This approach enables a dynamic representation of the evolving capacity mix and forms the basis for subsequent energy demand and emission calculations.

(Equation 10) $$I_{p,g}\left(t\right)=\frac{\exp\left(-\frac{1}{2}{\left(\frac{t-\mu_{g}^{p}}{\sigma_{g}^{p}}\right)}^{2}\right)}{\sum_{g^{\prime }=1}^{G}\;\exp\left(-\frac{1}{2}{\left(\frac{t-\mu_{g^{\prime }}^{p}}{\sigma_{g^{\prime }}^{p}}\right)}^{2}\right)}$$

(Equation 11) $$A_{p,g}\left(t\right)=\sum_{t^{\prime }=t0}^{t}I_{p,g}\left(t^{\prime }\right)*S_{p,g}\left(t-t^{\prime }\right)$$

(Equation 12) $$S_{p,g}\left(t-t^{\prime }\right)=\exp\left(-{\left(\frac{t-t^{\prime }}{\eta_{g}}\right)}^{\beta_{p,g}}\right)$$

(Equation 13) $$\alpha_{p,g}\left(t\right)=\frac{A_{p,g}\left(t\right)}{\sum_{g^{\prime }=1}^{G}A_{p,g^{\prime }}\left(t\right)}$$

(Equation 14) $$C_{p,g}\left(t\right)=\alpha_{p,g}\left(t\right)*C_{p}\left(t\right)$$

where *I*_*p*,*g*_(*t*) denotes the share of newly added EAFs of type *g* in province *p* in year *t*; $\mu_{g}^{p}$ represents the main commissioning year of type *g* in province *p*; $\sigma_{g}^{p}$ indicates the standard deviation reflecting the peak width of construction for type *g* in province *p*; *g* denotes the total number of EAF types, with *g* = 5; *A*_*p*,*g*_(*t*) denotes the proportion of in-service equipment of type *g* in province *p* in year *t*; *t*-*t*′ denotes the service age of the equipment; *S*_*p*,*g*_(*t*-*t*′) denotes the Weibull distribution function for type *g*; *η*_*g*_ denotes the average service life of type *g*; *β*_*p*,*g*_ denotes the Weibull shape parameter for type *g* in province *p*; *α*_*p*,*g*_(*t*) denotes the normalized value of the in-service proportion of type *g* in province *p* in year *t*; *C*_*p*,*g*_(*t*) denotes the EAF capacity of type *g* in province *p* in year *t*.

To estimate the actual steel production, the steelmaking capacity is adjusted using the capacity utilization rate. Accordingly, the production of EAFs is determined by Equation 15.

(Equation 15) $$P_{p,g}\left(t\right){=C}_{p,g}\left(t\right)*R_{p,g}\left(t\right)$$

where *P*_*p*,*g*_(*t*) denotes the EAF production of type *g* in province *p* in year *t*; *R*_*p*,*g*_(*t*) denotes the capacity utilization rate of EAFs of type *g* in province *p* in year *t*, which is determined by the product of two factors: *f*_*g*_, the national baseline adjustment factor for EAF type *g*, and *f*_*p*_, the regional adjustment factor of province *p*.

#### Demand and supply of steel scrap

Given that EAF steel production is highly dependent on steel scrap as the primary raw material, it is essential to assess the scrap demand and supply to ensure resource availability and system balance. The following analysis quantifies both the scrap demand driven by EAF production and the potential supply from domestic sources. The steel scrap demand is determined by the scale of EAF steel production, as formulated in Equation 16.

(Equation 16) $${SD}_{p}\left(t\right)=\sum_{g}\left[P_{p,g}\left(t\right)*Q_{g}\right]$$

where *SD*_*p*_(*t*) denotes the steel scrap demand in province *p* in year *t*; *Q*_*g*_ denotes the steel scrap demand per ton of steel production for type *g*.

The supply of steel scrap includes three main sources: home scrap, prompt scrap, and EOL scrap from five major end-use sectors—building, machinery, power, appliance, and automobile.^41^^,^^55^^,^^56^ At the provincial level, this study considers the following: Home scrap refers to the internal waste generated during the steelmaking process within steel plants in each province. Its volume is determined by the province’s crude steel production, as shown in Equation 17. Prompt scrap refers to the waste generated during the processing of steel products in local fabrication plants, such as cutting, stamping, and machining operations during the manufacturing of metal parts, components, and finished goods. Its volume is estimated based on the apparent steel consumption in each province, as shown in Equation 18.

(Equation 17) $${HS}_{p}\left(t\right)=k_{s}\left(t\right)*P_{p,g}\left(t\right)$$

(Equation 18) $${PS}_{p}\left(t\right)=k_{n}\left(t\right)*{AS}_{p,g}\left(t\right)$$

where *HS*_*p*_(*t*) denotes the amount of home scrap resources in province *p* in year *t*; *k*_*s*_(*t*) and *k*_*n*_(*t*) denote the recovery rates of home scrap and prompt scrap in year *t* respectively, with baseline values set at 7.5% and 6% and varying over time according to the scenario assumptions; *PS*_*p*_(*t*) denotes the amount of prompt scrap resources in province *p* in year *t*; *AS*_*p*,*g*_(*t*) denotes the apparent steel consumption in province *p* in year *t*.

The generation of EOL scrap is closely related to the size and dynamics of in-use steel stock across different sectors, as EOL steel becomes available for recycling once products or infrastructure reach the end of their service life. This stock is influenced by sector-specific drivers: for example, steel stock in the building and machinery sectors is primarily driven by floor area and population; in the automobile and appliance sectors, it is influenced by population and GDP; and in the power sector, it is linked to installed capacity by power type and the transmission distance of various grid lines. The detailed calculation based on stock-driven dynamic material flow analysis is shown in Equations 19, 20, and 21.

(Equation 19) SPTpi=1(t)=∑b[PTp,bi=1(t)∗CCbi=1]PPp(t)

where *i* denotes the end-use sectors, such as building, machinery, power, appliance, and automobile; ${SPT}_{p}^{i=1}\left(t\right)$ denotes the per capita in-use steel stock of building sector in province *p* in year *t*; *b* denotes the types of steel products in building sector, such as residential building, public building, and industrial building; ${PT}_{p,b}^{i=1}\left(t\right)$ denotes the product in-use stock of building sector in province *p* in year *t*; ${CC}_{b}^{i=1}$ denotes the steel consumption coefficient of different steel product types in building sector; *PP*_*p*_(*t*) denotes the population of province *p* in year *t*. Subsequently, a polynomial function is fitted to project its future values. Combined with future population projections, this yields the total steel stock in the building sector (${ST}_{p}^{i=1}\left(t\right)$). The in-use steel stock of the machinery sector, ${ST}_{p}^{i=2}\left(t\right)$, is estimated based on its relationship with the in-use steel stock of the building sector, using a sectoral conversion factor between machinery and building of 0.326.

(Equation 20) $${ST}_{p}^{i=3}\left(t\right)=\sum_{es}\left[{PT}_{p,es}^{i=3}\left(t\right)*{CC}_{es}^{i=3}\right]+\sum_{et}\left[{PT}_{p,et}^{i=3}\left(t\right)*{CC}_{et}^{i=3}\right]$$

where ${ST}_{p}^{i=3}\left(t\right)$ denotes the in-use steel stock of power sector in province *p* in year *t*; *es* denotes the types of steel products of power generation facility, such as hydropower, thermal power, nuclear power, wind power, solar photovoltaic, and others; *et* denotes the types of steel products of power transmission facility, such as above 330 kV, 330 kV, 220 kV, 110 kV, 35 kV, and cables. Similarly, future values are obtained by fitting historical data.

(Equation 21) SPTpi=4(t)=∑a[PTp,ai=4(t)∗CCai=4]PPp(t)

where ${SPT}_{p}^{i=4}\left(t\right)$ denotes the per capita in-use steel stock of appliance sector in province *p* in year *t*; *a* denotes the types of steel products in appliance sector, such as washing machine, refrigerator, microwave oven, television, air conditioner, and heater.

This study fits a Gompertz function to the historical relationship between per capita steel stock in the appliance sector and per capita GDP, as shown in Equation 22.

(Equation 22) $${SPT}_{p}^{i=4}\left(t\right)=H*e^{\left(-l*e^{\left(-k*{PG}_{t,p}\right)}\right)}$$

where *H* denotes the saturation level; *l* denotes the displacement coefficient; *k* is the growth rate coefficient; *PG*_*t*,*p*_ represents the per capita GDP in province *p* in year *t*. After estimating the parameters *H*, *l*, and *k* through curve fitting, future per capita steel stock values are projected. Combined with population projections, the total steel stock in the appliance sector (${ST}_{p}^{i=4}\left(t\right)$) is obtained. The total steel stock in the automobile sector (${ST}_{p}^{i=5}\left(t\right)$) is calculated in a similar manner. The only difference lies in the classification of automobile steel products, which include passenger vehicles, motorcycles, and electric-assisted bicycles.

By aggregating the in-use steel stock across the sectors, the amount of EOL scrap is calculated in Equation 23.

(Equation 23) $${ES}_{p}^{i}\left(t\right)={ST}_{p}^{i}\left(t\right)*{GF}^{i}\left(t\right)$$

where ${ES}_{p}^{i}\left(t\right)$ denotes the amount of EOL scrap resources of sector *i* in province *p* in year *t*; ${ST}_{p}^{i}\left(t\right)$ denotes the total in-use steel stock of sector *i* in province *p* in year *t*; *GF*^*i*^(*t*) denotes the steel scrapping rate of sector *i* in year *t*.

The recycling rates of scrap steel resources vary depending on the type and source of the scrap. Therefore, these differences should be analyzed separately. Home scrap and prompt scrap are defined as high-quality scrap due to minimal impurities during their generation. EOL scrap is categorized by industry, and the quantity of low-, medium-, and high-quality scrap for each sector is calculated using a scrap quality index (*Q*), whereas detailed in Equation 24. Following the principle of “high quality for high utilization, low quality for low utilization,” this study further models the actual recycled scrap volumes, as shown in Equations 25, 26, and 27.

(Equation 24) $$f_{i}\left(Q\right)=\frac{{WB}_{i}}{{WA}_{i}}{\left(\frac{Q}{{WA}_{i}}\right)}^{{{WB}_{i}}^{-1}}e^{-{\left(Q/{WA}_{i}\right)}^{{WB}_{i}}}$$

where *f*_*i*_(*Q*) denotes the probability density of scrap quality index *Q* in sector *i*; *WA*_*i*_ is the scale parameter of sector *i*; *WB*_*i*_ is the shape parameter of sector *i*.

(Equation 25) $${SU}_{h}\left(t\right)={[HS}_{p}\left(t\right)+{PS}_{p}\left(t\right)+\sum_{i}{ES}_{p}^{i}\left(t\right)*f_{i}\left(Q>8\right)]*{UR}_{h}\left(t\right)$$

(Equation 26) $${SU}_{m}\left(t\right)=\left[\sum_{i}{ES}_{p}^{i}\left(t\right)*f_{i}\left(2<Q<8\right)\right]*{UR}_{m}\left(t\right)$$

(Equation 27) $${SU}_{l}\left(t\right)=\left[\sum_{i}{ES}_{p}^{i}\left(t\right)*f_{i}\left(Q<2\right)\right]*{UR}_{l}\left(t\right)$$

where *SU*_*h*_(*t*), *SU*_*m*_(*t*), *SU*_*l*_(*t*) denote the recycled scrap volumes of high, medium, and low qualities; *UR*_*h*_(*t*), *UR*_*m*_(*t*), *UR*_*l*_(*t*) denote the recycling rates of high, medium, and low qualities.

#### Power generation mix and emission factor

This study adopts the method of literature and constructs provincial power development pathways under different scenarios with the objective of minimizing provincial indirect carbon emissions.^24^ The study also incorporates the recently constructed Yarlung Tsangpo Hydropower^57^ Station into the model, as shown in Equations 28, 29, and 30.

Objective function:

(Equation 28) $$\mathrm{Min}:C_{t}=\sum_{p_{out}}\left[{EF}_{p,out}\times \left(\sum_{j}G_{p_{out},j,t}-C_{p_{out},t}\right)\right]$$

where *EF* denotes the provincial carbon emission factor, *G* represents electricity generation, *p* refers to provinces, *j* to technologies, and t to years.

Carbon emission factor of electricity net-exporting provinces:

(Equation 29) $${EF}_{p_{out},t}=\frac{G_{p_{out},j_{gas},t}\times {ef}_{p_{out},j_{coal},t}+G_{p_{out},j_{coal},t}\times {ef}_{p_{out},j_{coal},t}}{\sum_{j}G_{p_{out},j,t}}$$

Carbon emission factor of electricity net-importing provinces:

(Equation 30) $${EF}_{p_{in},t}=\frac{G_{p_{in},j_{gas},t}\times {ef}_{p_{in},j_{coal},t}+G_{p_{in},j_{coal},t}\times {ef}_{p_{in},j_{coal},t}+\sum_{j}D_{p_{in}i_{out},t}\times {EF}_{p_{out},t}}{\sum_{j}G_{p_{out},j,t}+\sum_{j}D_{p_{in}p_{out},t}}$$

where *ef* represents the emission factor of each technology, and *D* indicates the inter-provincial transmission of electricity. Only natural gas and coal-fired power generation are considered. The term applies to electricity-exporting provinces and the other to electricity-importing provinces. *p*_*out*_ refers to electricity-exporting provinces, while *p*_*in*_ refers to electricity-importing provinces.

The model is primarily constrained by national power supply–demand balance, renewable energy generation limits, and renewable energy penetration rate limits, as shown in Equations 31, 32, 33, 34, 35, and 36.

National electricity supply–demand balance:

(Equation 31) $$\sum_{p}\sum_{j}G_{p,j,t}=\sum_{p}G_{p,t}$$

Renewable energy generation limit:

(Equation 32) $$\sum_{p}G_{p,j_{renew},t}\leq Potential_{j_{renew},t}$$

where $Potential_{j_{renew},t}$ denotes the renewable energy generation.

Renewable energy penetration rate constraint:

(Equation 33) $${PF}_{p,t}=\frac{G_{p,j_{coal},t}+G_{p,j_{gas},t}+G_{p,j_{biomass},t}}{\sum_{j}G_{p,j,t}}$$

(Equation 34) $${\mathrm{PR}}_{p,t}=1-{PF}_{p,t}\leq {1-PF}_{p,t-1}\times \left(1-n\right)$$

where *PF* and *PR* denote the penetration rates of renewable energy generation.

Energy security constraint:

(Equation 35) $$\frac{G_{p,j,t}-G_{p,j,t-1}}{G_{p,j,t-1}}\geq R_{j,t}$$

where *R*_*j*,*t*_ denotes the increment of electricity generation from technology *j* in year *t* compared with year *t*-1.

Policy constraint:

(Equation 36) $$\frac{G_{p,j,t}/h_{p,j}}{\sum_{j}\left(G_{p,j,t}/h_{p,j}\right)}\geq P_{p,j,t}$$

where *h* denotes the generation hours, and *P*_*p*,*j*,*t*_ represents the planned installed capacity.

#### Future CO_2_ emission trends of electric arc furnace route

CO_2_ emissions from steel production can be categorized into direct and indirect emissions.^58^ Direct emissions refer to CO_2_ emissions released during on-site fuel combustion processes, particularly from the consumption of fossil fuels such as coke and coal in EAF route. Indirect emissions, on the other hand, originate from upstream energy-related activities, primarily associated with electricity consumption, which accounts for a significant portion of the energy input in EAF route. The detailed calculation methods for both types of emissions are presented in Equations 37 and 38.

(Equation 37) $${DC}_{p,g}\left(t\right)=\sum_{f}{FC}_{g}^{f}*{FF}^{f}*P_{p,g}\left(t\right)$$

(Equation 38) $${IC}_{p,g}\left(t\right)={EC}_{g}*{EF}_{p,t}*P_{p,g}\left(t\right)$$

where *DC*_*p*,*g*_(*t*), *IC*_*p*,*g*_(*t*) denote the direct and indirect emissions of EAF production of type *g* in province *p* in year *t*; ${FC}_{g}^{f}$ denotes the net consumption of fossil fuel type *f* for EAF type *g*; *FF*^*f*^ denotes the emission factor of fuel type *f*; *EC*_*g*_ denotes the net electricity consumption of type *g*; *EF*_*p*,*t*_ denotes the emission factor of type *g* in province *p*.

#### Scenario setting and data

To explore how regional resource and energy availability influence the decarbonization pathways of EAF route in China, this study develops three differentiated scenarios: Baseline, Accelerated, and Ambitious. The three scenarios are designed to reflect different levels of policy support, technological progress, and implementation intensity for EAF development in China. The Baseline scenario assumes the continuation of current industrial and energy transition policies, with moderate growth in EAF capacity, gradual improvements in scrap recycling efficiency, and power-sector decarbonization following existing plans. The Accelerated scenario assumes enhanced policy incentives for low-carbon steelmaking, faster deployment of EAF technologies, improved scrap collection systems, and a more rapid decline in grid emission intensity. The Ambitious scenario represents a highly proactive transition pathway characterized by strong policy interventions, accelerated technological advancement, high scrap recovery efficiency, and an accelerated shift toward low-carbon electricity supply. The differences among the three scenarios are primarily reflected in the following dimensions. (1) Dynamic development of EAF capacity and production. This aspect is characterized by several key parameters: the main commissioning year of new EAF installations, the width of the construction peak period, the average service life of EAFs, the shape parameter of the Weibull distribution (for modeling EAF retirements), and the capacity utilization rate. (2) Relationship between scrap supply and demand. This dimension captures the changing patterns of scrap collection and utilization, primarily driven by improvements in the scrap resources recycling rate across scenarios. (3) Carbon emission trends. The carbon emission intensity of the power sector is a critical factor in determining indirect emissions from EAF production. Thus, the electricity emission factor is varied across scenarios to reflect differing levels of grid decarbonization. All scenario-specific parameters are detailed in Figures S1–S4 and Table S4 in the Supplementary Materials.

In addition, this study involves extensive data inputs for modeling and calculations. These include province-level socioeconomic parameters such as population and GDP (Tables S5 and S6 for historical data^59^ and Tables S7 and S8 for future projections^60^), historical in-use stocks of various steel-using products across five major sectors in each province (Table S9),^44^^,^^59^^,^^61^ steel consumption coefficients of different steel product types (Table S10),^17^ sector-specific steel scrapping rates (Figure S5), the time-series values of recovery rates (Figure S6), and the material and energy flows of scrap-EAF route by EAF type (Table S11).

To further assess the influence of key parameter uncertainties on the projected emission pathways, a sensitivity analysis is conducted as described below, as summarized in Table S12. These parameters include scrap recovery rate, EAF service life, Weibull shape parameter, capacity utilization rate, and scrap input–output ratio. Each parameter is perturbed individually while keeping others unchanged, and the model is re-run to evaluate the resulting changes in EAF production, scrap availability, and CO_2_ emission trajectories over time. In particular, the effects of parameter uncertainty are directly propagated into future emission pathways, allowing the construction of bounded CO_2_ emission ranges under different scenarios. This enables the derivation of uncertainty intervals for key indicators, including the future emission trends, thereby providing a clearer representation of how parameter assumptions influence long-term decarbonization outcomes.

### Quantification and statistical analysis

There are no quantification or statistical analyses to include in this study.
