# Supplementary material for: Tailoring electric arc furnace steelmaking expansion pathways based on regional scrap and energy availability in China
Source: iScience. 2026 Sep 12;29(10):117512. doi: 10.1016/j.isci.2026.117512 (PMC13586870; doi:10.1016/j.isci.2026.117512)
Supplement: Supplementary file 1 [file mmc1.pdf]

iScience, Volume *29*

Supplemental information

Tailoring electric arc furnace steelmaking expansion pathways based on regional scrap and energy availability in China

Jialin Shen, Shuoshuo Tian, Zhao Zhang, Zhaoling Li, Hongyou Lu, Peng Wang, Lu Sun, Linyang Wei, Sheng Xie, and Qi Zhang

Supplementary Material

Contents

Section 1. Methodology

Figure S1. The capacity utilization rates of EAF in different scenarios

Figure S2. The EOL scrap steel resource distribution by sector in the base year

Figure S3. The proportional distribution of EOL scrap steel resources by sector

Figure S4. The scrap resources recycling rate in different scenarios

Figure S5. The steel scrapping rates in different end-use sectors

Figure S6. The specific time-series values of recovery rates

Table S1. Assessment system for provincial EAF development potential scores

Table S2. The classification of EAF equipment types

Table S3. Definition and representation of key scenario parameters

Table S4. The parameters of the capacity modeling of the five types of EAF steelmaking in different scenarios

Table S5. The province-level historical population

Table S6. The province-level historical per capita GDP

Table S7. The province-level future population

Table S8. The province-level future GDP

Table S9. The historical in-use stocks of various steel-using products by sector

Table S10. The steel consumption coefficients of different steel products in different end-use sectors

Table S11. The material and energy flows of scrap- EAF route by EAF type

Table S12. Parameter settings and variation ranges used in the sensitivity analysis

Section 2. Results

Figure S7. The EAF capacity trends of China by type in baseline scenario

Figure S8. The EAF capacity trends of China by type in accelerated scenario

Figure S9. The EAF capacity trends of China by type in ambitious scenario

Figure S10. The EAF production trends of China by type in baseline scenario

Figure S11. The EAF production trends of China by type in accelerated scenario

Figure S12. The EAF production trends of China by type in ambitious scenario

Figure S13. The EOL scrap steel generated by building industry in each province

Figure S14. The EOL scrap steel generated by machine industry in each province

Figure S15. The EOL scrap steel generated by power infrastructure in each province

Figure S16. The EOL scrap steel generated by power transformation in each province

Figure S17. The EOL scrap steel generated by appliance industry in each province

Figure S18. The EOL scrap steel generated by automotive industry in each province

Figure S19. The total EOL scrap steel in each province

Figure S20. The home scrap steel in each province

Figure S21. The prompt scrap steel in each province

Figure S22. The total scrap steel in each province

Figure S23. The recycled scrap in each province in baseline scenario

Figure S24. The recycled scrap in each province in accelerated scenario

Figure S25. The recycled scrap in each province in ambitious scenario

Figure S26. The power demand of EAF in each province in baseline scenario

Figure S27. The power demand of EAF in each province in accelerated scenario

Figure S28. The power demand of EAF in each province in ambitious scenario

Figure S29. Provincial power generation structure from 2025 to 2060

Figure S30. The emissions generated by EAF steelmaking by province

Figure S31. The simulated results from 2013 to 2022

Section 1. Methodology

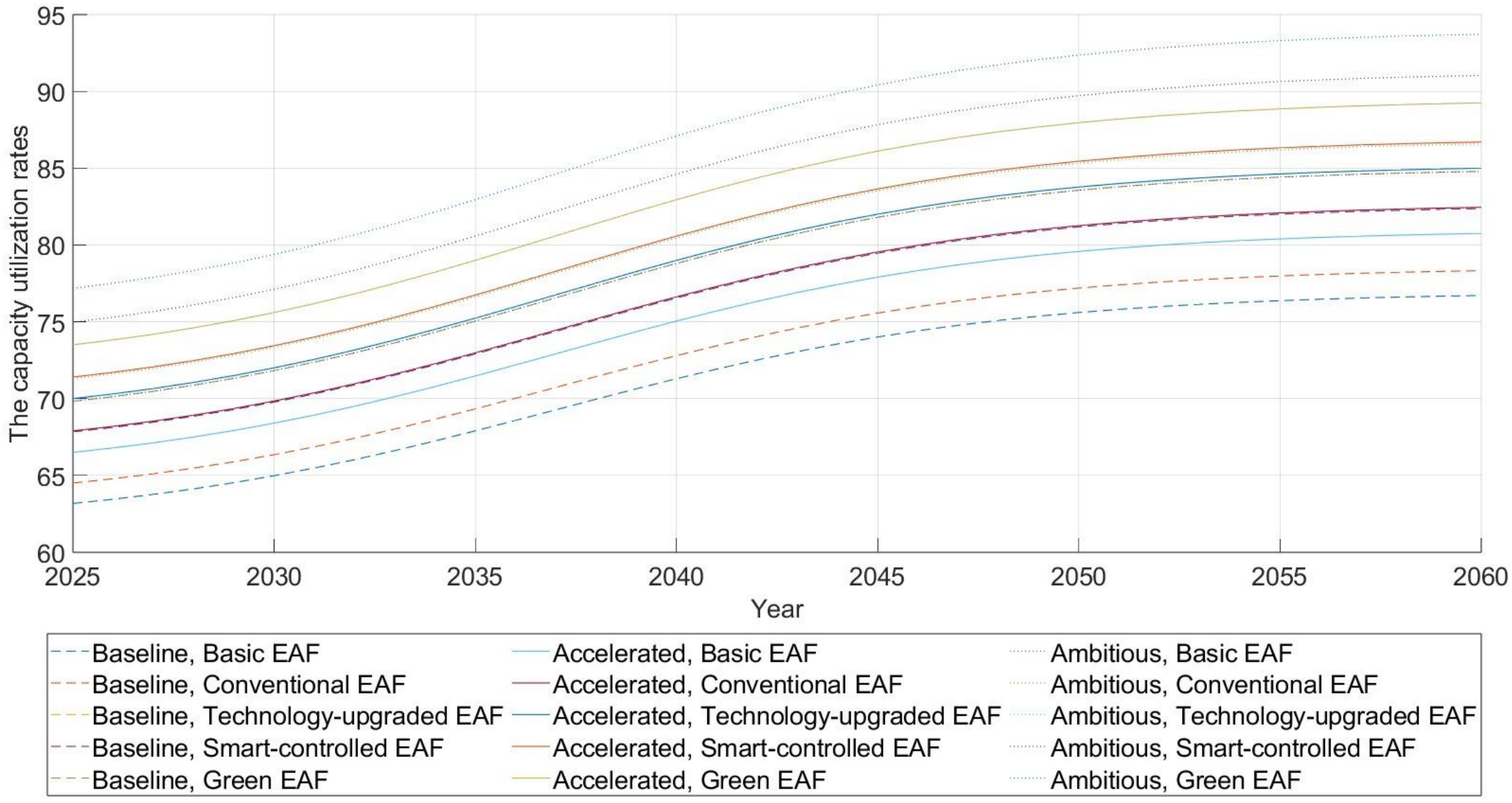

Figure S1. The capacity utilization rates of EAF in different scenarios

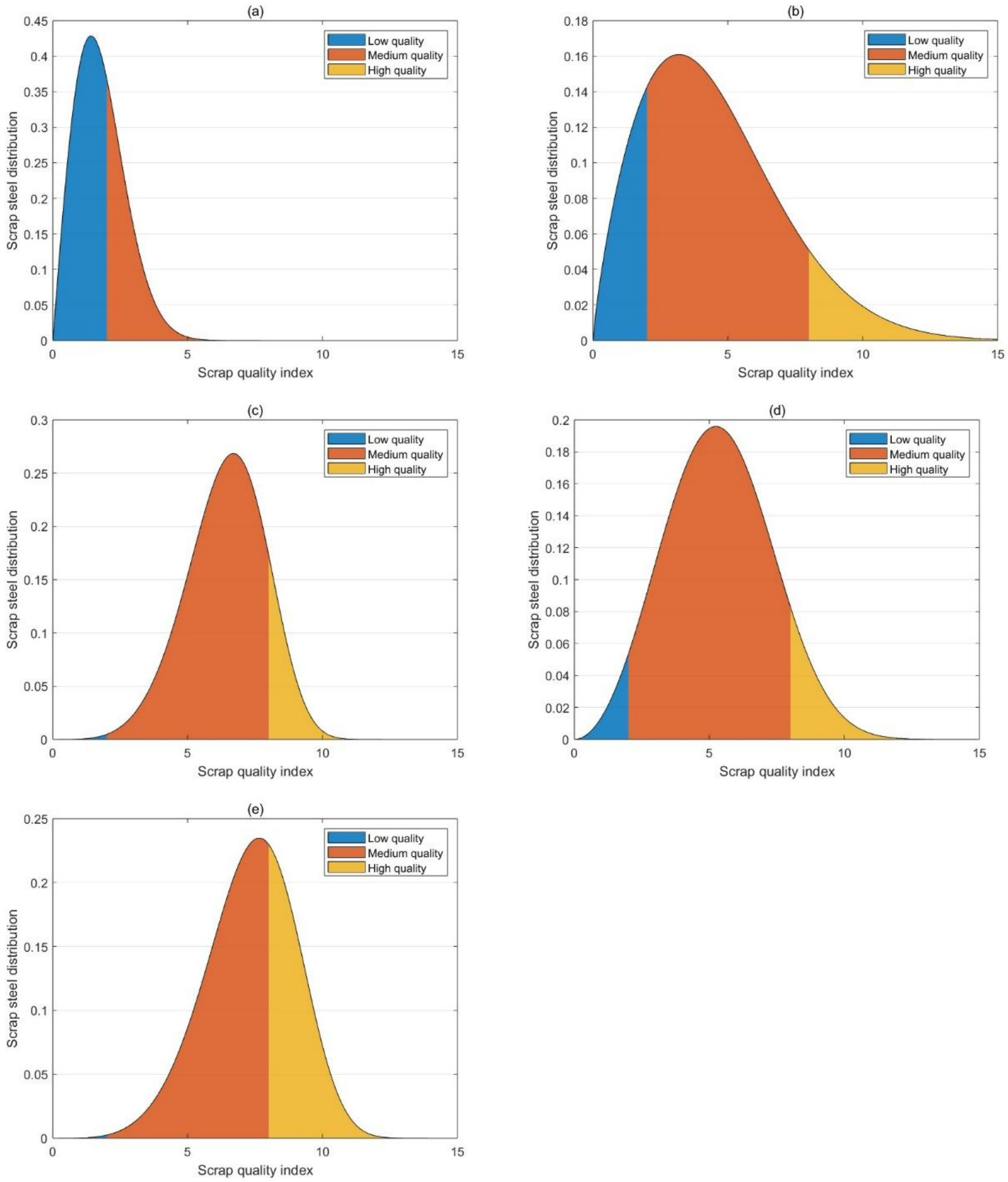

Figure S2. The EOL scrap steel resource distribution by sector in the base year. (a) Building; (b) Machinery; (c) Power; (d) Appliance; (e) Automotive

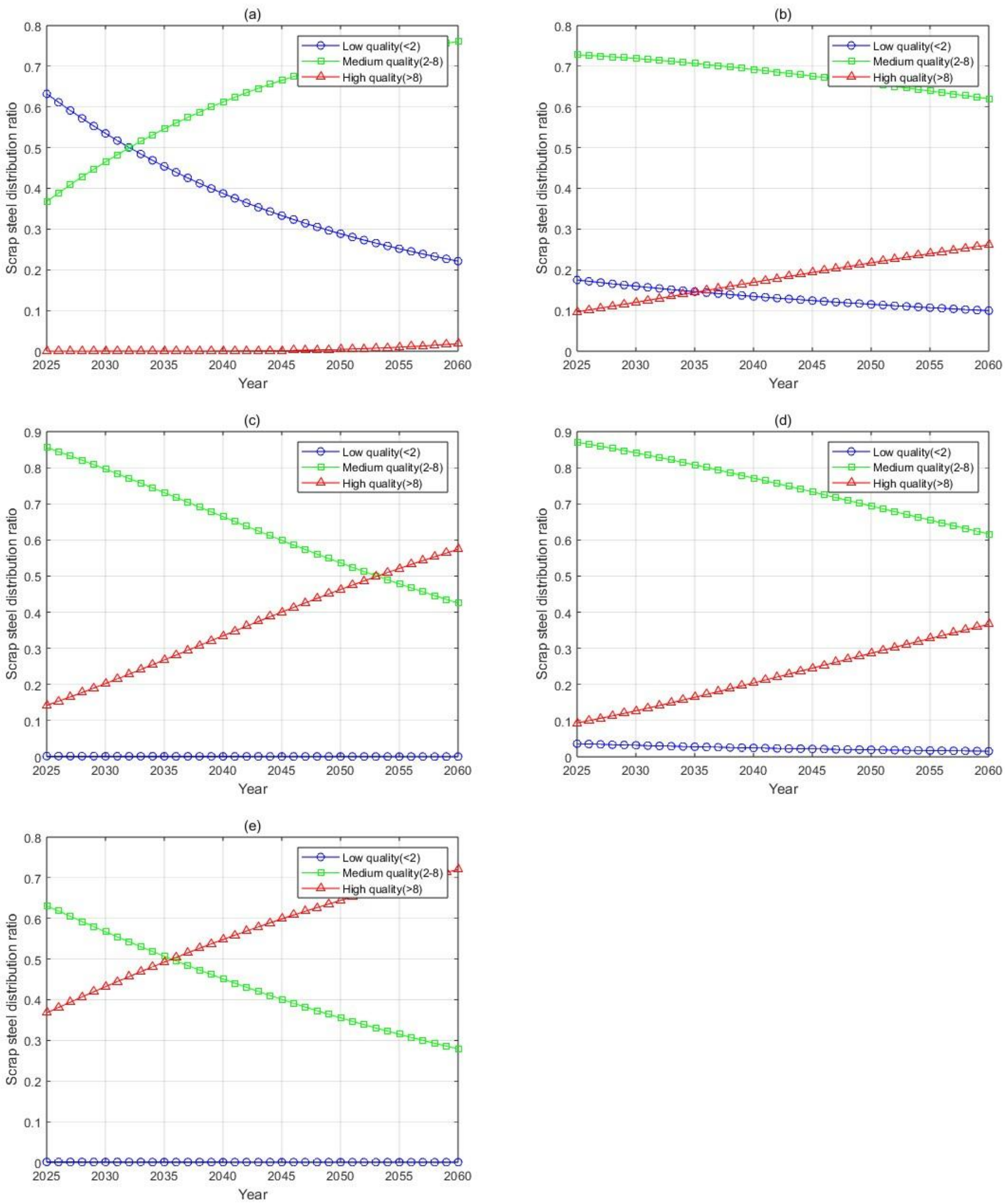

Figure S3. The proportional distribution of EOL scrap steel resources by sector (a) Building; (b) Machinery; (c) Power; (d) Appliance; (e) Automotive

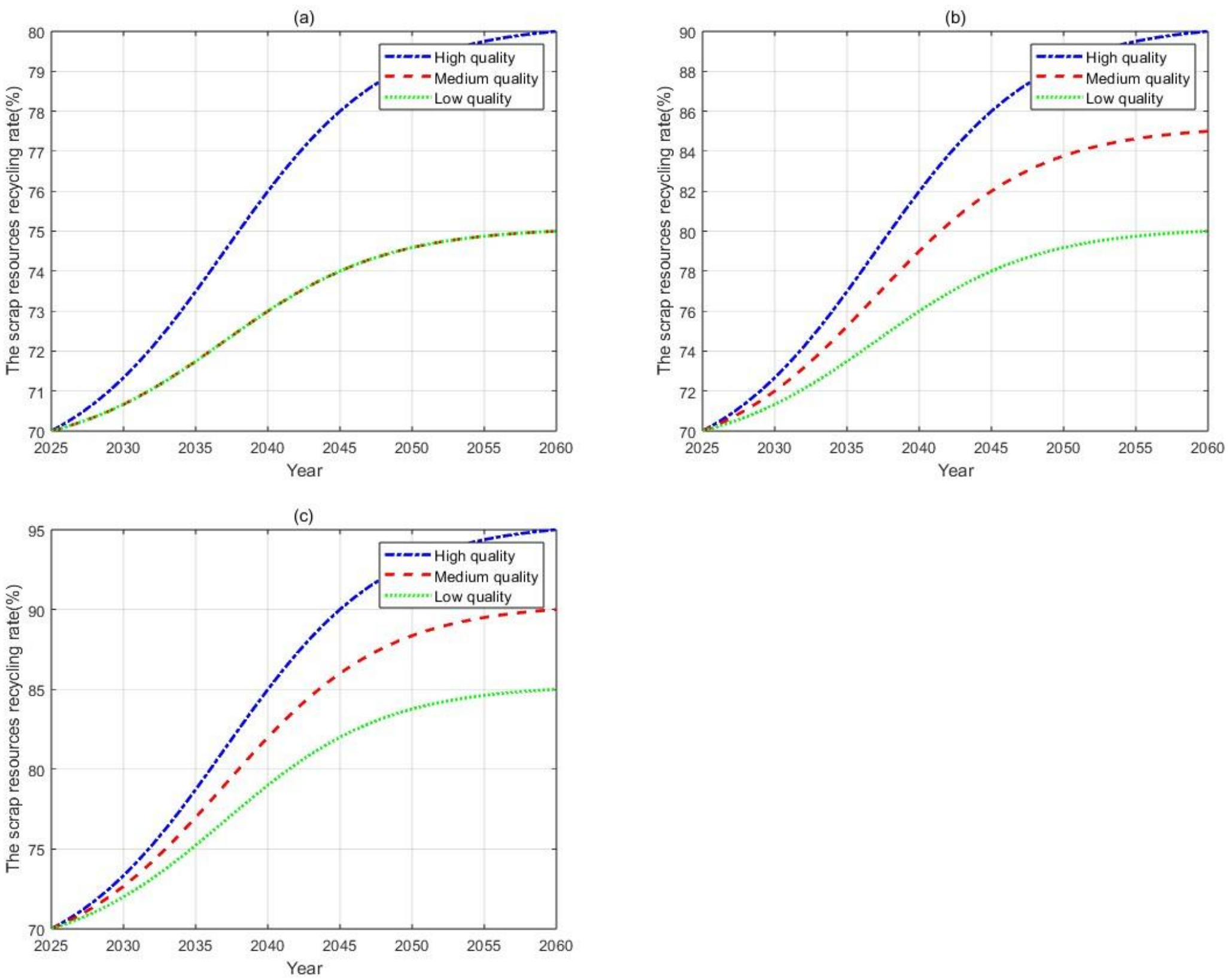

Figure S4. The scrap resources recycling rate in different scenarios. (a) Baseline scenario; (b) Accelerated scenario; (c) Ambitious scenario

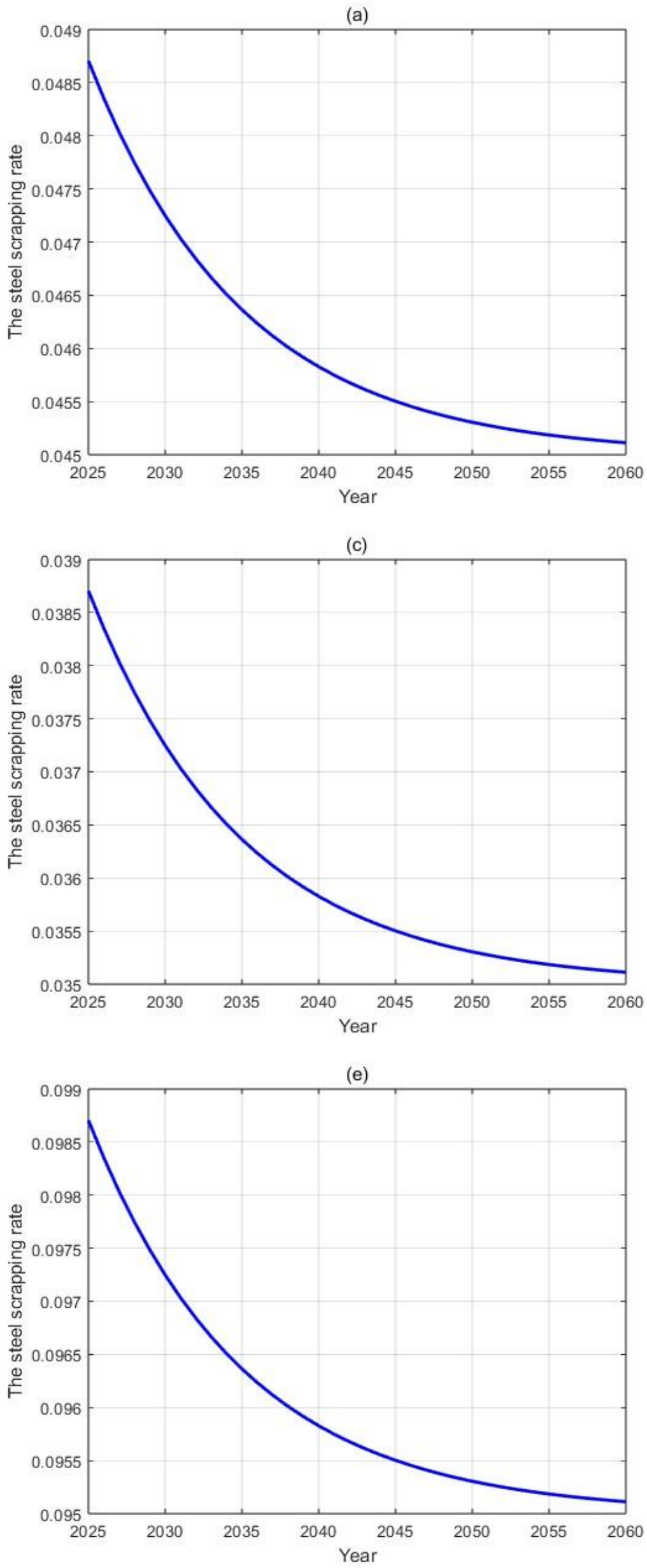

Figure S5. The steel scrapping rates in different end-use sectors. (a) Building; (b) Machinery; (c) Power; (d) Appliance; (e) Automotive

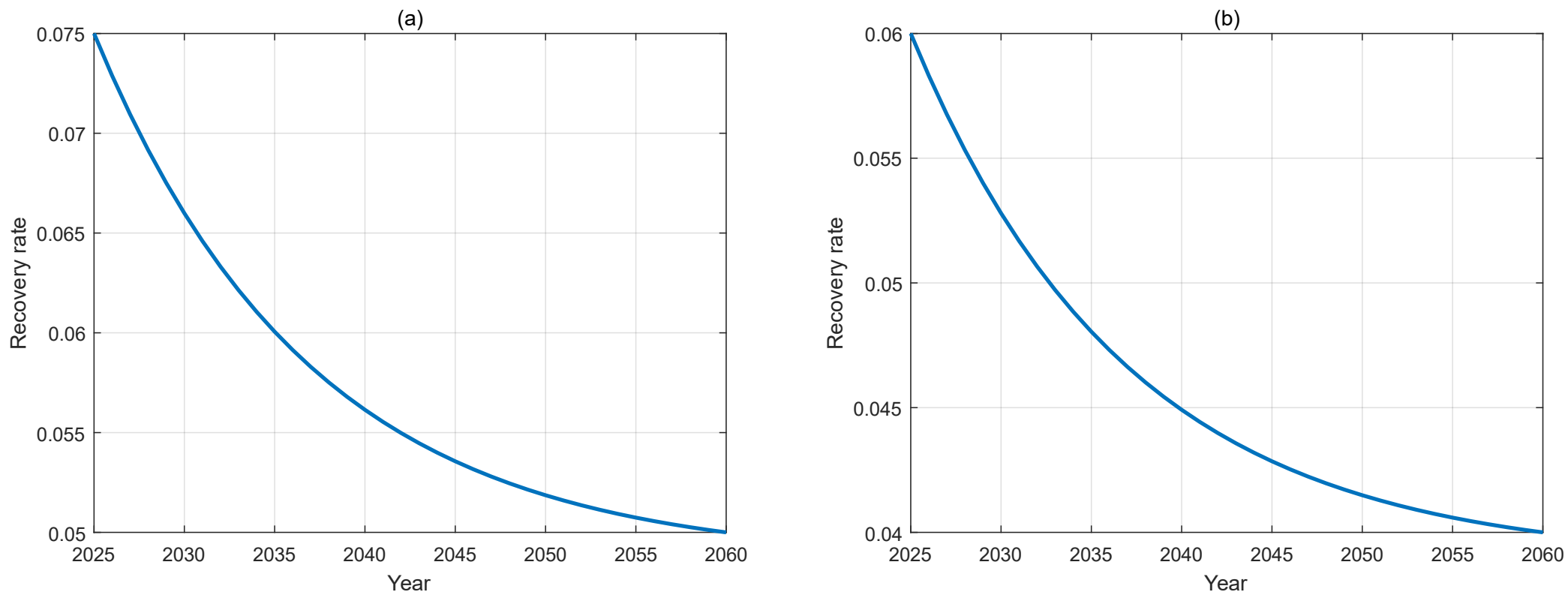

Figure S6. The specific time-series values of recovery rates. (a) Home scrap; (b) Prompt scrap

Table S1. Assessment system for provincial EAF development potential scores

Dimension	Indicator	Description	Effect
Resource supply	A1	Scrap self-sufficiency rate of steel plants	Positive
	A2	Provincial scrap supply rate	Positive
	A3	Availability of substitute materials	Positive
	A4	Provincial scrap supply volume	Positive
	A5	Water supply capacity	Positive
Energy supply	B1	Natural gas supply capacity	Positive
	B2	Electricity supply capacity	Positive
	B3	Coking coal supply capacity	Positive
	B4	Energy infrastructure construction	Negative
Economic conditions	C1	Scrap price	Negative
	C2	Electricity price	Negative
	C3	Labor cost	Negative
	C4	GDP per capita	Positive
	C5	Rebar price	Positive
Market demand	D1	Crude steel consumption	Positive
	D2	Crude steel production	Positive
Policy support	E1	Green transformation of steel industry	Positive
	E2	Environmental protection policy	Positive
	E3	Tax support	Negative
	E4	Electricity structure adjustment	Positive

Table S2. The classification of EAF equipment types

EAF Type	Approximate commissioning period	Typical electricity consumption (kWh/t)	Melting time (min)	Average lifespan (years)	Technical description
Type 1	1975–2005	600–650	120–150	30	Traditional AC EAF, low automation, high energy consumption
Type 2	1995–2025	550–600	90–120	30	Oxygen-fuel system, automatic charging, improved efficiency
Type 3	2015–2045	500–550	60–90	30	High-frequency DC, optimized furnace design, energy-saving device applications
Type 4	2035–2065	430–500	45–60	35	Intelligent EAF, energy management systems, smart melting control
Type 5	2055–2085	380–430	30–45	35	Zero-carbon EAF, hydrogen metallurgy, AI-driven full-process optimization

Table S3. Definition and representation of key scenario parameters

Parameter	Description	Role in the model	Associated equation	Data source / presentation
Main commissioning year	Central year of large-scale deployment for a given EAF generation	Determines timing of capacity expansion	Eq. (4)	Table S4
Construction peak width	Width of deployment period around the commissioning year	Determines deployment speed and diffusion pattern	Eq. (4)	Table S4
Average service life	Expected operational lifetime of EAF equipment	Determines retirement timing	Eq. (6)	Table S4
Weibull shape parameter	Shape parameter governing retirement probability distribution	Determines retirement dynamics	Eq. (6)	Table S4
Capacity utilization rate	Ratio of actual output to installed capacity	Determines EAF production level	Eq. (9)	Figure S1
Scrap recovery rate	Fraction of recoverable scrap entering the recycling system	Determines scrap availability	Eqs. (11)-(12)	Figure S6
Grid emission factor	Carbon intensity of electricity generation	Determines indirect EAF emissions	Eq. (22)	Figure 4

Table S4. The parameters of the capacity modeling of the five types of EAF steelmaking in different scenarios

(a) Baseline scenario

Province	Main commissioning years	Construction peak width	Average EAF lifetime	The shape parameter of Weibull distribution
$S_p \geq 40$	[1985, 2005, 2025, 2045, 2065]	[6, 6, 6, 7, 7]	[30, 30, 30, 35, 35]	[2.0, 2.5, 2.8, 3.0, 3.2]
$20 < S_p < 40$	[1990, 2010, 2030, 2050, 2070]	[6, 6, 6, 7, 7]	[30, 30, 30, 35, 35]	[2.0, 2.3, 2.6, 2.9, 3.1]
$S_p \leq 20$	[1995, 2015, 2035, 2055, 2075]	[6, 6, 6, 7, 7]	[30, 30, 30, 35, 35]	[1.8, 2.2, 2.5, 2.8, 3.0]

Note: (1) $S_p \geq 40$ provinces include Hebei, Guangdong, Shandong, Liaoning, Shanghai, Jiangsu, Heilongjiang, Shanxi, Ningxia, Inner Mongolia, and Xinjiang. (2) $20 < S_p < 40$ provinces include Sichuan, Anhui, Hubei, Yunnan, Gansu, Shaanxi, Zhejiang, and Fujian. (3) $S_p \leq 20$ provinces include Jilin, Guizhou, Hunan, Qinghai, Henan, Tianjin, Chongqing, Guangxi, Jiangxi, Tibet, Beijing, and Hainan.

(b) Accelerated scenario

Province	Main commissioning years	Construction peak width	Average EAF lifetime	The shape parameter of Weibull distribution
$S_p \geq 40$	[1975, 1995, 2015, 2035, 2055]	[6, 6, 6, 7, 7]	[30, 30, 30, 35, 35]	[2.0, 2.5, 2.8, 3.0, 3.2]
$20 < S_p < 40$	[1980, 2000, 2020, 2040, 2060]	[6, 6, 6, 7, 7]	[30, 30, 30, 35, 35]	[2.0, 2.3, 2.6, 2.9, 3.1]
$S_p \leq 20$	[1985, 2005, 2025, 2045, 2065]	[6, 6, 6, 7, 7]	[30, 30, 30, 35, 35]	[1.8, 2.2, 2.5, 2.8, 3.0]

(c) Ambitious scenario

Province	Main commissioning years	Construction peak width	Average EAF lifetime	The shape parameter of Weibull distribution
$S_p \geq 40$	[1970, 1990, 2010, 2030, 2050]	[6, 6, 6, 7, 7]	[30, 30, 30, 35, 35]	[2.0, 2.5, 2.8, 3.0, 3.2]
$20 < S_p < 40$	[1975, 1995, 2015, 2035, 2055]	[6, 6, 6, 7, 7]	[30, 30, 30, 35, 35]	[2.0, 2.3, 2.6, 2.9, 3.1]
$S_p \leq 20$	[1980, 2000, 2020, 2040, 2060]	[6, 6, 6, 7, 7]	[30, 30, 30, 35, 35]	[1.8, 2.2, 2.5, 2.8, 3.0]

Table S5. The province-level historical population (unit: ten thousand people)[1]

Province	2015	2016	2017	2018	2019	2020	2021	2022
Beijing	2188	2195	2194	2192	2190	2189	2189	2184
Tianjin	1439	1443	1410	1383	1385	1387	1373	1363
Hebei	7345	7375	7409	7426	7447	7464	7448	7420
Shanxi	3519	3514	3510	3502	3497	3490	3480	3481
Inner Mongolia	2440	2436	2433	2422	2415	2403	2400	2401
Liaoning	4338	4327	4312	4291	4277	4255	4229	4197
Jilin	2613	2567	2526	2484	2448	2399	2375	2348
Heilongjiang	3629	3463	3399	3327	3255	3171	3125	3099
Shanghai	2458	2467	2466	2475	2481	2488	2489	2475
Jiangsu	8315	8381	8423	8446	8469	8477	8505	8515
Zhejiang	5985	6072	6170	6273	6375	6468	6540	6577
Anhui	6011	6033	6057	6076	6092	6105	6113	6127
Fujian	3984	4016	4065	4104	4137	4161	4187	4188
Jiangxi	4485	4496	4511	4513	4516	4519	4517	4528
Shandong	9866	9973	10033	10077	10106	10165	10170	10163
Henan	9701	9778	9829	9864	9901	9941	9883	9872
Hubei	5850	5885	5904	5917	5927	5745	5830	5844
Hunan	6615	6625	6633	6635	6640	6645	6622	6604
Guangdong	11678	11908	12141	12348	12489	12624	12684	12657
Guangxi	4811	4857	4907	4947	4982	5019	5037	5047
Hainan	945	957	972	982	995	1012	1020	1027
Chongqing	3070	3110	3144	3163	3188	3209	3212	3213
Sichuan	8196	8251	8289	8321	8351	8371	8372	8374
Guizhou	3708	3758	3803	3822	3848	3868	3852	3856
Yunnan	4663	4677	4693	4703	4714	4722	4690	4693
Tibet	330	340	349	354	361	366	366	364
Shaanxi	3793	3813	3835	3864	3876	3953	3954	3956
Gansu	2600	2610	2626	2637	2647	2502	2490	2492
Qinghai	588	593	598	603	608	592	594	595
Ningxia	668	675	682	688	695	720	725	728
Xinjiang	2360	2398	2445	2487	2523	2585	2589	2587

Table S6. The province-level historical per capita GDP (unit: ten thousand yuan per person)[1]

Province	2022	2021	2020	2019	2018	2017	2016	2015
Beijing	19.0	18.4	16.5	16.4	14.0	12.9	11.8	10.6
Tianjin	11.8	11.4	10.1	10.2	9.6	8.7	8.0	7.6
Hebei	5.7	5.4	4.8	4.7	4.4	4.1	3.9	3.6
Shanxi	7.4	6.6	5.1	4.8	4.6	4.1	3.4	3.4
Inner Mongolia	9.6	8.5	7.2	7.1	6.6	6.1	5.7	5.3
Liaoning	6.9	6.5	5.9	5.7	5.5	5.0	4.7	4.6
Jilin	5.5	5.5	5.1	4.3	4.5	4.3	4.0	3.8
Heilongjiang	5.1	4.7	4.3	3.6	3.8	3.6	3.4	3.3
Shanghai	18.0	17.4	15.6	15.7	13.5	12.7	11.7	10.4
Jiangsu	14.3	13.8	12.1	11.7	11.1	10.2	9.3	8.9
Zhejiang	11.8	11.4	10.1	9.9	9.3	9.2	7.8	7.3
Anhui	7.4	7.0	6.2	6.1	5.6	4.9	4.4	4.0
Fujian	12.7	11.7	10.5	10.3	9.5	8.4	7.4	6.8
Jiangxi	7.1	6.6	5.7	5.5	4.9	4.4	4.0	3.7
Shandong	8.6	8.2	7.2	7.0	6.6	6.3	5.9	5.6
Henan	5.9	5.9	5.5	5.4	5.1	4.6	4.1	3.8
Hubei	9.2	8.7	7.4	7.7	7.1	6.3	5.7	5.2
Hunan	7.4	6.9	6.3	6.0	5.5	5.1	4.7	4.3
Guangdong	10.2	9.9	8.9	8.7	8.2	7.6	7.0	6.5
Guangxi	5.2	5.0	4.4	4.3	4.0	3.6	3.3	3.1
Hainan	6.7	6.4	5.5	5.4	5.0	4.7	4.3	4.0
Chongqing	9.1	8.7	7.8	7.4	6.8	6.4	5.8	5.2
Sichuan	6.8	6.5	5.8	5.6	5.2	4.6	4.0	3.7
Guizhou	5.2	5.0	4.6	4.4	4.0	3.6	3.2	2.9
Yunnan	6.2	5.8	5.2	4.9	4.4	3.9	3.5	3.2
Tibet	5.8	5.7	5.2	4.9	4.3	3.9	3.5	3.2
Shaanxi	8.3	7.6	6.6	6.6	6.1	5.5	4.9	4.7
Gansu	4.5	4.1	3.6	3.5	3.2	2.9	2.7	2.6
Qinghai	6.1	5.7	5.1	5.0	4.7	4.2	3.9	3.5
Ningxia	7.0	6.3	5.5	5.3	5.0	4.6	4.0	3.8
Xinjiang	6.9	6.2	5.4	5.4	4.9	4.5	4.1	4.0

Table S7. The province-level future population (unit: ten thousand people)[2]

Province	2025	2030	2035	2040	2045	2050	2055	2060
Beijing	2216.2	2230.8	2220.1	2175.6	2109.8	2031.6	1936.4	1832.5
Tianjin	1388.0	1381.3	1364.4	1333.3	1290.3	1237.2	1172.3	1103.7
Hebei	7402.6	7298.3	7211.5	7121.7	6983.3	6753.5	6424.2	6074.4
Shanxi	3479.7	3449.5	3402.9	3329.5	3226.2	3090.8	2919.3	2735.9
Inner Mongolia	2385.8	2348.5	2297.5	2228.2	2140.5	2033.6	1906.4	1773.8
Liaoning	4148.6	4010.7	3847.5	3647.6	3412.9	3151.7	2869.2	2590.3
Jilin	2350.5	2278.6	2195.0	2093.0	1969.2	1826.9	1669.9	1512.9
Heilongjiang	3069.9	2937.1	2790.3	2619.1	2419.4	2197.3	1960.6	1728.7
Shanghai	2504.9	2508.2	2486.7	2433.0	2354.2	2256.1	2137.9	2011.5
Jiangsu	8415.4	8295.7	8136.4	7932.8	7673.4	7339.0	6915.6	6455.8
Zhejiang	6526.7	6563.4	6560.1	6493.7	6366.6	6177.9	5912.7	5604.9
Anhui	6064.2	6000.6	5938.3	5860.2	5750.6	5583.6	5340.5	5070.8
Fujian	4198.3	4217.2	4228.1	4224.5	4198.8	4129.4	4000.1	3841.4
Jiangxi	4491.9	4470.8	4481.6	4485.5	4442.9	4331.1	4152.7	3965.7
Shandong	10101.5	10004.6	9900.5	9758.5	9571.5	9312.9	8930.6	8502.6
Henan	9865.4	9771.0	9734.1	9715.9	9639.8	9427.7	9061.0	8660.3
Hubei	5703.6	5588.3	5445.9	5272.8	5068.8	4823.6	4522.5	4203.2
Hunan	6522.7	6385.5	6272.4	6155.5	5999.8	5771.5	5458.7	5129.6
Guangdong	12964.0	13280.9	13523.6	13656.1	13700.3	13633.5	13402.7	13078.6
Guangxi	5044.1	5081.1	5166.8	5269.0	5353.4	5379.9	5332.8	5275.0
Hainan	1030.8	1050.4	1069.6	1083.6	1090.6	1088.0	1073.2	1053.6
Chongqing	3164.3	3117.4	3065.1	2990.9	2890.3	2764.1	2609.3	2449.0
Sichuan	8267.5	8147.3	8013.8	7827.6	7576.1	7253.5	6853.0	6435.3
Guizhou	3912.1	3991.1	4107.3	4216.6	4301.6	4351.8	4368.5	4400.0
Yunnan	4732.8	4754.0	4788.9	4793.5	4757.3	4672.9	4535.9	4390.2
Tibet	380.2	395.8	413.3	429.6	443.3	452.7	457.1	460.4
Shaanxi	3952.6	3922.6	3867.5	3788.5	3690.6	3564.1	3393.0	3202.5
Gansu	2501.7	2491.1	2481.2	2463.0	2431.8	2379.1	2298.4	2213.7
Qinghai	600.6	608.4	617.2	622.6	622.5	615.2	599.9	582.5
Ningxia	735.0	748.5	762.8	772.6	776.5	773.1	760.5	745.1
Xinjiang	2595.2	2596.7	2599.7	2603.3	2587.8	2528.9	2423.6	2300.5

Table S8. The province-level future GDP (unit: ten billion yuan)[2]

Province	2025	2030	2035	2040	2045	2050	2055	2060
Beijing	280.8	287.9	293.9	299.0	303.5	307.6	311.4	315.1
Tianjin	224.7	234.8	245.6	256.5	266.8	276.0	283.7	290.1
Hebei	511.8	540.3	566.5	590.9	614.0	636.0	657.5	678.7
Shanxi	150.7	155.4	159.9	164.4	168.8	173.3	177.9	182.6
Inner Mongolia	285.8	295.3	303.3	310.3	317.1	324.3	332.3	340.9
Liaoning	379.7	392.9	403.8	413.3	422.0	430.9	440.5	450.8
Jilin	167.9	173.3	178.4	183.4	188.5	194.0	199.9	206.1
Heilongjiang	187.4	192.1	195.3	197.7	199.7	201.8	204.4	207.4
Shanghai	390.1	411.8	434.2	455.9	475.7	492.2	504.5	513.6
Jiangsu	1551.9	1608.6	1656.6	1697.7	1733.8	1767.1	1799.0	1830.2
Zhejiang	749.4	796.2	850.3	908.2	966.4	1021.3	1070.0	1112.9
Anhui	303.1	312.2	319.9	326.9	333.6	340.7	348.7	357.7
Fujian	360.9	381.5	401.2	420.7	440.9	462.5	486.1	511.3
Jiangxi	242.3	252.1	260.2	267.4	274.4	281.9	290.4	299.8
Shandong	1079.7	1176.5	1267.8	1354.5	1437.4	1517.5	1595.6	1673.3
Henan	790.5	823.6	853.9	882.9	911.9	942.5	975.8	1012.0
Hubei	466.1	486.5	503.8	518.6	531.8	544.1	556.3	568.4
Hunan	378.0	390.4	402.5	414.6	426.6	438.8	451.0	463.7
Guangdong	1001.5	1042.7	1085.7	1130.1	1175.6	1221.8	1268.3	1314.6
Guangxi	188.2	192.6	196.2	199.2	201.8	204.4	207.1	210.0
Hainan	56.2	59.0	61.8	64.4	66.8	68.9	70.6	72.0
Chongqing	224.0	232.4	239.4	245.2	249.9	253.5	256.2	258.3
Sichuan	520.9	545.5	569.0	591.5	612.7	632.4	650.7	667.4
Guizhou	128.8	130.3	131.3	132.0	132.4	132.6	132.6	132.7
Yunnan	211.9	219.5	226.3	232.4	237.8	242.8	247.3	251.7
Tibet	13.9	14.3	14.6	14.9	15.1	15.2	15.3	15.3
Shaanxi	204.2	209.8	215.0	220.2	225.7	231.8	238.8	246.5
Gansu	89.1	92.9	97.1	101.5	105.9	110.1	114.1	117.9
Qinghai	31.4	32.6	33.8	34.9	35.9	36.7	37.3	37.6
Ningxia	36.2	37.8	39.2	40.6	41.9	43.2	44.6	46.0
Xinjiang	111.7	115.4	119.1	122.8	126.5	130.6	134.9	139.4

Table S9. The historical in-use stocks of various steel-using products by sector

End-use sector	Steel-using product	Item	Data source
Building	Residential building, public building, and industrial building	Year-end usable building area	China Urban-Rural Construction Statistical Yearbook
Machinery	Agricultural machinery, construction machinery	/	/
Power	(1) Hydropower, thermal power, nuclear power, wind power, solar photovoltaic, and others; (2) Above 330 kV, 330 kV, 220 kV, 110 kV, 35 kV, and cables	Installed capacity, transmission distance	China Electricity Yearbook
Appliance	Washing machine, refrigerator, microwave oven, television, air conditioner, and heater	Average number of major durable consumer goods per 100 households at year-end	Provincial statistical yearbooks, such as the Beijing Statistical Yearbook
Automotive	Passenger vehicles, motorcycles, and electric-assisted bicycles	Average number of major durable consumer goods per 100 households at year-end	Provincial statistical yearbooks, such as the Beijing Statistical Yearbook

Table S10. The steel consumption coefficients of different steel products in different end-use sectors

End-use sector	Steel product	Steel consumption coefficient	Unit
Building	Residential building	28	kg/m^2
	Public building	52	kg/m^2
	Industrial building	52	kg/m^2
Machinery	Agricultural machinery	/	/
	Construction machinery	/	/
Power generation	Hydropower	35	kg/kw
	Thermal power	18	kg/kw
	Nuclear power	40	kg/kw
	Wind power	115	kg/kw
	Solar photovoltaic	50	kg/kw
	Other	30	kg/kw
Power transmission	Above 330 kV	42	t/km
	330 kV	30	t/km
	220 kV	20	t/km
	110 kV	12	t/km
	35 kV and cable	5	t/km
Appliance	Washing machine	0.018	t/unit
	Refrigerator	0.03	t/unit
	Microwave oven	0.008	t/unit
	Television	0.0008	t/unit
	Air conditioner	0.026	t/unit
	Heater	0.012	t/unit
Automotive	Passenger vehicles	0.94	t/unit
	Motorcycles	0.085	t/unit
	Electric-assisted bicycles	0.085	t/unit

Table S11. The material and energy flows of scrap- EAF route by EAF type

Type	Unit	Basic EAF	Conventional EAF	Technology-upgraded EAF	Smart-controlled EAF	Green EAF
Electricity	kWh/t	991.12	910.20	829.27	748.34	647.42
Coke	kg/t	23.42	22.75	22.30	21.63	21.19
Coal	kg/t	20.06	19.48	19.10	18.53	18.15
Oxygen	m^3/t	37.91	36.82	36.10	35.02	34.30

Table S12. Parameter settings and variation ranges used in the sensitivity analysis

Parameter	Base value / assumption	Variation range	Description
Scrap recovery rate	Time-dependent (scenario-based baseline values)	±0.5%	Reflects uncertainty in scrap collection and recovery efficiency under existing industrial structure
EAF service life	30–35 years (technology-dependent)	±2 years	Accounts for variability in equipment lifespan due to operational and maintenance differences
Weibull shape parameter	Scenario-specific baseline values	±0.2	Represents uncertainty in retirement distribution of EAF capacity
Capacity utilization rate	Scenario-dependent baseline values	±5%	Captures fluctuations in production intensity and market-driven operational adjustments
Scrap input–output ratio	1.07–1.30	Fixed range perturbation	Reflects variation in material efficiency and process conditions in EAF steelmaking

Section 2. Results

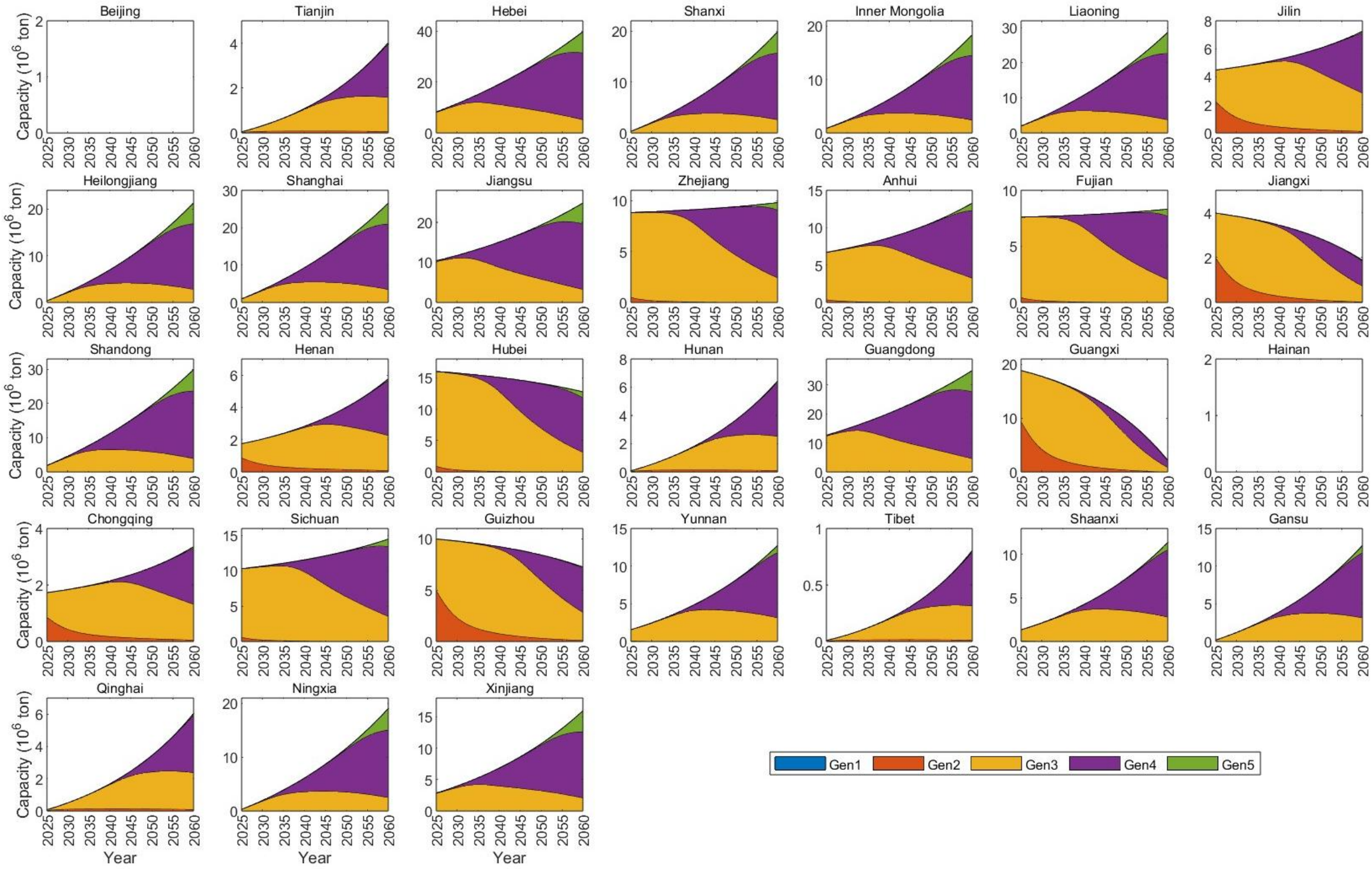

Figure S7. The EAF capacity trends of China by type in baseline scenario

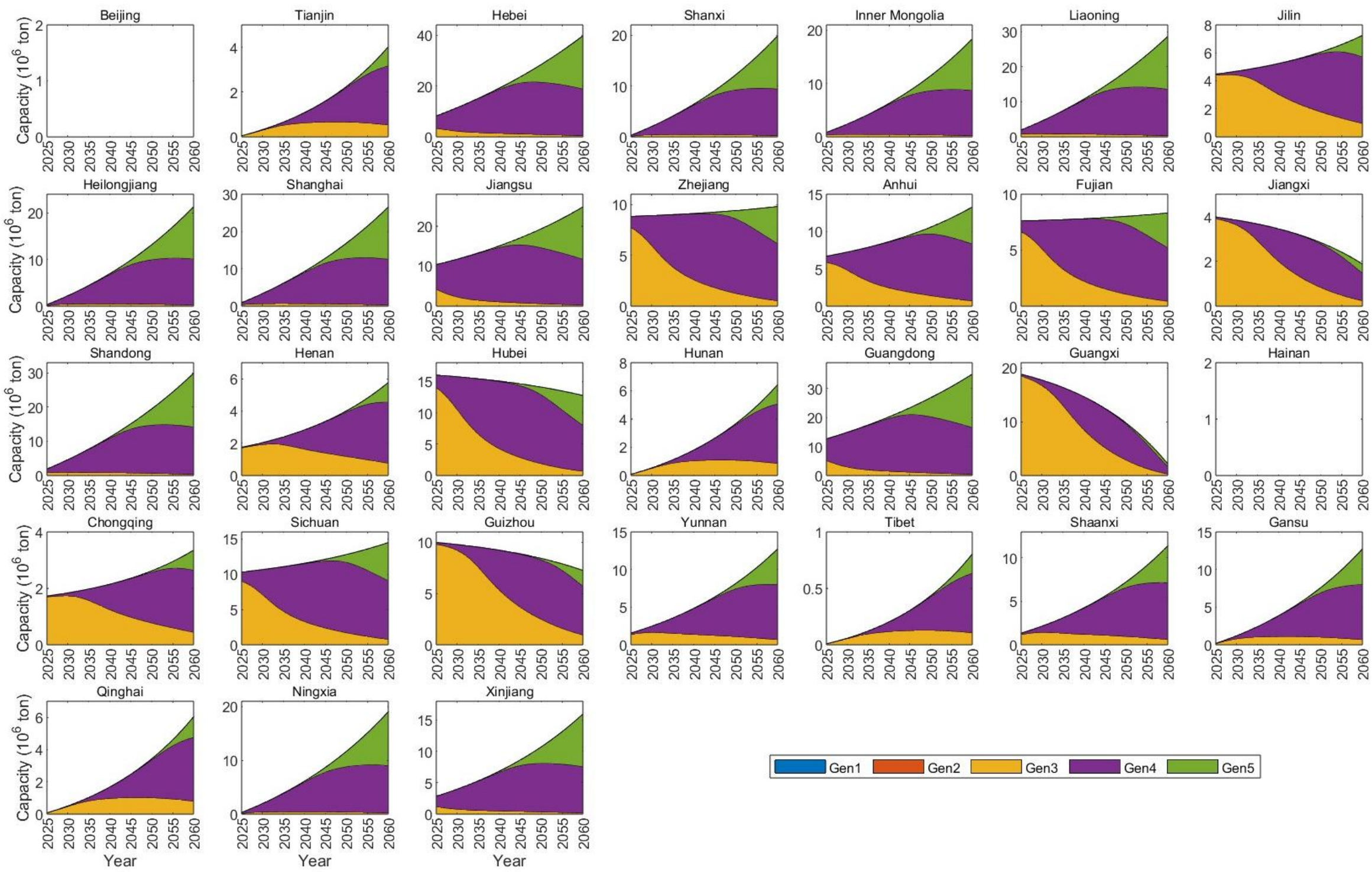

Figure S8. The EAF capacity trends of China by type in accelerated scenario

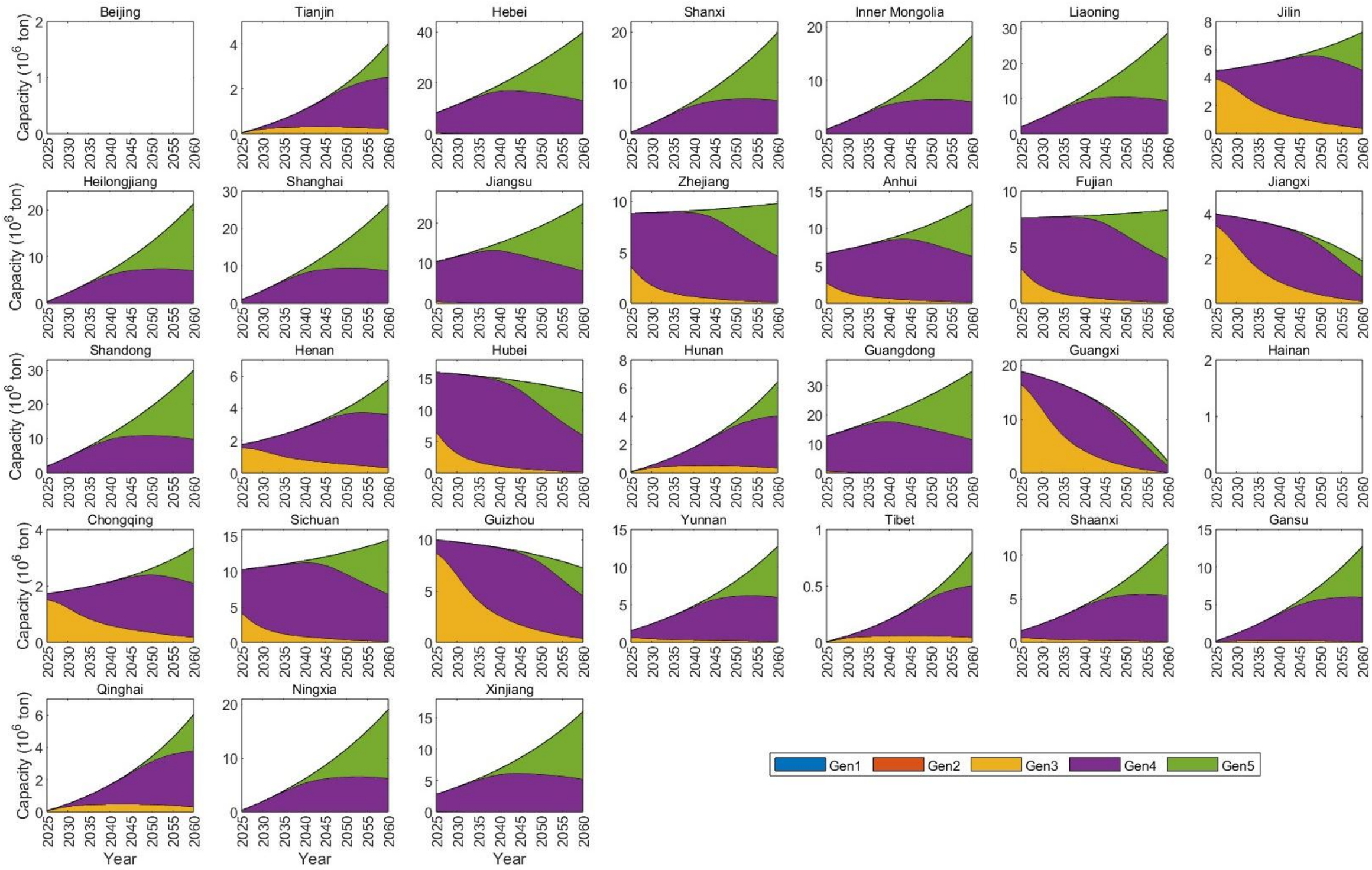

Figure S9. The EAF capacity trends of China by type in ambitious scenario

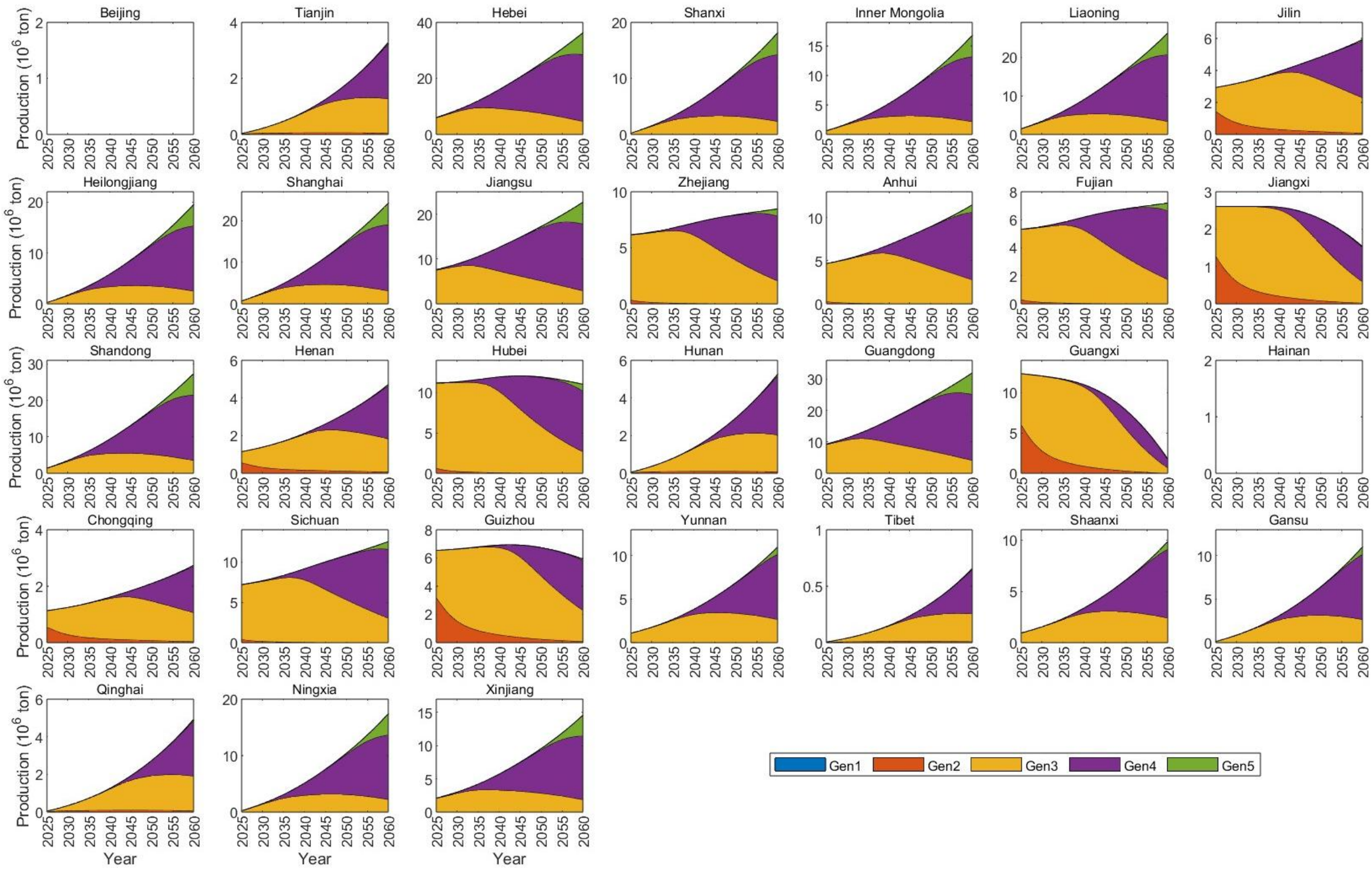

Figure S10. The EAF production trends of China by type in baseline scenario

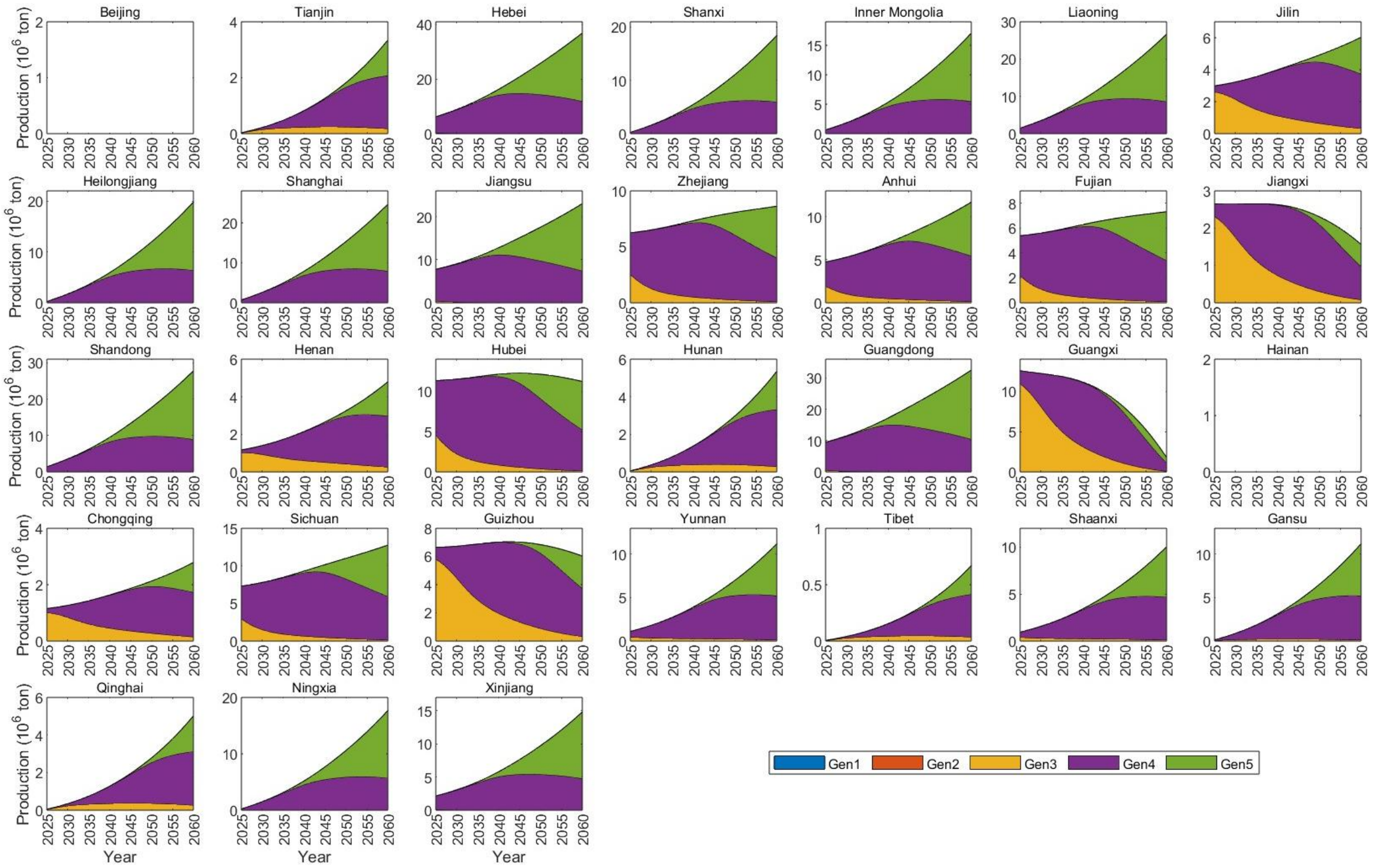

Figure S11. The EAF production trends of China by type in accelerated scenario

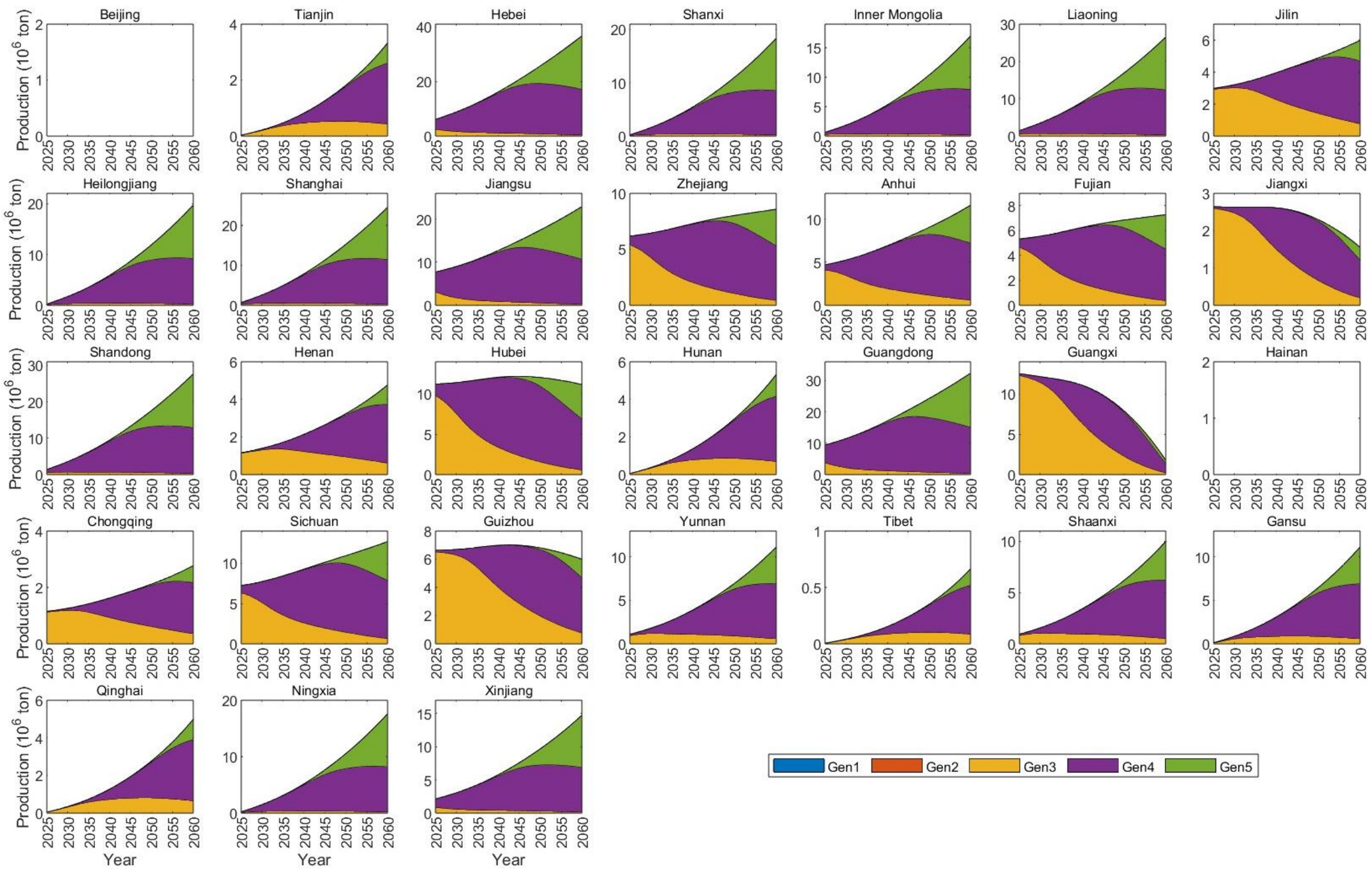

Figure S12. The EAF production trends of China by type in ambitious scenario

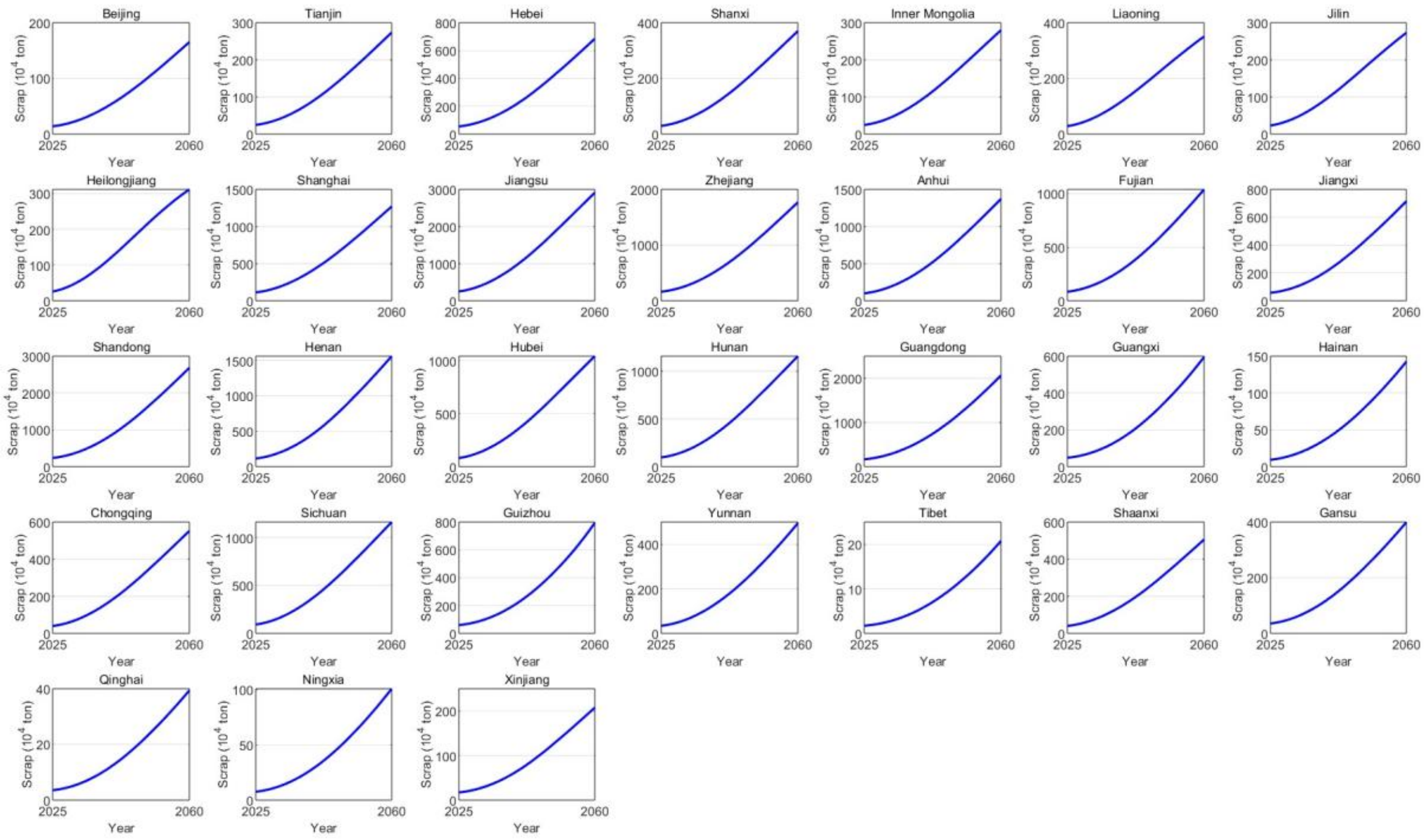

Figure S13. The EOL scrap steel generated by building industry in each province

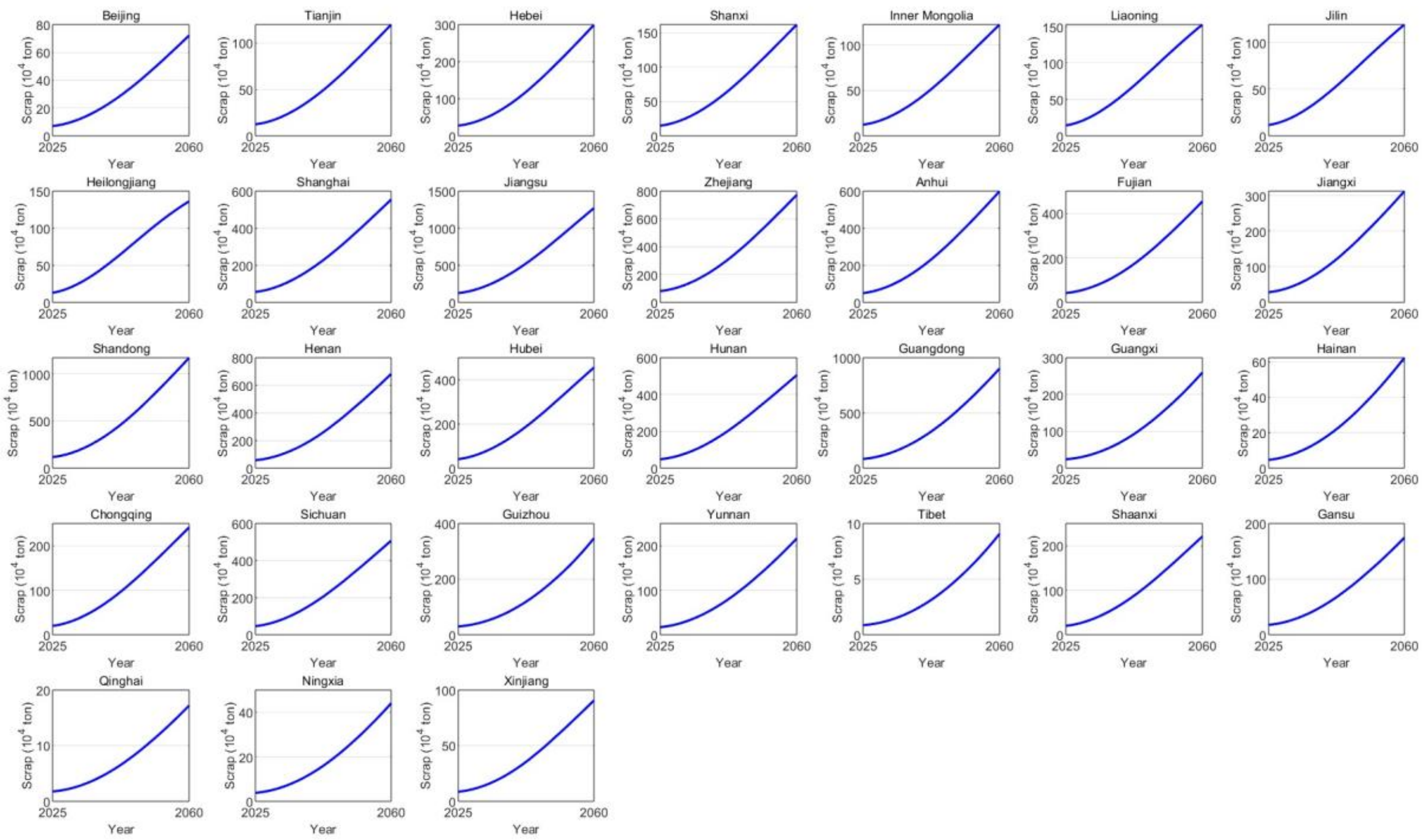

Figure S14. The EOL scrap steel generated by machine industry in each province

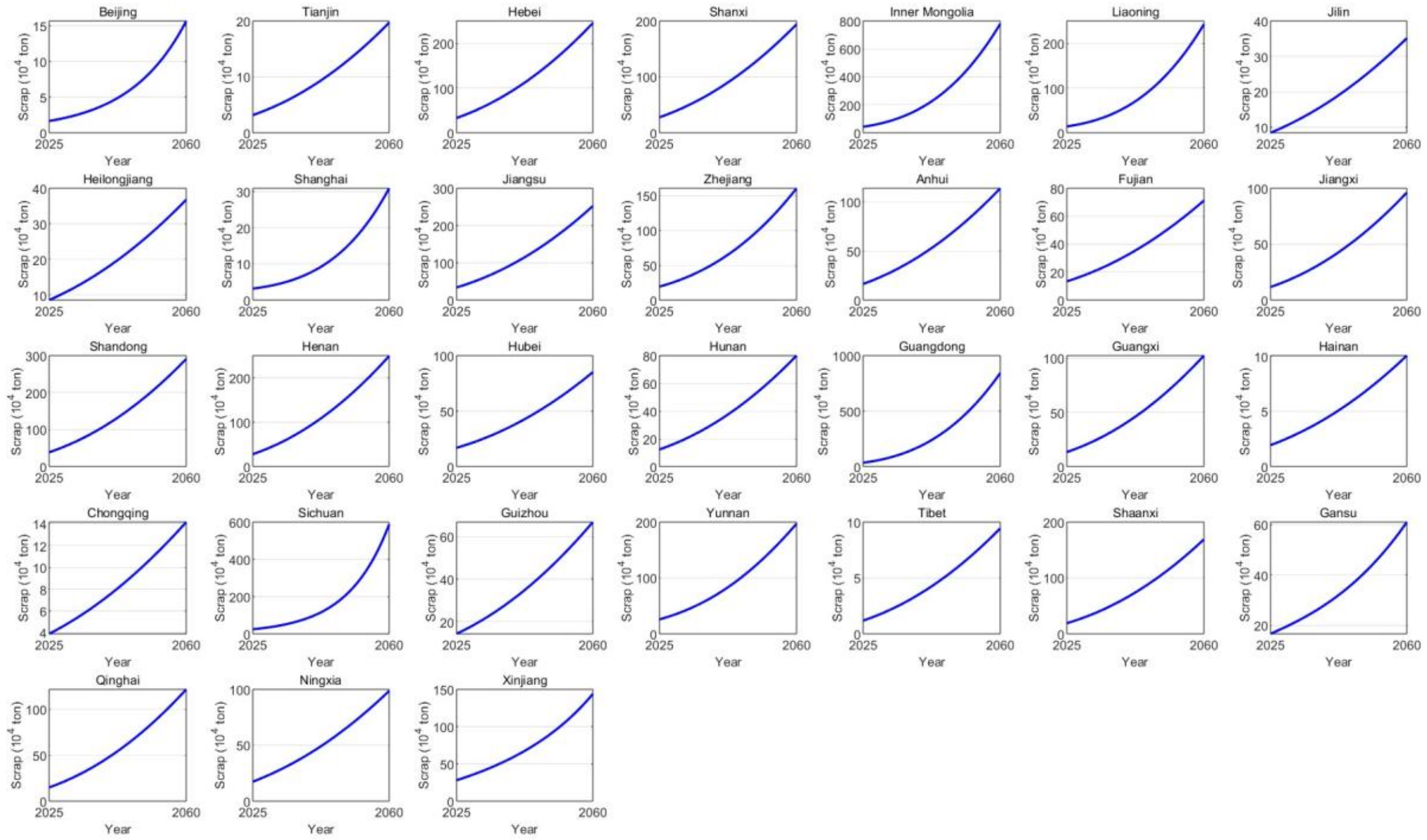

Figure S15. The EOL scrap steel generated by power infrastructure in each province

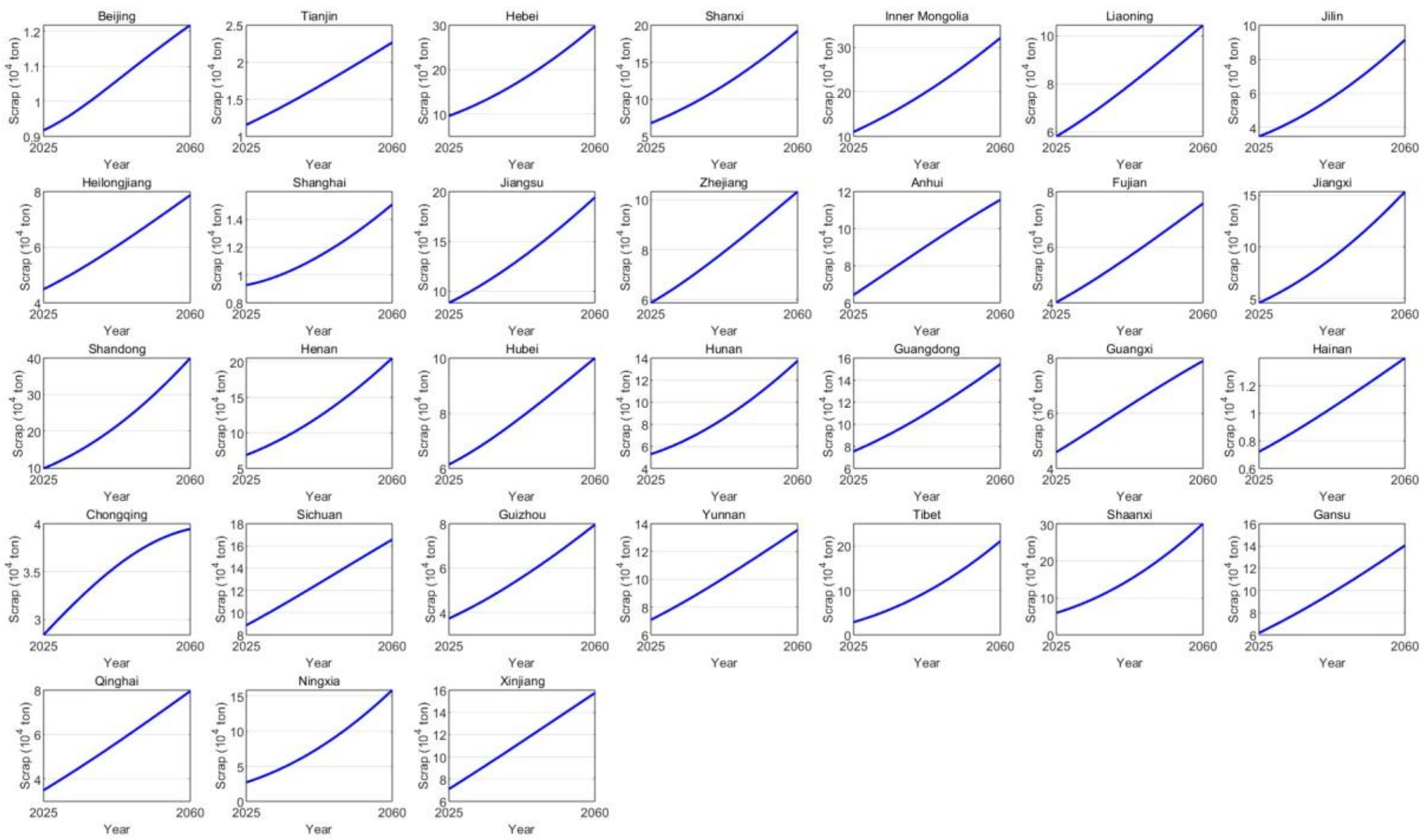

Figure S16. The EOL scrap steel generated by power transformation in each province

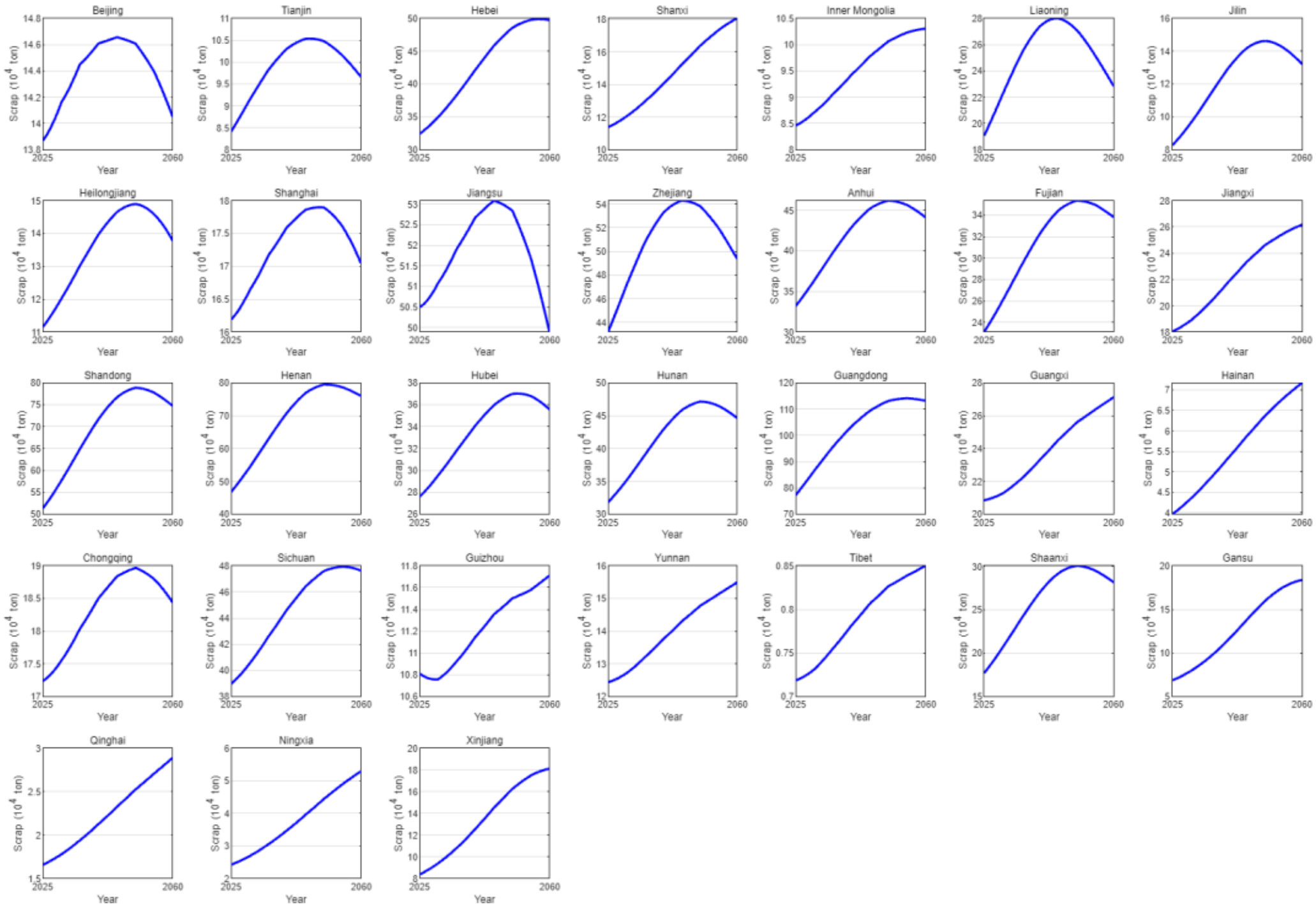

Figure S17. The EOL scrap steel generated by appliance industry in each province

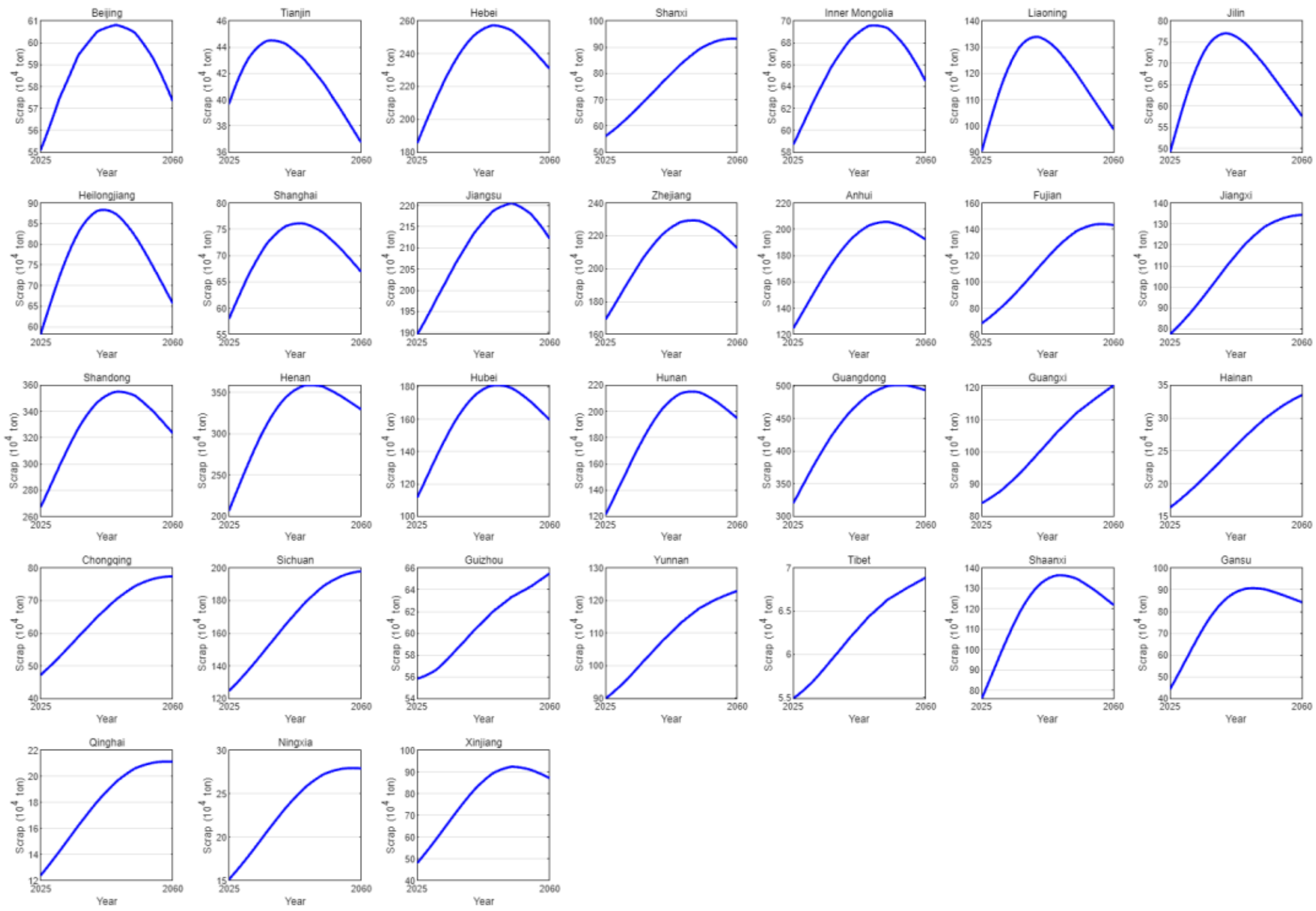

Figure S18. The EOL scrap steel generated by automotive industry in each province

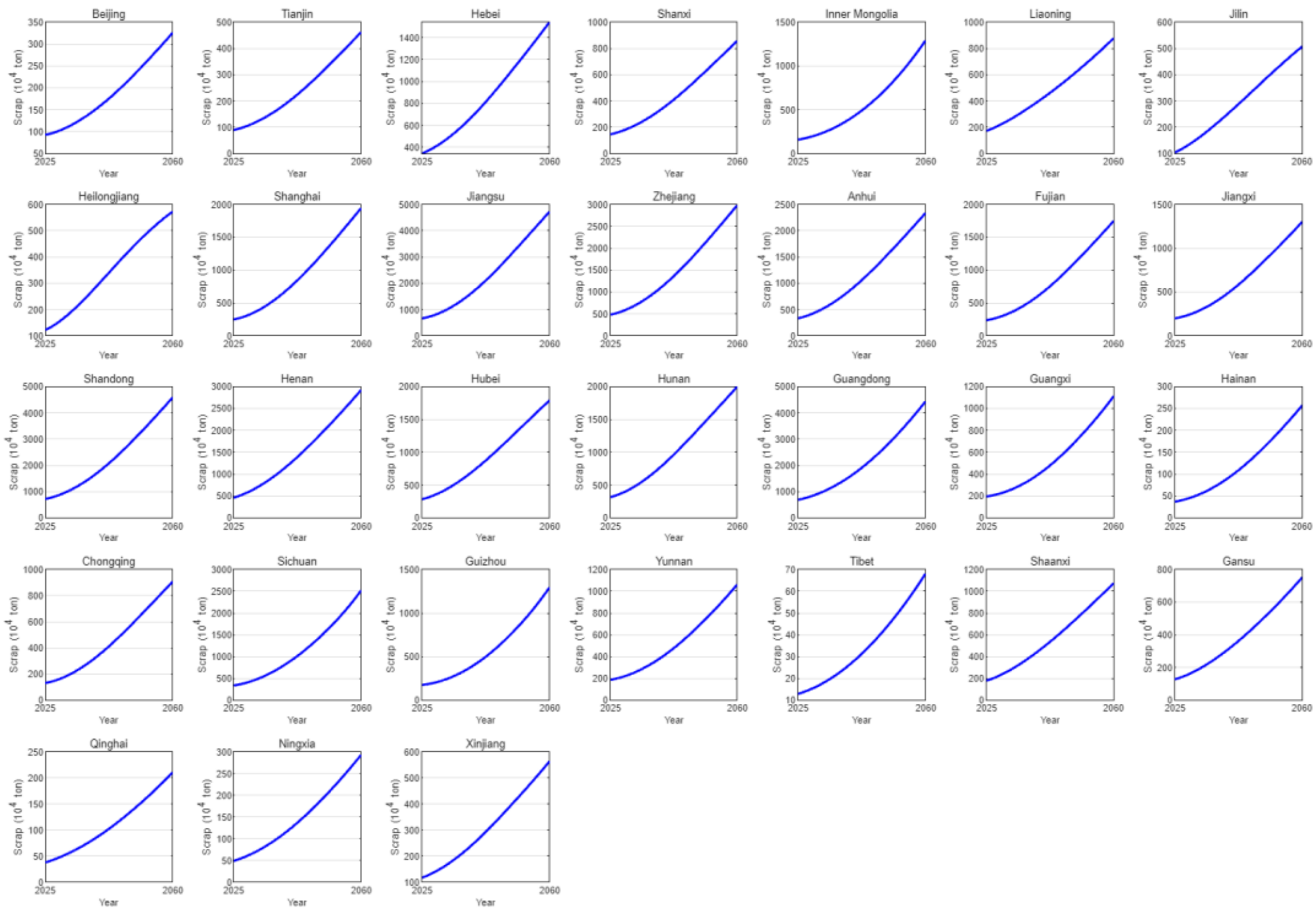

Figure S19. The total EOL scrap steel in each province

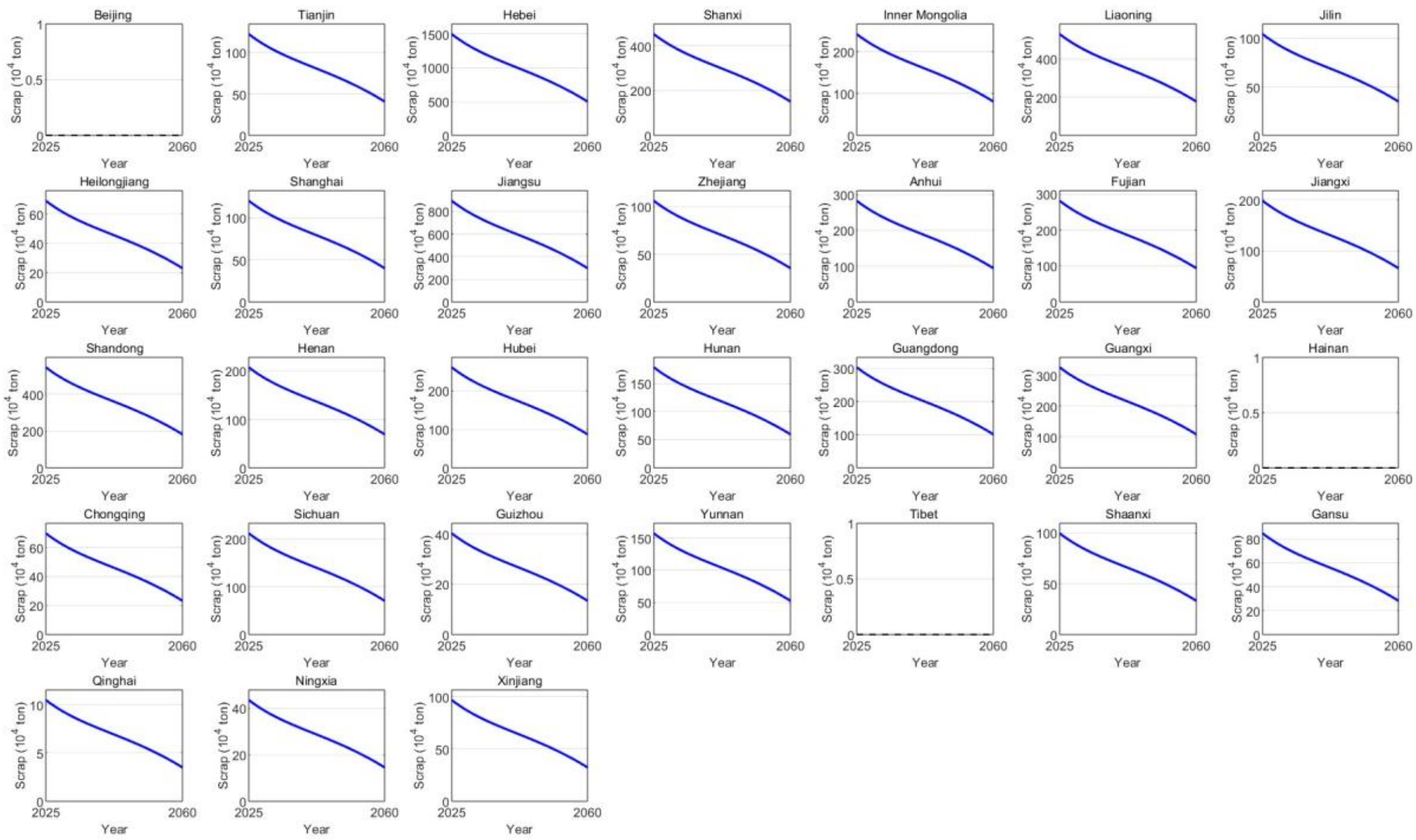

Figure S20. The home scrap steel in each province

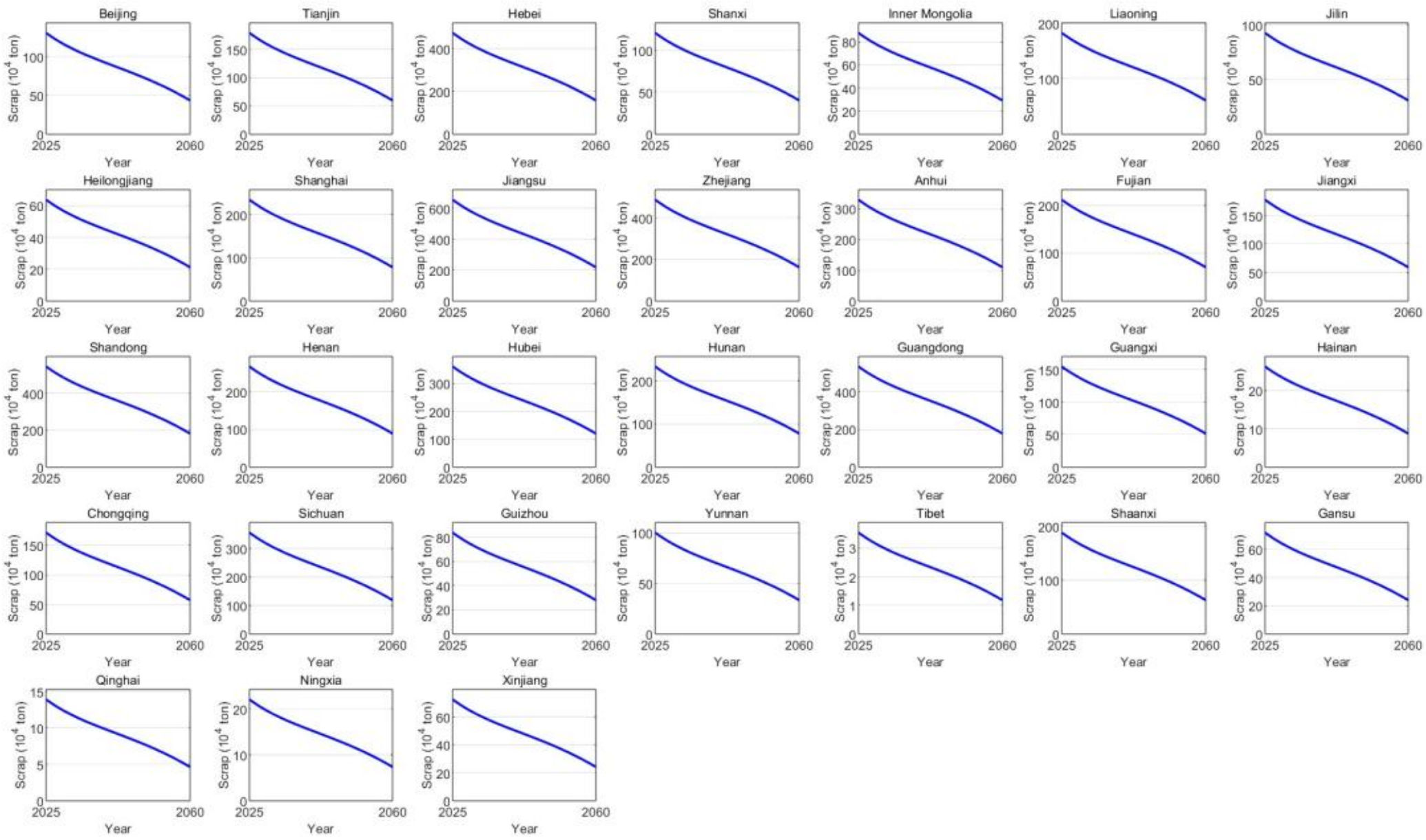

Figure S21. The prompt scrap steel in each province

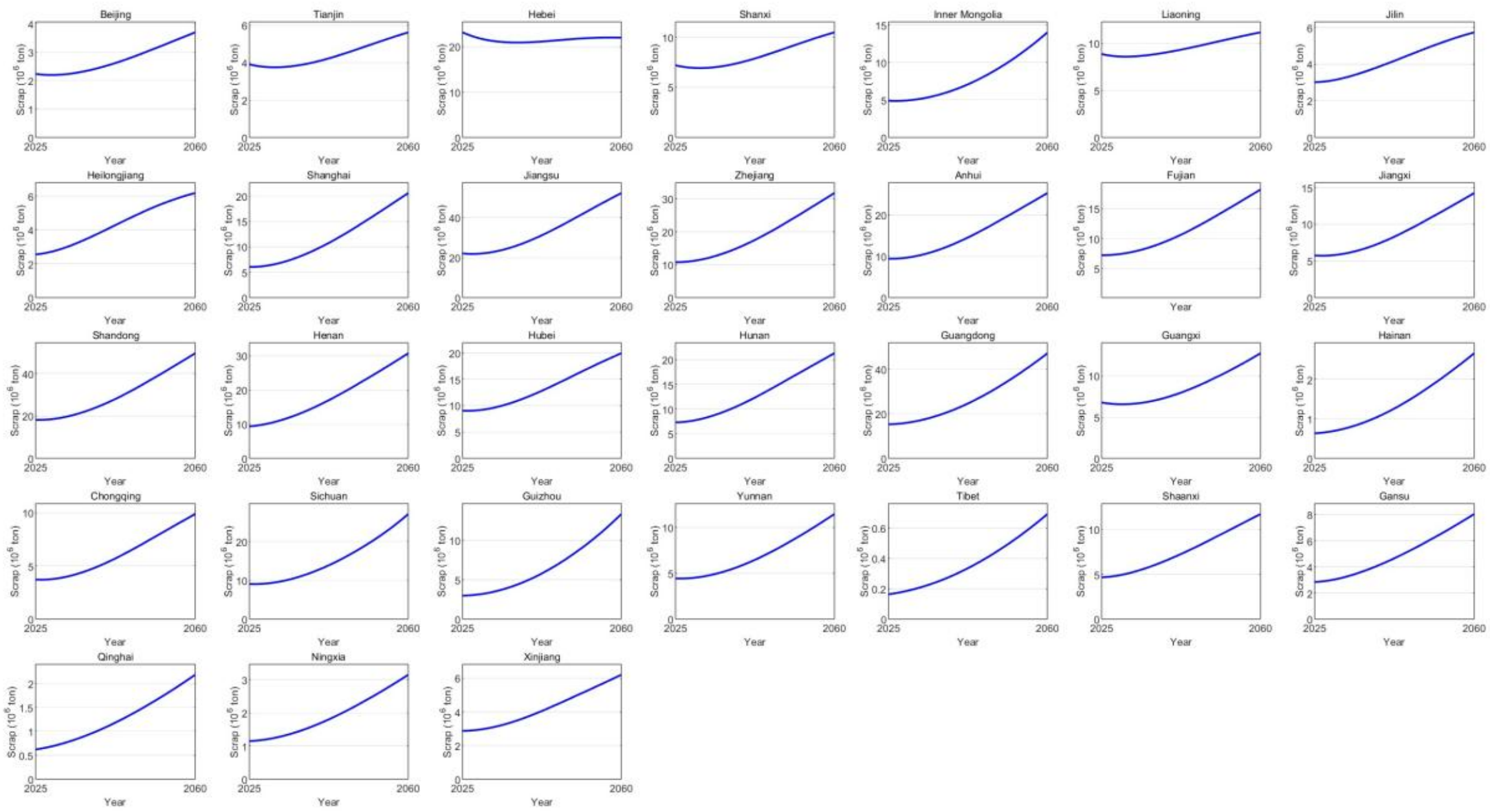

Figure S22. The total scrap steel in each province

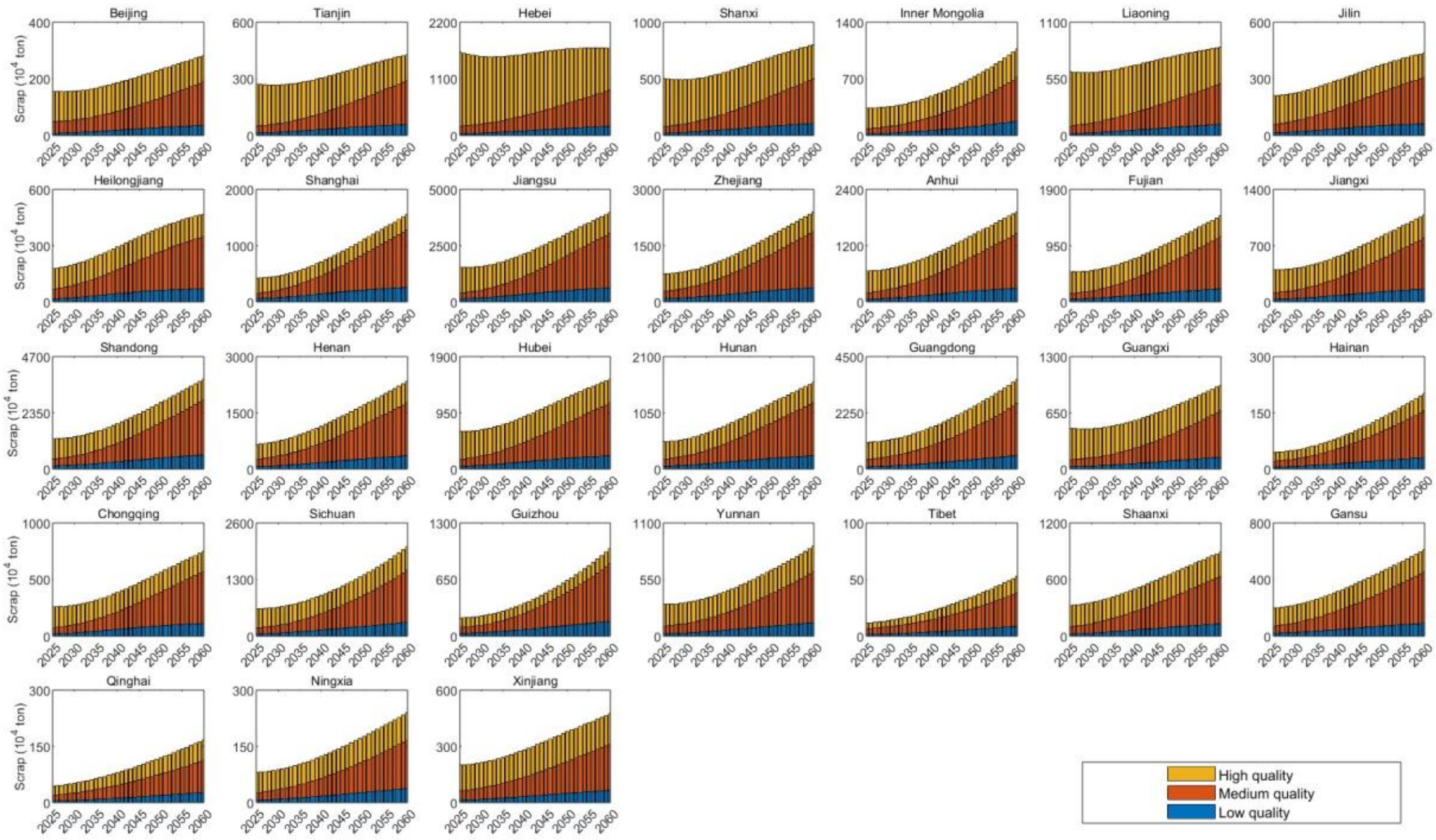

Figure S23. The recycled scrap in each province in baseline scenario

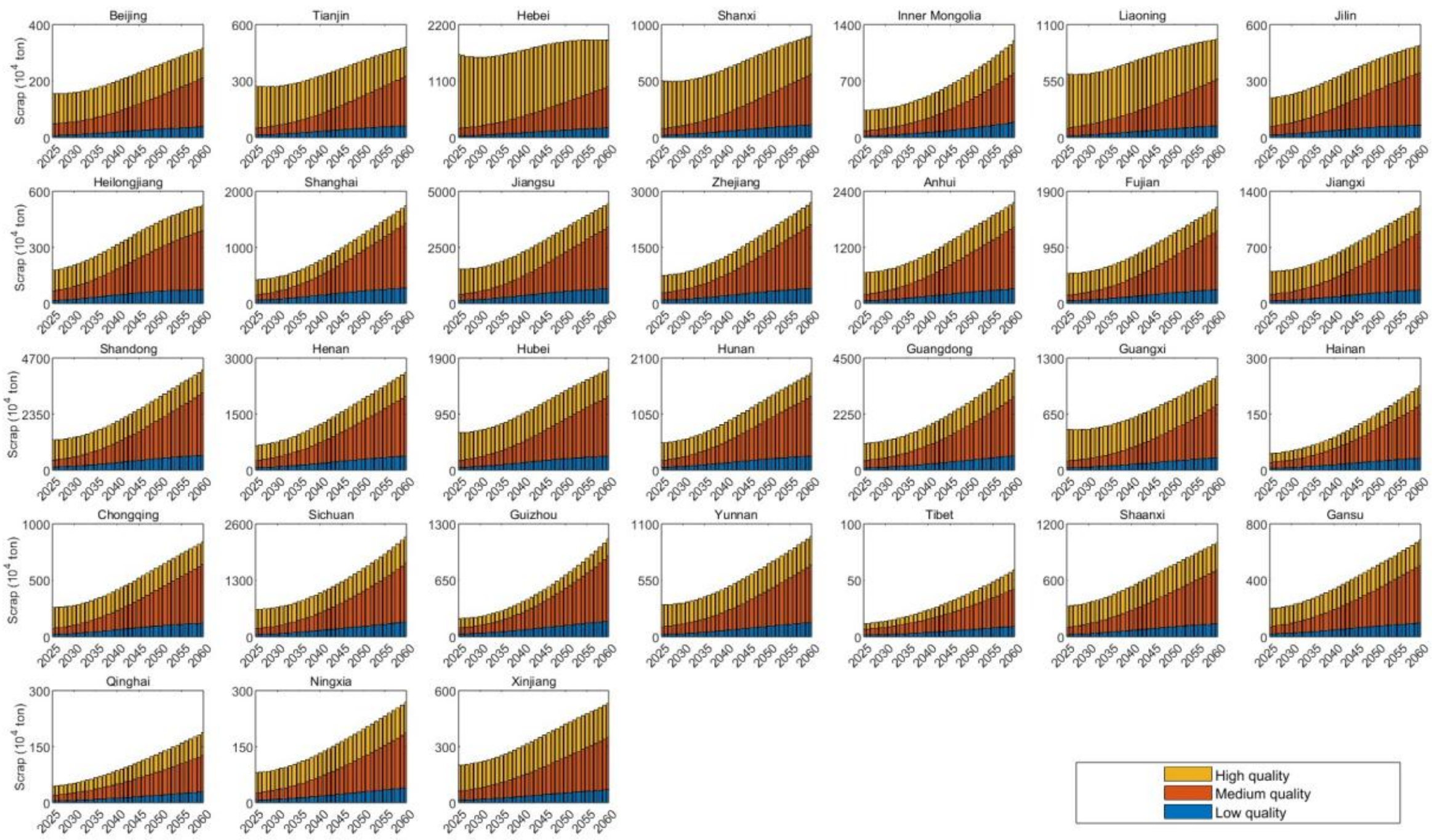

Figure S24. The recycled scrap in each province in accelerated scenario

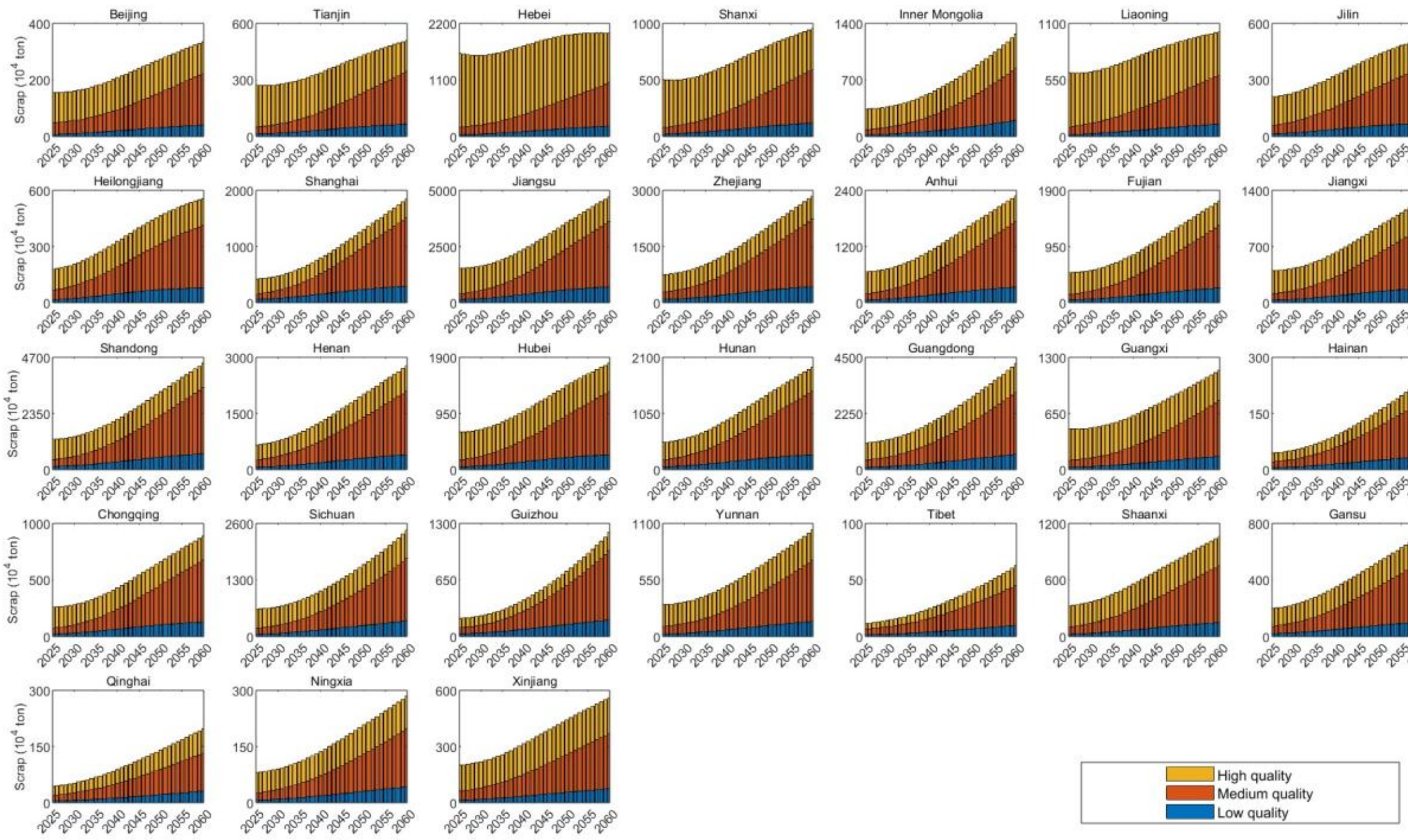

Figure S25. The recycled scrap in each province in ambitious scenario

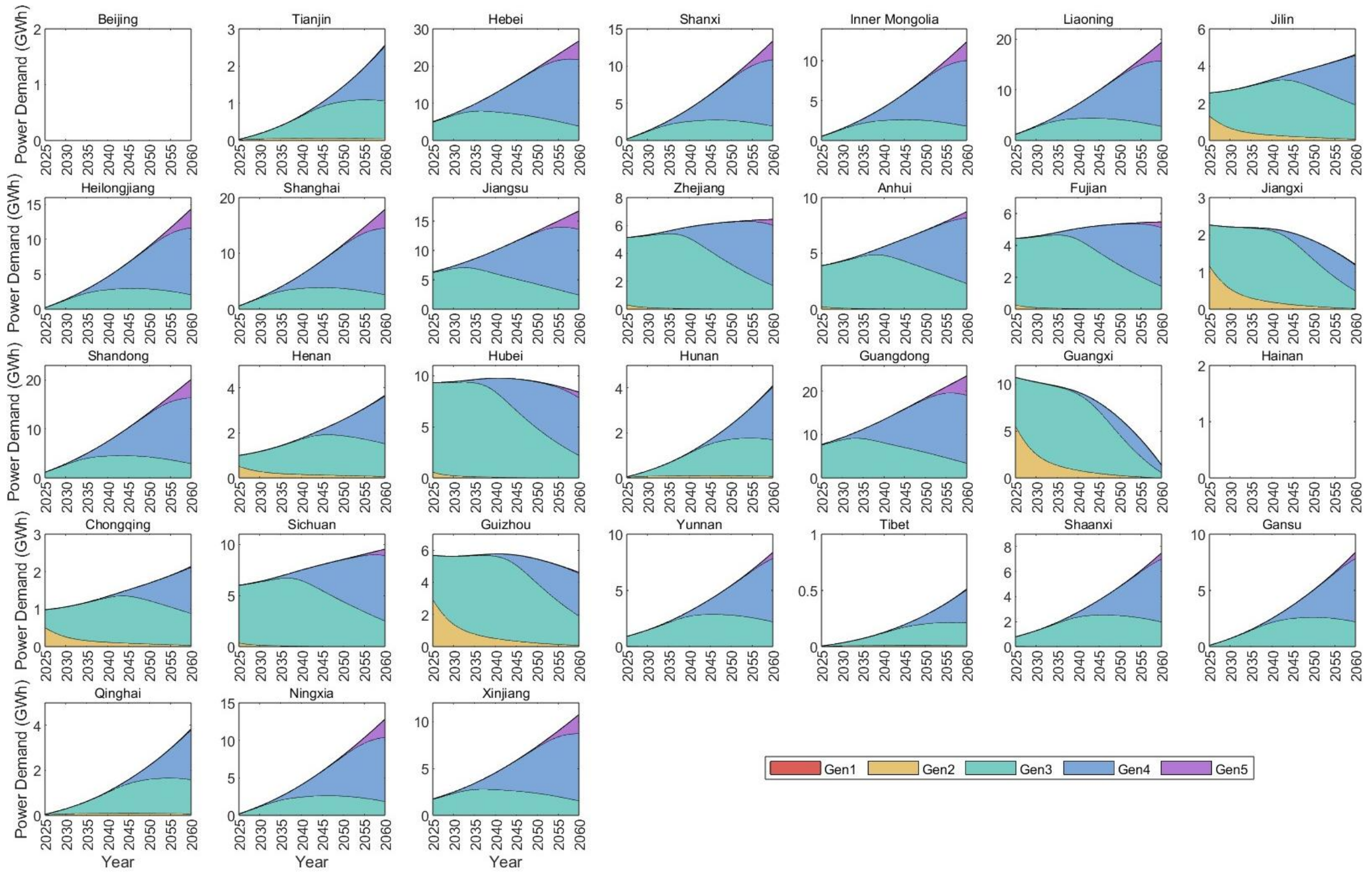

Figure S26. The power demand of EAF in each province in baseline scenario

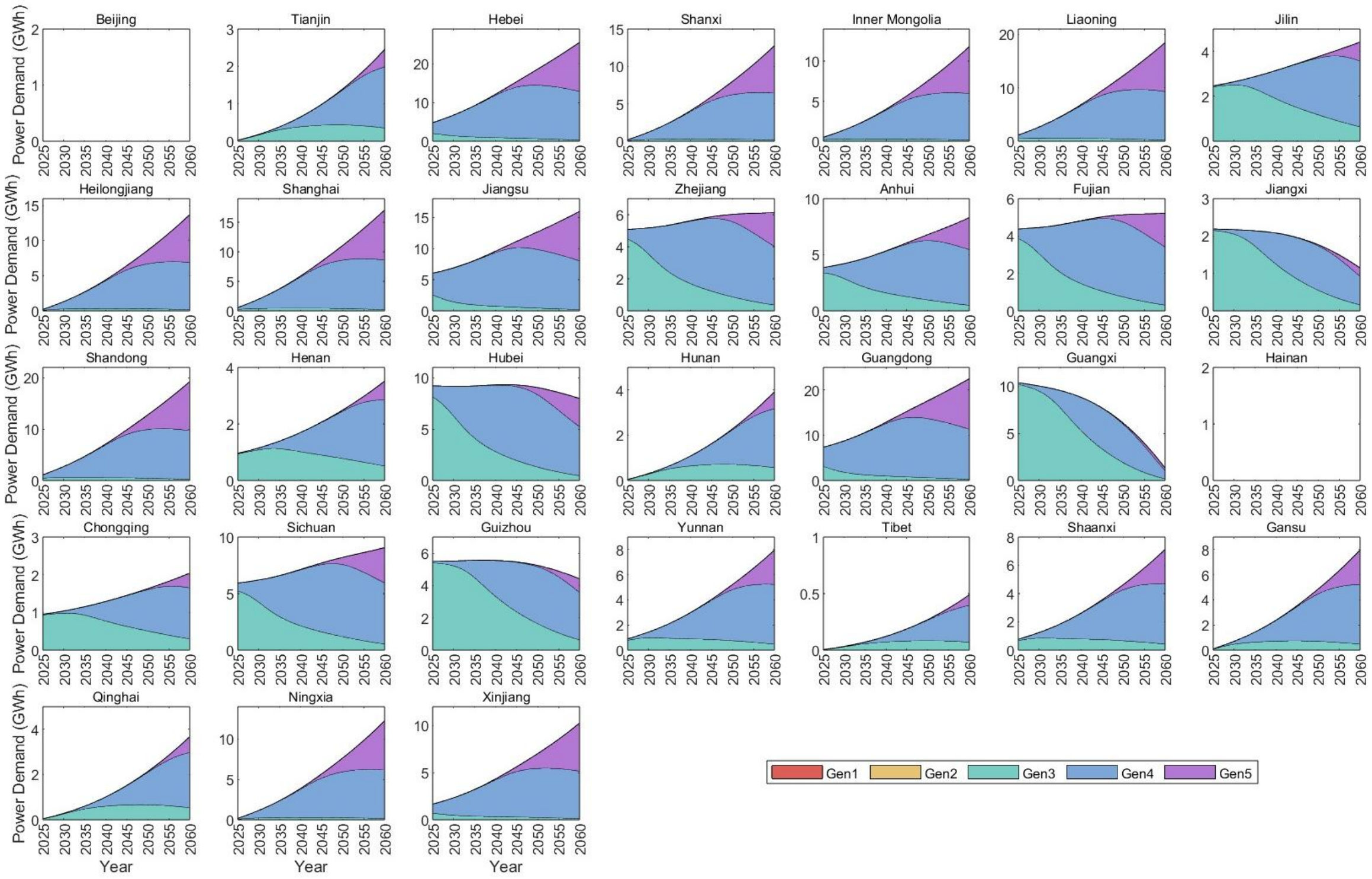

Figure S27. The power demand of EAF in each province in accelerated scenario

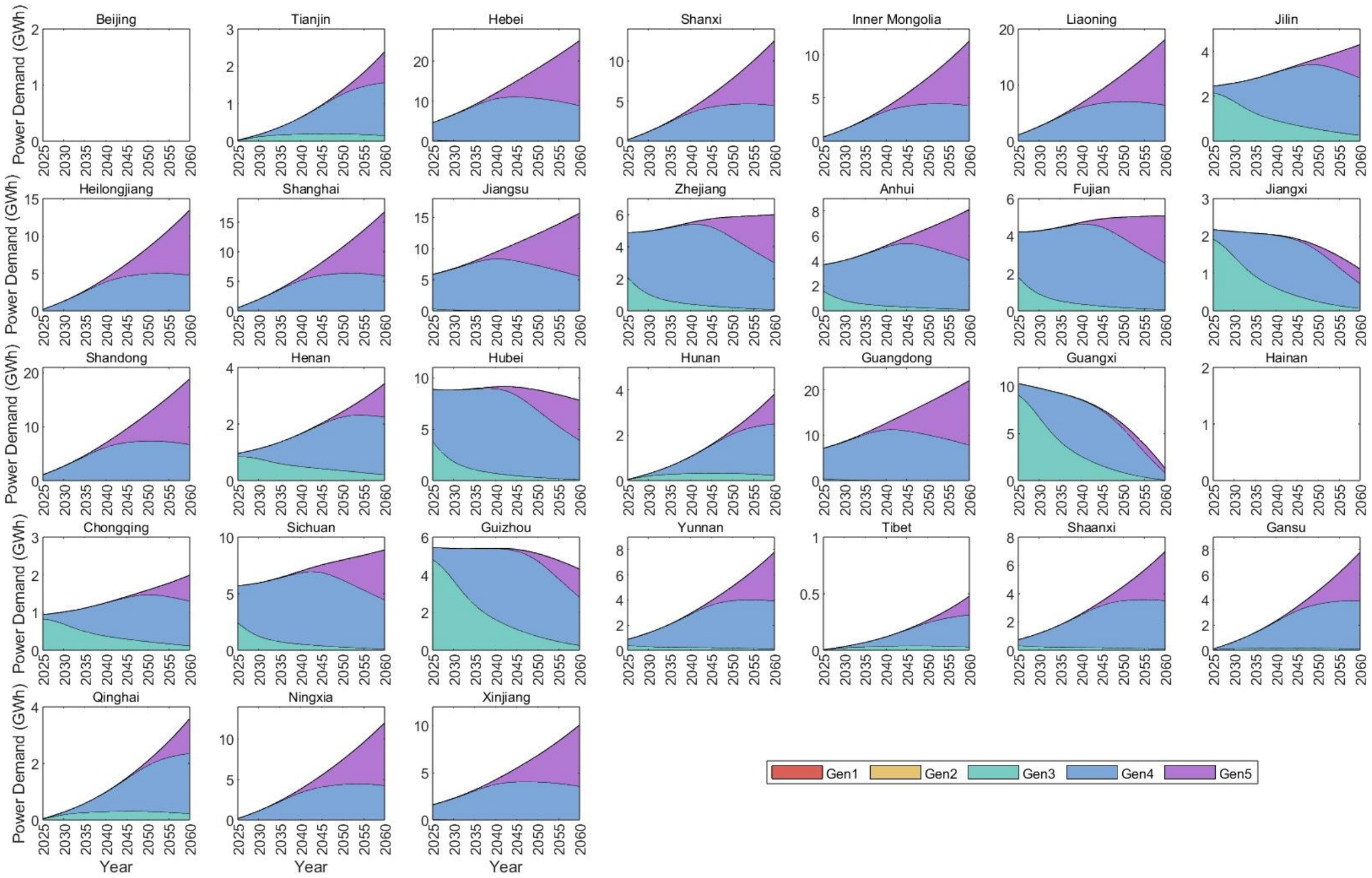

Figure S28. The power demand of EAF in each province in ambitious scenario

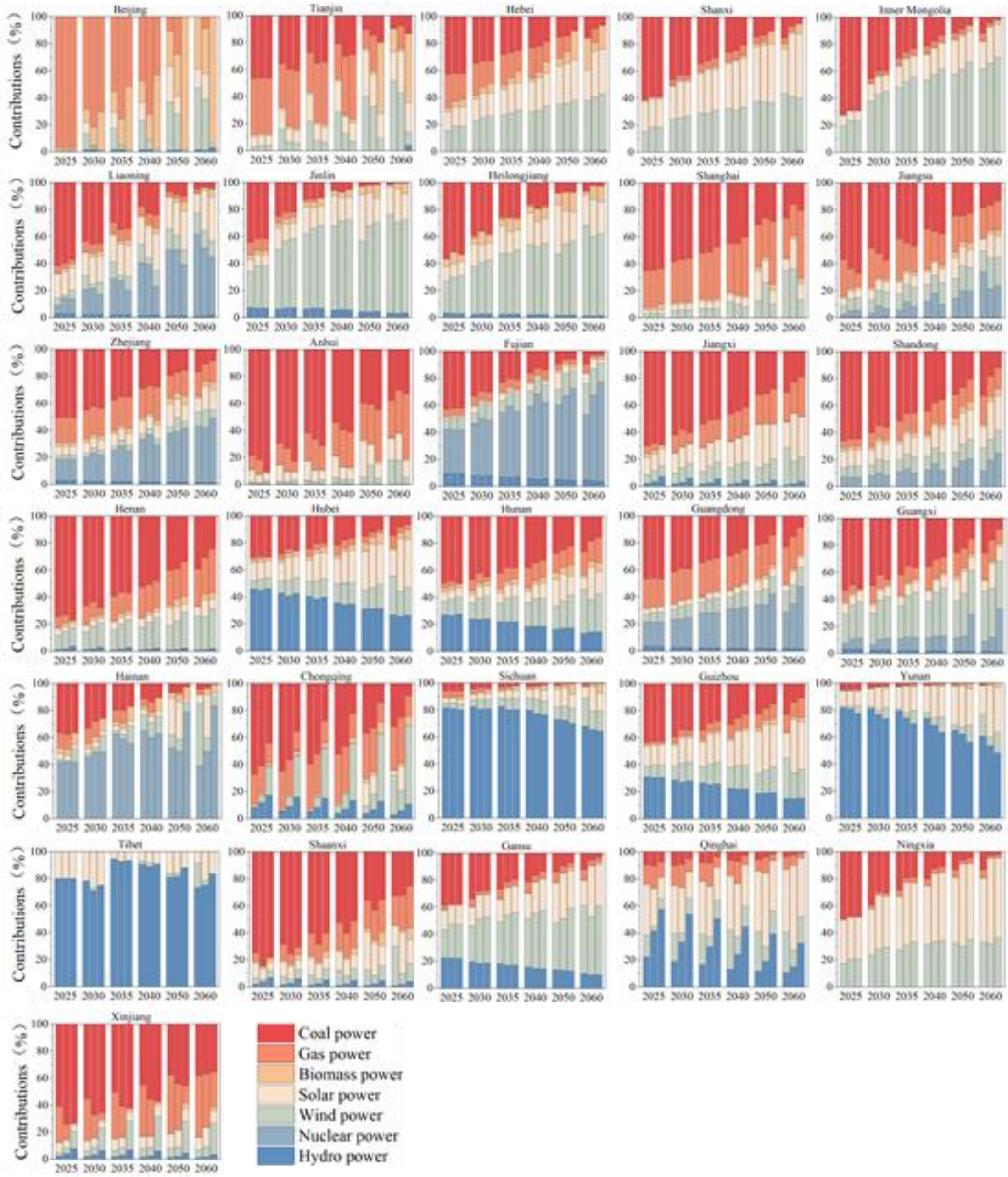

Figure S29. Provincial power generation structure from 2025 to 2060.
(From left to right, the scenarios are baseline scenario, accelerated scenario, and ambitious scenario)

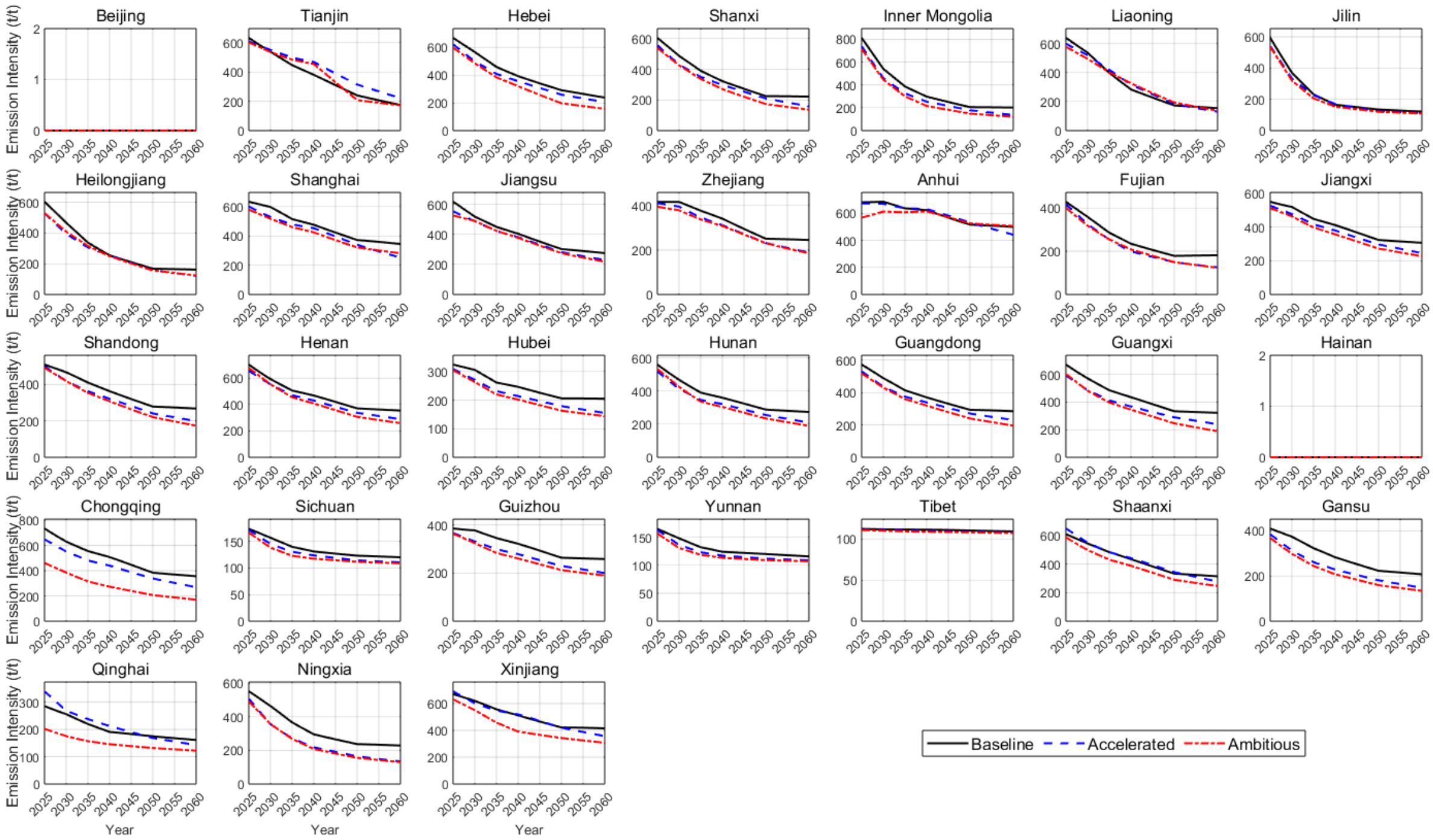

Figure S30. The emission intensities of EAF steelmaking by province

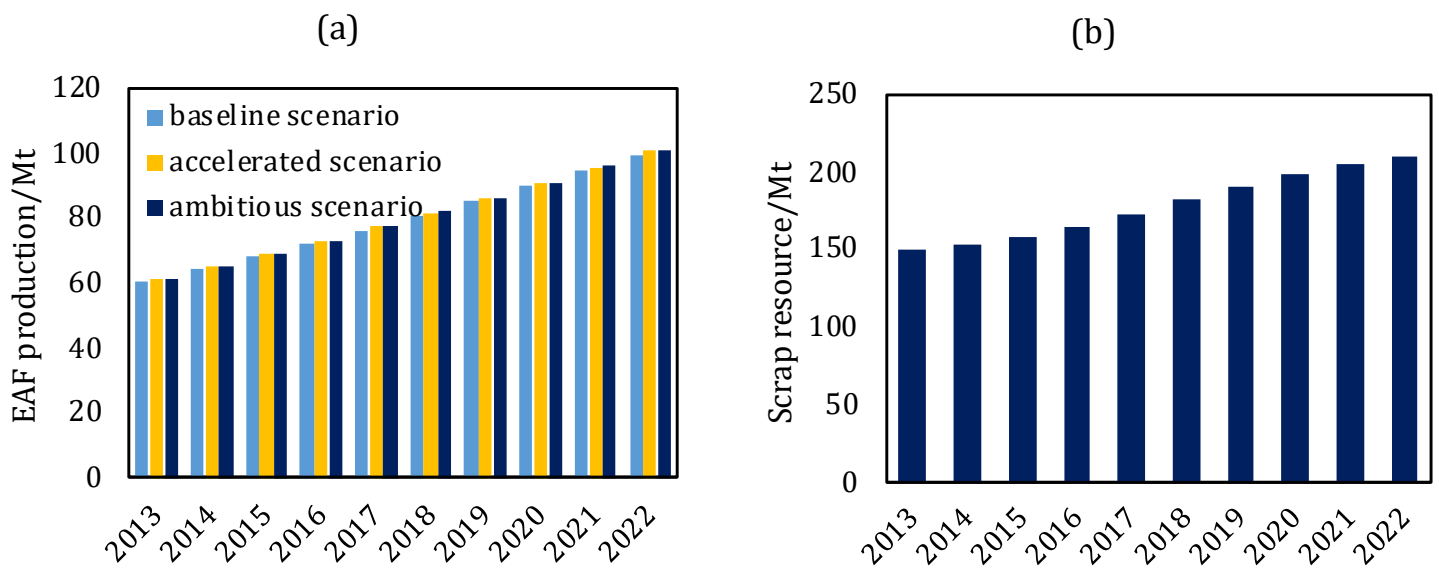

Figure S31. The simulated results from 2013 to 2022. (a) EAF production; (b) Scrap supply
